# In Silico Structure-Guided Optimization and Molecular Simulation Studies of 3-Phenoxy-4-(3-trifluoromethylphenyl)pyridazines as Potent Phytoene Desaturase Inhibitors

**DOI:** 10.3390/molecules26226979

**Published:** 2021-11-19

**Authors:** Lijun Yang, Dawei Wang, Dejun Ma, Di Zhang, Nuo Zhou, Jing Wang, Han Xu, Zhen Xi

**Affiliations:** 1 National Pesticide Engineering Research Center, Department of Chemical Biology, State Key Laboratory of Elemento-Organic Chemistry, College of Chemistry, Nankai University, Tianjin 300071, China; younglj1997@163.com (L.Y.); wangdw@nankai.edu.cn (D.W.); madejun@nankai.edu.cn (D.M.); zhangdi022@126.com (D.Z.); zhounuo2345@163.com (N.Z.); 2 State Key Laboratory of Natural and Biomimetic Drugs, School of Pharmaceutical Sciences, Peking University, No. 38, Xueyuan Road, Beijing 100191, China; wangjing1988@bjmu.edu.cn

**Keywords:** herbicidal activity, molecular design, phytoene desaturase, pyridazine

## Abstract

A series of novel 3-phenoxy-4-(3-trifluoromethylphenyl)pyridazines **2**–**5** were designed, based on the structure of our previous lead compound **1** through the in silico structure-guided optimization approach. The results showed that some of these new compounds showed a good herbicidal activity at the rate of 750 g ai/ha by both pre- and post-emergence applications, especially compound **2a**, which displayed a comparable pre-emergence herbicidal activity to diflufenican at 300–750 g ai/ha, and a higher post-emergence herbicidal activity than diflufenican at the rates of 300–750 g ai/ha. Additionally, **2a** was safe to wheat by both pre- and post-emergence applications at 300 g ai/ha, showing the compound’s potential for weed control in wheat fields. Our molecular simulation studies revealed the important factors involved in the interaction between **2a** and *Synechococcus* PDS. This work provided a lead compound for weed control in wheat fields.

## 1. Introduction

Weeds compete with crops for nutrients, light, and water, and are one of the biggest threats to global food security, causing billions of dollars of economic losses every year [1,2]. Therefore, weed control is crucial to modern sustainable agricultural production [3,4]. There are many ways to control weeds in crop fields, and using herbicides is the most effective and economical approach to decimate weeds [5]. Consequently, the discovery of new herbicides is essential to crop protection [6,7,8]. Phytoene desaturase (PDS) is an important target for the discovery of herbicides [9]. It is a rate-limiting enzyme involved in the biosynthesis of carotenoids, which catalyzes the symmetric desaturation of phytoene to carotene. Inhibition of PDS within plants could block the biosynthesis of carotene and lead to the accumulation of phytoene, which, in turn, would result in the photooxidation of the plant cell. Then, the treated plants would develop a unique whitening symptom on the new leaves, followed by the full whitening effect of the entire leaves, and finally, death [10,11].

**Figure 1:**
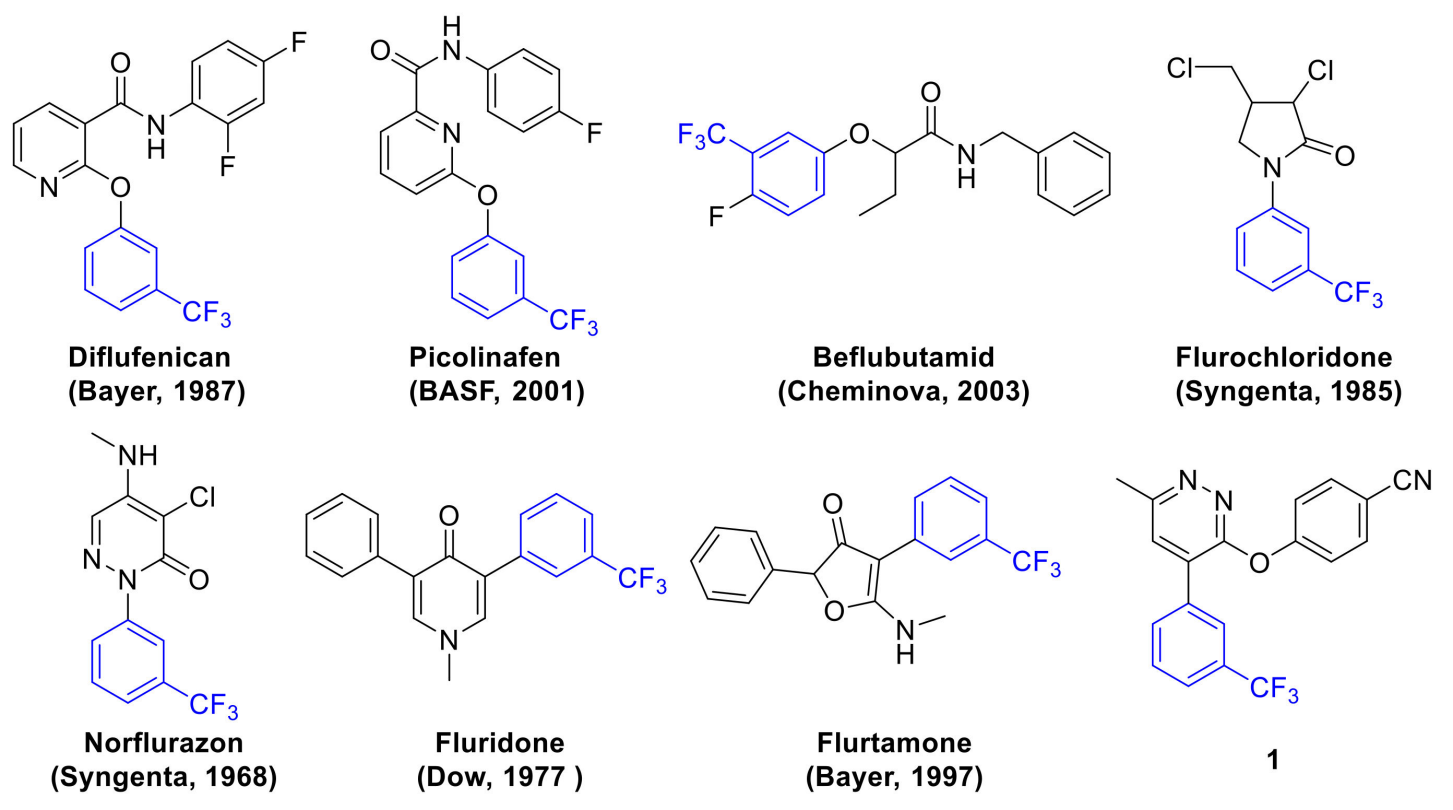
The chemical structures of commercial PDS inhibitory herbicides and the lead compound **1**. The introducing company and year are shown below each herbicide; the common sub-structure of the herbicides and compound **1** are highlighted in blue.

Currently, seven PDS-inhibiting herbicides are being used in the market to decimate weeds in grain fields (Figure 1). Based on the structural features, these herbicides can furtherly be divided into three classes: phenyl–ether (diflufenican, beflubutamid, and picolinafen), *N*-phenyl heterocyclic compounds (norflurazon and flurochloridone), and diphenyl heterocyclic compounds (flurtamone and fluridone) [12]. Diflufenican was developed by Bayer in 1987, and was mainly used for the control of broadleaf weeds in barley and wheat fields by an early post- or pre-emergence application. Norflurazon was developed by Syngenta in 1968. It can control monocotyledon and broadleaf weeds in peanut and cotton fields by a pre-emergence application at 0.5–2 kg ai/ha. Additionally, at the higher application dosage of 1.5–4 kg ai/ha, norflurazon can be used for the management of weeds in non-tilled lands and orchards. Flurtamone was developed by Bayer in 1997; similar to other PDS herbicides, it can also provide a wide range of broadleaf weed control in some commercial crops, such as cotton and sunflower, as well as in grain fields, at the rates of 250–375 g ai/ha.

**Figure 2:**
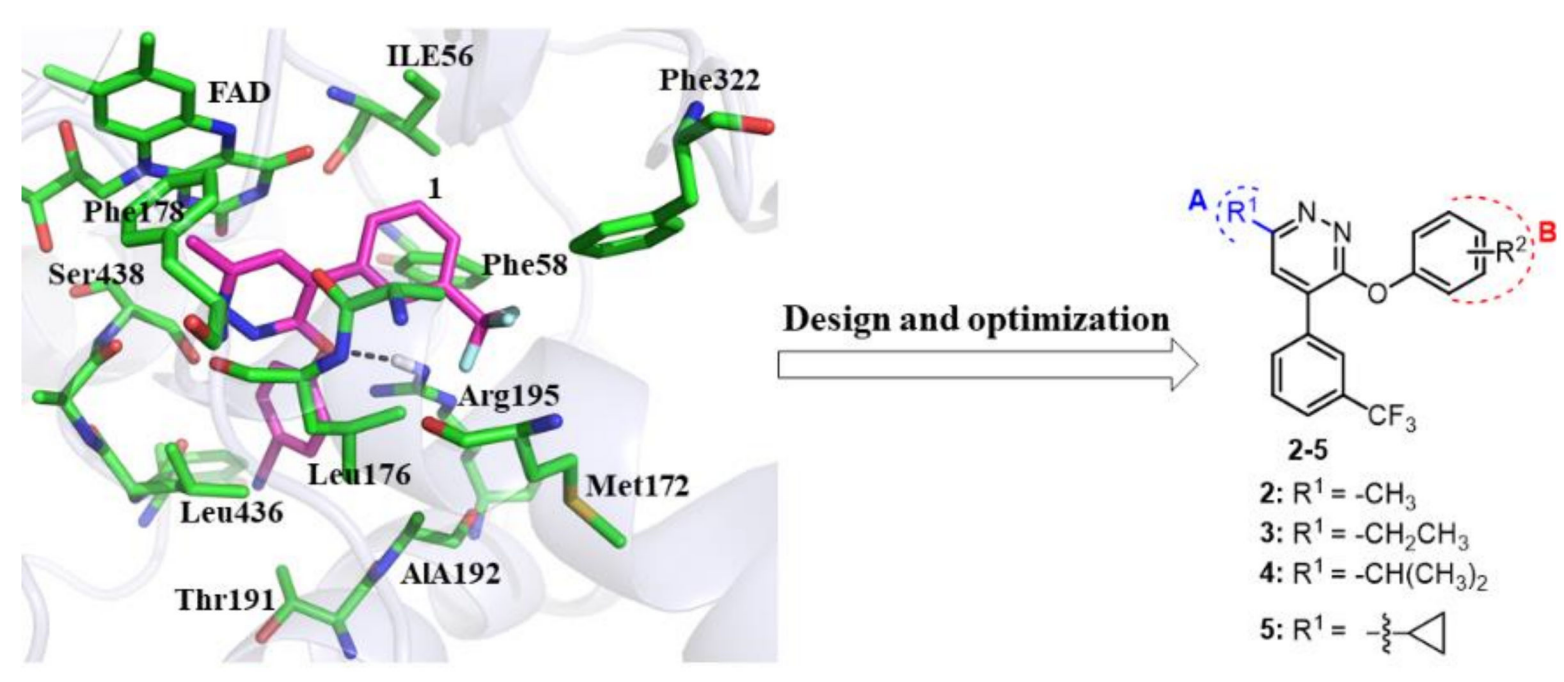
The discovery process of compounds **2**–**5**. The simulated binding mode of compound **1** with *Synechococcus* PDS is shown in cartoon mode, the structure of **1** is shown in the magenta sticks, and the key residues in the active site are shown in the green sticks.

After comparing the structures of the commercial PDS herbicides, we found that they all contain the *m*-trifluoromethyl phenyl group as a common substructure (Figure 1), suggesting that this group has a vital interaction with their target enzyme [13]. In 2017, Brausemann et al., reported the crystal structure of *Oryza sativa* PDS 4-trifluoromethyl phenyl moietyin complex with norflurazon [14]. They found that norflurazon bound to the catalytic site of OsPDS, and that the *m*-trifluoromethyl phenyl group of norflurazon was located at the hydrophobic channel of the protein, and had several important hydrophobic interactions with the surrounding Ala280, Phe423, and Leu421. These findings explained why the *m*-trifluoromethyl phenyl group was important for PDS herbicides. Previously, we have designed a series of 3-(phenoxy)-6-methyl-4-(3-trifluoromethylphenyl)pyridazines; among them, compound **1** (Figure 1) showed good herbicidal activity at the rate of 750 g ai/ha. To obtain a more detailed structure–activity relationship (SAR) of this series of compounds, as well as to obtain new compounds with improved performances, herein, we performed molecular simulation studies on compound **1** with *Synechococcus* PDS, and designed a new series of 3-phenoxy-4-(3-trifluoromethylphenyl)pyridazines **2**–**5** through an in silico structure-guided optimization approach (Figure 2). The systematic SAR studies led to the discovery of a more potent lead compound **2a**. Furthermore, to reveal the vital factors in the binding of **2a** with *Synechococcus* PDS, we performed a systematic molecular simulation study of the two molecules.

## 2. Results

### 2.1. Design of Compounds ***2***–***5***

Previously, we have found that compound **1** was a potent PDS inhibitor, but its herbicidal potency still requires further improvement. In this work, we intended to make an additional structural modification of compound **1,** based on the results of the molecular simulation study of **1** with *Synechococcus* PDS. First, we built the 3D structure of Synechococcus PDS through homologous modeling, using MODELER 9v19, based on the structure of OsPDS [15]. Then, we docked compound **1** to the active site of *Synechococcus* PDS using Autodock 4 software [16]. The results indicated that compound **1** was located at the catalytic site of *Synechococcus* PDS (Figure 2), which was similar to that of the norflurazon–OsPDS system [14]. It was found that the *m*-trifluoromethyl phenyl group of compound **1** was located in the hydrophobic pocket, which was composed of Ile56, Ala175, Phe322, and Phe58. Additionally, the trifluoromethyl group could form an alkyl–π interaction with Phe322 and Phe58, and an alkyl–alkyl interaction with Ala175 and Leu176; the benzyl group could form a T–π interaction with Phe58, and these key interactions may explain why the *m*-trifluoromethyl phenyl group was dispensable for activity. The methyl group of compound **1** was situated at the hydrophobic pocket, and was mainly composed by the isoalloxazine ring of FAD, Phe178, and Val403. The 4-cyano-phenyl group of compound **1** was also located at the hydrophobic cavity, suggesting that the modification of this group may improve the hydrophobic interactions of ligands and the surrounding residues. In addition, the pyridazine ring could form favorable π–π interactions with the isoalloxazine ring of FAD, and the oxygen group had a hydrogen-bonding interaction with Arg95.

Based on the above discussions, we intended to fix the highly conserved *m*-trifluoromethyl phenyl group and modify the methyl and 4-cyano-phenyl groups of compound **1,** to improve the binding affinity between ligands and the protein. Accordingly, compounds **2**–**5** were designed (Figure 2).

### 2.2. Chemistry

**Scheme 1:**
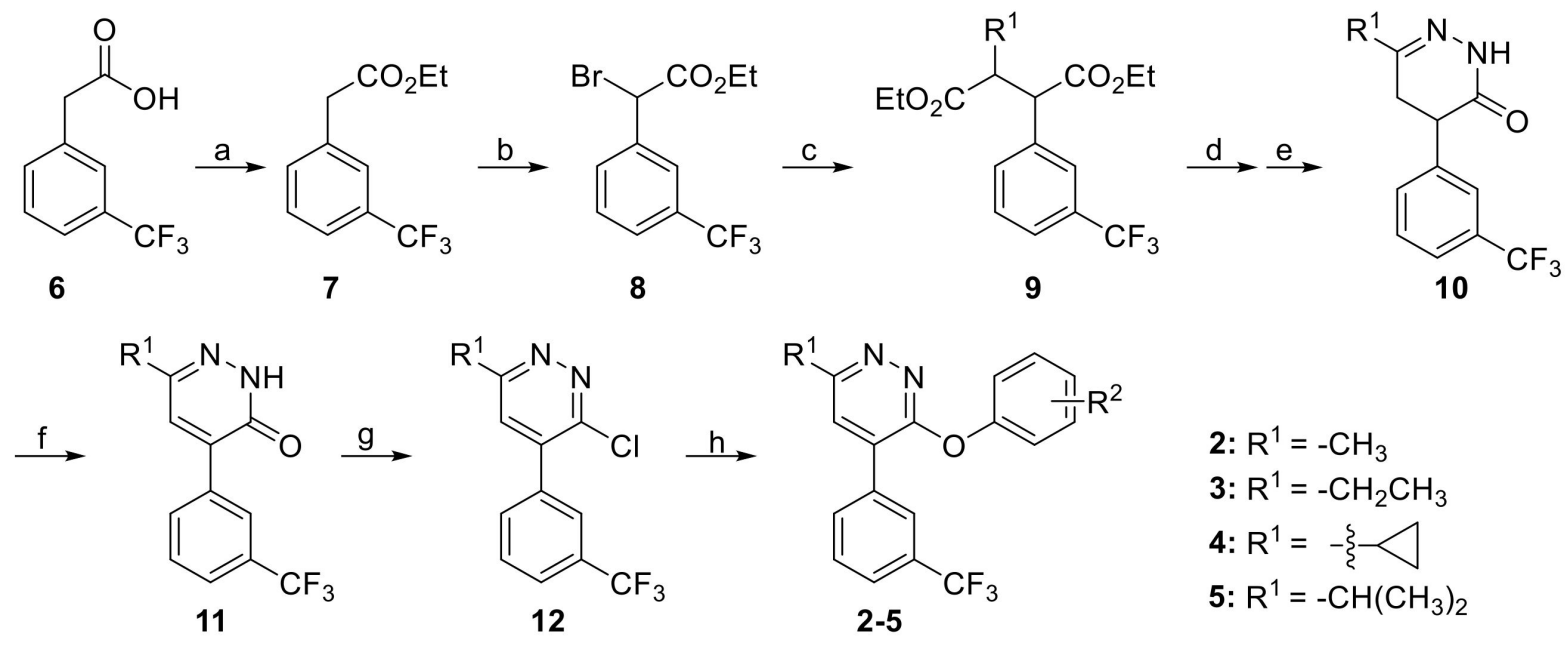
Synthetic routes of compounds **2**–**5**. (**a**) EtOH, H_2_SO_4_, reflux, (**b**) NBS, AIBN, CCl_4_, reflux; (**c**) R^1^COCH_2_CO_2_Et, K_2_CO_3_, CH_3_CN, reflux; (**d**) NaOH, EtOH-H_2_O, reflux; (**e**) N_2_H_4_·H_2_O, EtOH, reflux; (**f**) K_2_CO_3_, DMSO, 60 °C; (**g**) POCl_3_, 60 °C; (**h**) substituted phenols, 130 °C.

To obtain new compounds for the subsequent bioactivity study relatively quickly, we designed synthetic routes that could support substrate expansion at the last step of the reaction. As illustrated in Scheme 1, the newly designed compounds **2**–**5** were prepared, starting from the ester reaction of 2-(3-(trifluoromethyl)phenyl)acetic acid **6** with ethanol, using concentrated H_2_SO_4_ as a catalyst under reflux conditions. The resulting intermediate **7** reacted with the *N*-bromosuccinimide (NBS) in the refluxing of the CCl_4_ solution, which provided the bromide-substituted intermediate **8**. Then, compound **8** reacted with the (substituted) ethyl acetoacetate in CH_3_CN, with K_2_CO_3_ as the base; this afforded the intermediates **9a**–**d**. Without purification, the ethoxycarbonyl groups of **9a**–**d** were hydrolyzed under base conditions which were provided the corresponding acids. Without purification, the acids were directly reacted with N_2_H_4_·H_2_O in ethanol, under refluxing, which afforded the intermediates **10a**–**d**. Subsequently, two hydrogen atoms in the 4,5-dihydropyridazin-3(2*H*)-one ring of **10** were removed by K_2_CO_3_, using DMSO as a reaction solution, at 60 °C. The resulting pyridazin-3(2*H*)-one derivatives, **11a**–**d**, reacted with POCl_3_ at 60 °C, which gave the corresponding intermediates **12a**–**d** in 30–70% yields. Initially, we tried to prepare the compounds **2**–**5** using dimethyl formamide (DMF), CH_3_CN, acetone, dimethyl sulfoxide (DMSO), or *N*-methylpyrrolidone (NMP) as solvents, either under base conditions (using K_2_CO_3_, Cs_2_CO_3_, or NaOH) at room temperature, or under reflux conditions. However, we found that none of these combinations achieved the desired compounds, due to the low conversation rate of the starting material. We, therefore, inferred that the solvent and base might be averse to the reaction. Finally, the preparation of compounds **2**–**5** was accomplished by heating the intermediates **12** with substituted phenols at 130 °C under nitrogen, and the target compounds were obtained in 20–85% yields (Table S1).

### 2.3. Herbicidal Activity and SAR

**Figure 3:**
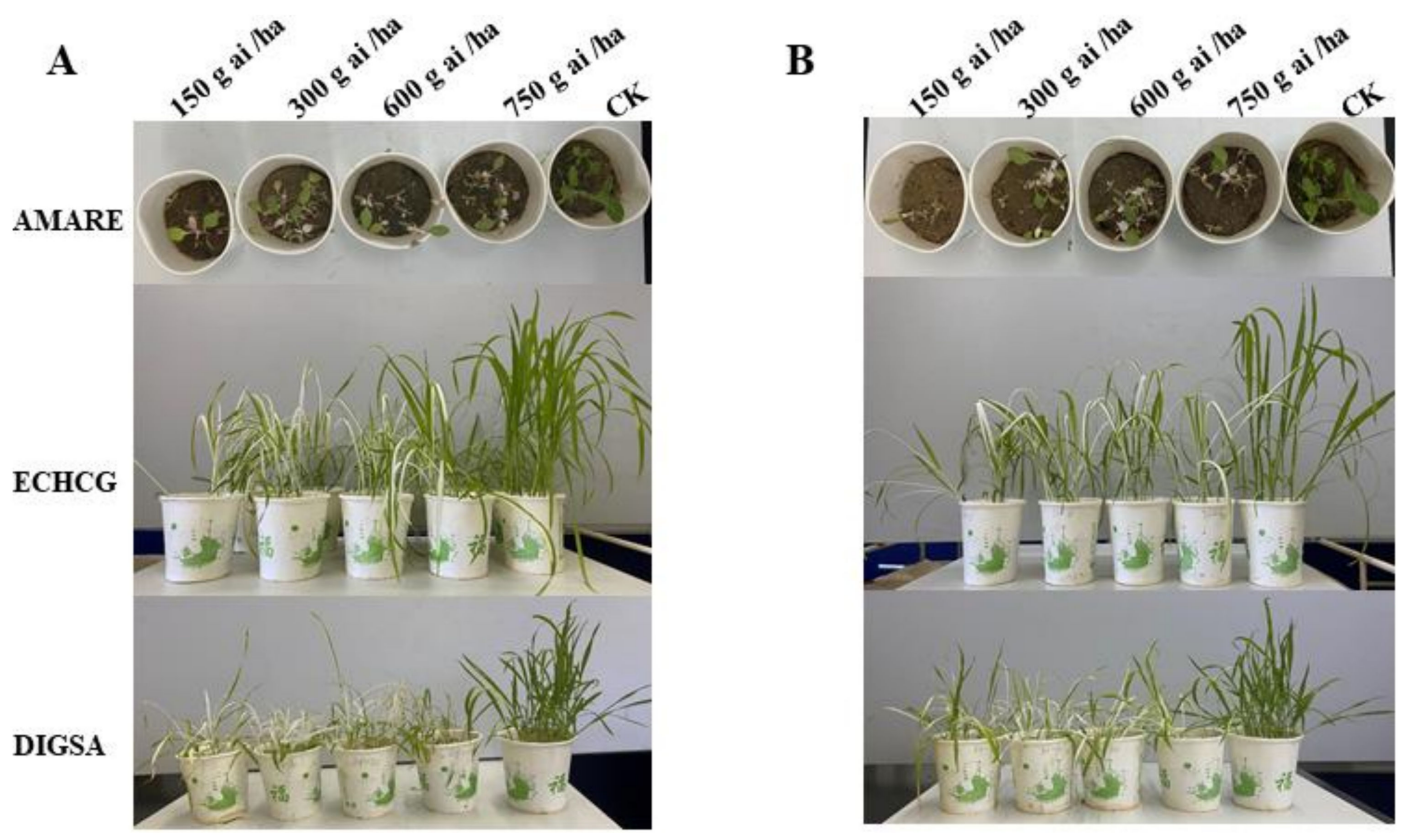
The injury symptoms of *Eclipta prostrata* (ECLPR), *Amaranthus retroflexus* (AMARE), and *Digitaria sanguinalis* (DIGSA) after treatment with **3h** (**A**) and diflufenican (**B**) at a dosage range from 150 to 750 g ai/ha by a post-emergence application, the photos were taken at 20th day after treatment.

The post- and pre-emergence herbicidal potency of the newly synthesized compounds **2**–**5** were tested against six weeds, as was the compound diflufenican, as well as our previous lead compound **1**, which was was selected as the positive control. The post-emergent herbicidal potency of these compounds is shown in Table 1, and the pre-emergent herbicidal activity is shown in Table S2. The results regarding the herbicidal activity showed that most of the synthesized compounds **2**–**5** showed good to excellent weed control toward the tested weeds. Some compounds, such as **2a** and **3h**, exhibited excellent herbicidal activity higher than that of the lead compound **1**, and comparable to diflufenican by both pre- and post-emergence application. Additionally, we found that the synthesized compounds showed similar whitening symptoms on the leaves of the treated weeds. For example, as shown in Figure 3, compound **3h** exhibited the same whitening symptoms on the leaves of AMARE, ECHCG, and DIGAS as that of diflufenican, suggesting that compound **3h** might also inhibit the PDS enzyme in planta.

Initially, we kept the R^1^ as a -CH_3_ and optimized the R^2^ substitution at the phenoxy group. Previously, we found that installing the electron-donating groups at R^2^ was detrimental to the compounds’ bioactivity [17]. Therefore, in the current work, we mainly introduced the electron-withdrawing groups at R^2^. As depicted in Table 1, introducing the -CF_3_ group at the 4-position of the benzene ring led to compound **2a**, which showed excellent herbicidal activity (over 80% that of control) against the six tested weeds at the dosage of 750 g ai/ha, and displayed significantly improved potency than that of diflufenican and compound **1**. Substituting the fluorine atom at the 3-position of the benzene ring (**2b**) was found to be unfavorable to herbicidal activity. In contrast, installing another fluorine atom at the 3-position could improve the herbicidal potency (**2c**), indicating that the electron-withdrawing group at the 4-position was crucial to activity. Switching the 4-fluorine atom at the benzene ring of **2c** to the chlorine atom was beneficial to herbicidal activity (**2d** > **2c**). Interestingly, changing the 4-fluorine atom at **2c** to a bromine atom almost did not affect the herbicidal potency (**2e** ≈ **2e**). In addition, adding another fluorine atom at the 5-position of the benzene ring of **2c** further improved the potency (**2h** > **2c**). Next, we changed the methyl group at the R^1^ to an ethyl group, and synthesized the compounds **3a**–**i** (Scheme 1). It was observed that there was some variation in the herbicidal activity of compounds **2** and **3**. For example, compound **3a** showed a decreased herbicidal potency relative to its mother compound **1a**, while **3h** showed an improved herbicidal activity than **2h**. In most cases, switching the ethyl groups of compounds **3a**–**i** to the cyclopropyl group decreased the herbicidal activity (**4a**–**i**). However, changing the cyclopropyl groups of **4a**–**I** to the isopropyl groups resulted in the nearly complete loss of herbicidal potency (**5a**–**i**). These results indicated that the substitution of the steric bulky groups at R^1^ could reduce the herbicidal activity. Therefore, the effect of R^1^ substitution on the post-emergence herbicidal activity decreased in the order of -CH_3_, -CH_2_CH_3_ > cyclopropyl group > -CH(CH_3_)_2_.

In most cases, the pre-emergence herbicidal activity SAR trends for the compounds **2**–**5** were similar to their post-emergence herbicidal activity that was discussed in the above paragraph. For example, as shown in Table S2, the compounds **2a**–**h** (R^1^ = -CH_3_) and **3a**–**i** (R^1^ = -CH_2_CH_3_) showed a higher herbicidal activity than the compounds **4a**–**i** (R^1^ = cyclopropyl group) and **5a**–**i** (R^1^ = -CH(CH_3_)_2_) (**2**, **3** > **4** > **5**). Additionally, the compound **2a** showed a strong pre-emergence herbicidal activity comparable to that of diflufenican, while **3h** showed less potency than diflufenican.

### 2.4. Crop Selectivity

Compound **2a** showed higher herbicidal potency than diflufenican, at the rates of 300–750 g ai/ha by the post-emergence application, as well as a comparable potency to that of diflufenican by the pre-emergence application. Therefore, to explore whether **2a** could be a potential lead compound or not, we carried out further crop selectivity tests of **2a** against six representative crops. As shown in Table 2, at the rate of 300 g ai/ha by the post-emergence application, the compound **2a** showed high selectivity to wheat. At the same time, diflufenican was not as safe as **2a** to wheat in this experimental condition. Additionally, at 300 g ai/ha, both **2a** and diflufenican showed excellent safety towards the six kinds of crops by the pre-emergence application. Collectively, our results showed that **2a** had the potential to develop as a lead herbicide for wheat fields by both pre- and post-emergence application.

### 2.5. Synechococcus PDS Binding Affinity

To understand whether our newly synthesized compounds could bind to the PDS in vitro or not, the compounds **2a** and **3h,** which had good herbicidal activity, were selected for further *Synechococcus* PDS binding affinity experiments, using a surface plasmon resonance (SPR) assay [18]. We found that **2a** and **3h** could bind to the recombinant *Synechococcus* PDS protein which was immobilized on the sensor chip, and the *K*_D_ values for **2a** and **3h** were determined to be 7.08 and 63.2 μM, respectively. To our surprise, the *K*_D_ value of **2a** was even higher than that of diflufenican (70.8 μM) and **the** lead compound **1** (43.5 μM). Together, our results indicated that **2a** decimates weeds through the inhibition of PDS *in planta*.

### 2.6. Molecular Simulation Studies

**Figure 4:**
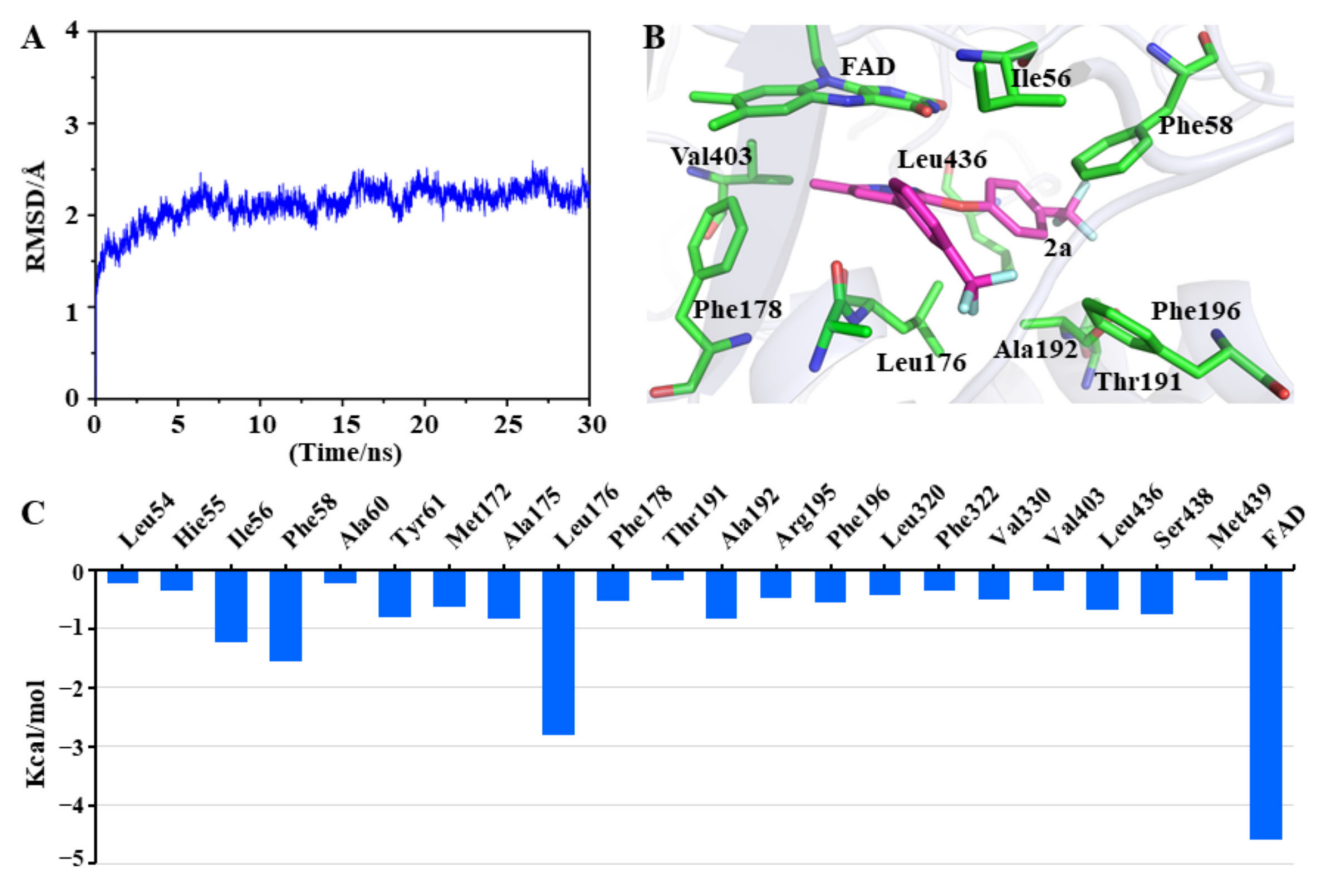
Molecular dynamic simulation study of the **2a**-*Synechococcus* PDS system. (**A**) The root-mean-square deviation (RMSD) analysis of the C, CA, and N atoms during the MD simulation. (**B**) The binding mode of **2a** to *Synechococcus* PDS at the last trajectory of MD simulation. (**C**) Decomposing of the binding free energy of the **2a**–*Synechococcus* PDS complex, in the last 10 ns of the MD trajectories, using MM-GBSA.

To understand the binding details of the synthesized inhibitors to *Synechococcus* PDS, we carried out molecular dynamic (MD) simulation studies for the representative compound **2a** with *Synechococcus* PDS. First, we docked **2a** to the active site of *Synechococcus* PDS using Autodock 4 software, and the best binding mode of **2a** to the protein was selected, based on the binding score [19]. Next, we performed a 30 ns MD simulation of the **2a–***Synechococcus* PDS complex using the Amber14 program (Figure 4A). Then, we calculated the binding free energy (Δ*G*_bind_) of **2a** to *Synechococcus* PDS, using the MM-PBSA method, from the last ten ns of the MD trajectory. As shown in Table S3, the Δ*G*_bind_ was calculated to be −19.37 kcal/mol, and the van der Waals (Δ*E*_VDW_ = −52.04 kcal/mol) energy contributed a larger proportion to the Δ*G*_bind_ than the electrostatic (Δ*E*_ele_ = −12.95 kcal/mol) energy.

Additionally, we extracted the binding mode of **2a** to *Synechococcus* PDS from the MD trajectory. As shown in Figure 4B, at the 30th ns, compound **2a** was still located at the active site of *Synechococcus* PDS, and several conserved interactions were observed to occur between **2a** and *Synechococcus* PDS. For example, the isoalloxazine ring of FAD could form a π–π interaction with the pyridazine ring of **2a**, Phe58 had a T-shaped π interaction with the benzene ring of the 4-trifluoromethyl phenyl moiety of **2a**, and the 3-trifluoromethyl phenyl moiety of **2a** had hydrophilic interactions with the surrounding Phe196, Ile56, and Ala175.

To understand the ligand–protein interaction from the energy perspective, we performed a binding free energy decomposition of the **2a**–*Synechococcus* PDS system, using the MM-GBSA method. The results indicated that, among the resides of *Synechococcus* PDS (Figure 4C), FAD contributed the most significant proportion of the binding free energy (Δ*G*_bind_ = −4.59 kcal/mol), suggesting that the π–π interaction between the ligand and FAD is crucial to the activity. In addition, Leu176 was also found to contribute a large proportion to the binding energy (Δ*G*_bind_ = −2.81 kcal/mol), showing the importance of the 3-trifluoromethylphenyl group to the binding free energy value; this observation can also explain why the 3-trifluoromethylphenyl function group was indispensable to the herbicidal activity observed. Other residues, such as Ile56 (Δ*G*_bind_ = −1.23 kcal/mol), Phe58 (Δ*G*_bind_ = −1.57 kcal/mol), Tyr61 (Δ*G*_bind_ = −0.8 kcal/mol), Ala175 (Δ*G*_bind_ = −0.83 kcal/mol), and Ala192 (Δ*G*_bind_ = −0.82 kcal/mol) also made relatively large contributions to the binding free energy.

## 3. Discussion

In this research, we performed molecular modeling studies of our previously discovered lead compound **1** with *Synechococcus* PDS, and designed the compounds **2**–**5**. Our herbicidal activity results showed that some newly synthesized compounds displayed a good herbicidal activity against the tested weeds at 750 g ai/ha by both pre- and post-emergence applications. Promisingly, compound **2a** showed a higher post-emergence herbicidal activity than diflufenican at the rates of 300–750 g ai/ha, and a comparable pre-emergence herbicidal activity to that of diflufenican at 300–750 g ai/ha. Most importantly, **2a** was safe to wheat by both pre- and post-emergence applications at 300 g ai/ha, suggesting the great potential for it to develop as a herbicide for wheat fields. Additionally, we found that **2a** (*K*_D_ = 7.08 μM) bound more tightly to the *Synechococcus* PDS than did lead compound **1** (*K*_D_ = 43.5 μM) and diflufenican (*K*_D_ = 70.8 μM). The results of our molecular simulation studies indicated that **2a** could bind to the active site of *Synechococcus* PDS, and that the van der Waals interaction between ligand and protein is important to its activity. Our present study not only provided a lead compound for weed control but also be useful to explain why the 3-trifluoromethylphenyl functional group is crucial to the bioactivity of PDS inhibitors.

## 4. Materials and Methods

### 4.1. Synthetic Chemistry

The chemicals used in this study were all commercially available, unless otherwise specified. Silica gel (200–300 mesh) was used for column chromatography. The ^1^H and ^13^C NMR spectra of all the synthesized compounds were recorded on a Bruker Avance II 400 MHz spectrometer. The HRMS spectra data were obtained on a Varian 7.0T FTICR-MS spectrometer.

#### 4.1.1. Preparation of the Intermediates **7**–**12**

The synthetic routes for the intermediates **7**–**12** were the same as for our previous reports [13,17,20]. The details are shown in the Supporting Information.

#### 4.1.2. General Route to Prepare Compounds **2**–**5**

A mixture of 3-chloro-6-(substituted)-4-(3-(trifluoromethyl)phenyl)pyridazines (0.87 mmol) and (substituted)phenol (2.2 mmol) was heated at 130 °C, with stirring for 12 h. Then, the reaction mixture was cooled to room temperature, and CH_2_Cl_2_ (100 mL) was added to dissolve the mixture. The organic layer was washed with 10% NaOH (30 mL) and brine (20 mL), dried over magnesium sulfate, and filtered. The resulting solution was concentrated and purified by column chromatography to afford **2**–**5**.

*6-methyl-3-(4-(trifluoromethyl)phenoxy)-4-(3(trifluoromethyl)phenyl)pyridazine* (**2a**). Yellow solid; yield 85%; ^1^H NMR (400 MHz, acetone-*d*_6_) *δ* 8.16 (d, *J* = 6.4 Hz, 2H), 7.88–7.83 (m, 2H), 7.82–7.76 (m, 3H), 7.49 (d, *J* = 8.6 Hz, 2H), 2.68 (s, 3H). ^13^C NMR (101 MHz, acetone-*d*_6_) *δ* 162.01, 159.16, 158.28, 135.35, 134.12, 131.96, 131.64, 131.32, 130.93, 130.63, 129.90, 127.94, 127.90, 127.86, 127.83, 127.02, 126.98, 126.94, 126.90, 126.86, 122.65, 21.48.HRMS (QFT-ESI) calcd for C_19_H_12_F_6_N_2_O [M + H]^+^ 399.0927, found: 399.0930.

*3-(3-fluorophenoxy)-6-methyl-4-(3-(trifluoromethyl)phenyl)pyridazine* (**2b**). Yellow oil; yield 60%; ^1^H NMR (400 MHz, acetone-*d*_6_) *δ* 8.15 (d, *J* = 9.2 Hz, 2H), 7.81–7.79 (m, 3H), 7.45–7.43 (m, 1H), 7.15–7.07 (m, 2H), 7.03–6.97 (m, 1H), 2.66 (s, 3H). ^13^C NMR (101 MHz, acetone-*d*_6_) *δ* 163.82, 161.39, 160.59, 157.40, 155.00, 154.94, 134.01, 132.64, 130.16, 130.07, 129.29, 129.11, 128.22, 116.61, 116.57, 111.09, 110.87, 108.51, 108.26, 19.96. HRMS (QFT-ESI) calcd for C_18_H_12_F_4_N_2_O [M + H]^+^ 349.0959, found: 349.0953.

*3-(3,4-difluorophenoxy)-6-methyl-4-(3-(trifluoromethyl)phenyl)pyridazine* (**2c**). Yellow solid; yield 85%; ^1^H NMR (400 MHz, acetone-*d*_6_) *δ* 8.19–8.13 (m, 2H), 7.87 (d, *J* = 7.9 Hz, 1H), 7.83–7.76 (m, 2H), 7.45–7.35 (m, 2H), 7.14–7.12 (m, 1H), 2.66 (s, 3H). ^13^C NMR (101 MHz, acetone-*d*_6_) *δ* 152.13, 148.86, 142.30, 142.16, 141.07, 141.04, 140.98, 140.95, 139.84, 139.71, 139.58, 137.30, 137.17, 125.40, 124.14, 121.91, 121.59, 121.27, 120.95, 120.80, 120.59, 119.48, 116.96, 116.92, 116.89, 116.85, 108.95, 108.91, 108.89, 108.85, 108.48, 108.29, 102.51, 102.31, 11.42. HRMS (QFT-ESI) calcd for C_18_H_11_F_5_N_2_O [M + H]^+^ 367.0864, found: 367.0868.

*3-(4-chloro-3-fluorophenoxy)-6-methyl-4-(3-(trifluoromethyl)phenyl)pyridazine* (**2d**). Yellow oil; yield 75%; ^1^H NMR (400 MHz, acetone-*d*_6_) *δ* 8.15 (dd, *J* = 2.6, 1.9 Hz, 2H), 7.89–7.77 (m, 3H), 7.58 (t, *J* = 8.7 Hz, 1H), 7.36 (dd, *J* = 10.2, 2.6 Hz, 1H), 7.17–7.15 (m, 1H), 2.67 (s, 3H). ^13^C NMR (101 MHz, acetone-*d*_6_) *δ* 151.92, 150.10, 149.09, 147.63, 144.78, 144.68, 125.23, 124.09, 121.89, 121.75, 121.57, 121.25, 120.91, 120.58, 119.68, 116.98, 116.95, 116.91, 116.88, 116.84, 116.80, 109.42, 109.39, 107.30, 107.14, 101.63, 101.39, 11.44. HRMS (QFT-ESI) calcd for C_18_H_11_ClF_4_N_2_O [M + H]^+^ 383.0569, found: 383.0571.

*3-(4-bromo-3-fluorophenoxy)-6-methyl-4-(3-(trifluoromethyl)phenyl)pyridazine* (**2e**). Yellow solid; yield 82%; ^1^H NMR (400 MHz, acetone-*d*_6_) *δ* 8.18–8.13 (m, 2H), 7.89–7.77 (m, 3H), 7.75–7.69 (m, 1H), 7.34–7.32 (m, 1H), 7.12–7.10 (m, 1H), 2.68 (s, 3H). ^13^C NMR (101 MHz, acetone-*d*_6_) *δ* 206.31, 161.93, 161.23, 159.09, 158.78, 155.64, 155.54, 135.28, 134.68, 134.14, 131.91, 131.59, 131.27, 131.00, 130.62, 129.82, 127.02, 126.99, 126.95, 126.92, 126.88, 126.84, 123.81, 119.92, 119.89, 111.56, 111.31, 104.74, 104.53, 21.41. HRMS (QFT-ESI) calcd for C_18_H_11_BrF_4_N_2_O [M + H]^+^ 427.0064, found: 427.0066.

*2-fluoro-4-((6-methyl-4-(3-(trifluoromethyl)phenyl)pyridazine-3-yl)oxy)benzonitrile* (**2f**). Yellow solid; yield 75%; ^1^H NMR (400 MHz, acetone-*d*_6_) *δ* 8.16–8.11 (m, 2H), 7.95–7.85 (m, 3H), 7.82–7.77 (m, 1H), 7.46 (dd, *J* = 10.6, 2.2 Hz, 1H), 7.34 (dd, *J* = 8.6, 2.2 Hz, 1H), 2.70 (s, 3H). ^13^C NMR (101 MHz, acetone-*d*_6_) *δ* 165.91, 163.36, 161.49, 160.41, 160.30, 159.81, 135.51, 134.89, 134.02, 131.23, 130.63, 130.17, 127.10, 127.06, 127.02, 126.99, 126.89, 126.85, 126.81, 126.78, 118.92, 118.89, 114.30, 110.58, 110.35, 97.95, 97.80, 21.50. HRMS (QFT-ESI) calcd for C_19_H_11_F_4_N_3_O [M + H]^+^ 374.0911, found: 374.0915.

*3-(4-chloro-2-fluorophenoxy)-6-methyl-4-(3-(trifluoromethyl)phenyl)pyridazine* (**2g**). Yellow solid; yield 78%; ^1^H NMR (400 MHz, acetone-*d*_6_) *δ* 8.21–8.14 (m, 2H), 7.90–7.77 (m, 3H), 7.53–7.40 (m, 2H), 7.33 (d, *J* = 8.7 Hz, 1H), 2.66 (s, 3H). ^13^C NMR (101 MHz, acetone-*d*_6_) *δ* 151.92, 150.10, 149.09, 147.63, 144.78, 144.68, 125.23, 124.09, 121.89, 121.75, 121.57, 121.25, 120.91, 120.58, 119.68, 109.42, 109.39, 107.30, 107.14, 101.63, 101.39, 11.44. HRMS (QFT-ESI) calcd for C_18_H_11_ClF_4_N_2_O [M + H]^+^ 383.0569, found: 383.0563.

*6-methyl-4-(3-(trifluoromethyl)phenyl)-3-(3,4,5-trifluorophenoxy)pyridazine* (**2h**). Yellow oil; yield 65%; ^1^H NMR (400 MHz, acetone-*d*_6_) *δ* 8.15 (d, *J* = 5.2 Hz, 2H), 7.87 (d, *J* = 7.9 Hz, 1H), 7.84–7.77 (m, 2H), 7.30–7.22 (m, 2H), 2.67 (s, 3H). ^13^C NMR (101 MHz, acetone-*d*_6_) *δ* 161.90, 159.27, 153.32, 153.27, 153.21, 153.16, 150.86, 150.81, 150.75, 150.70, 150.43, 150.39, 150.31, 150.27, 150.19, 150.15, 139.57, 139.42, 139.27, 137.12, 136.97, 136.82, 135.18, 134.13, 132.02, 131.70, 131.38, 130.99, 130.63, 129.67, 127.07, 127.04, 127.00, 126.97, 126.94, 126.90, 126.86, 126.55, 123.85, 108.12, 108.06, 107.95, 107.89, 21.45. HRMS (QFT-ESI) calcd for C_18_H_10_F_6_N_2_O [M + H]^+^ 385.0770, found: 385.0772.

*6-ethyl-3-(4-(trifluoromethyl)phenoxy)-4-(3-(trifluoromethyl)phenyl)pyridazine* (**3a**). Yellow solid; yield 60%; ^1^H NMR (400 MHz, acetone-*d*_6_) *δ* 8.17 (d, *J* = 7.6 Hz, 2H), 7.86 (s, 2H), 7.79 (d, *J* = 6.4 Hz, 3H), 7.49 (d, *J* = 8.0 Hz, 2H), 3.02 (q, *J* = 7.2 Hz, 2H), 1.37 (t, *J* = 7.6 Hz, 3H). ^13^C NMR (101 MHz, acetone-*d*_6_) *δ* 162.55, 161.32, 151.36, 151.21, 150.12, 150.09, 150.03, 150.00, 148.90, 148.78, 148.76, 148.66, 146.37, 146.24, 134.55, 133.22, 130.95, 130.63, 130.31, 129.99, 129.62, 129.02, 128.76, 126.04, 126.00, 125.96, 125.92, 125.88, 125.65, 122.89, 118.08, 117.98, 117.53, 117.34, 111.63, 111.43, 28.30, 13.09. HRMS (QFT-ESI) calcd for C_20_H_14_F_6_N_2_O [M + Na]^+^ 435.0903, found: 435.0908.

*6-ethyl-3-(3-fluorophenoxy)-4-(3-(trifluoromethyl)phenyl)pyridazine* (**3b**). Yellow solid; yield 60%; ^1^H NMR (400 MHz, acetone-*d*_6_) *δ* 8.18 (d, *J* = 7.4 Hz, 2H), 7.90–7.84 (m, 2H), 7.81 (t, *J* = 7.8 Hz, 1H), 7.52–7.44 (m, 1H), 7.18–7.10 (m, 2H), 7.07–7.01 (m, 1H), 3.02 (q, *J* = 7.6 Hz, 2H), 1.38 (t, *J* = 7.6 Hz, 3H).^13^C NMR (101 MHz, acetone-*d*_6_) *δ* 165.26, 163.51, 162.82, 162.18, 156.39, 156.28, 135.51, 134.11, 131.59, 131.49, 130.53, 129.91, 126.81, 118.10, 112.56, 112.35, 110.02, 109.78, 29.25, 14.04. HRMS (QFT-ESI) calcd for C_19_H_14_F_4_N_2_O [M + Na]^+^ 385.0934, found: 385.0939.

*3-(3,4-difluorophenoxy)-6-ethyl-4-(3-(trifluoromethyl)phenyl)pyridazine* (**3c**). Yellow solid; yield 50%; ^1^H NMR (400 MHz, DMSO-*d*_6_) *δ* 8.19–8.13 (m, 2H), 7.89 (d, *J* = 11.2 Hz, 2H), 7.79 (t, *J* = 7.6 Hz, 1H), 7.59–7.49 (m, 2H), 7.20–7.13 (m, 1H), 2.94 (q, *J* = 7.6 Hz, 2H), 1.31 (t, *J* = 7.6 Hz, 3H). ^13^C NMR (101 MHz, acetone-*d*_6_) *δ* 163.97, 161.92, 158.79, 135.24, 134.85, 134.09, 131.57, 131.25, 130.58, 130.56, 130.23, 130.18, 130.14, 126.99, 126.95, 126.86, 126.83, 122.97, 119.08, 109.18, 108.15, 107.91, 29.26, 14.00. HRMS (QFT-ESI) calcd for C_19_H_13_F_5_N_2_O [M + Na]^+^ 403.0840, found: 408.0848.

*3-(4-chloro-3-fluorophenoxy)-6-ethyl-4-(3-(trifluoromethyl)phenyl)pyridazine* (**3d**). Yellow oil; yield 50%; ^1^H NMR (400 MHz, CD_2_Cl_2_) *δ* 7.95–7.88 (m, 2H), 7.75 (d, *J* = 7.9 Hz, 1H), 7.65 (t, *J* = 7.8 Hz, 1H), 7.47–7.40 (m, 2H), 7.08 (dd, *J* = 9.8, 2.6 Hz, 1H), 7.01–6.96 (m, 1.3 Hz, 1H), 3.00 (q, *J* = 7.6 Hz, 2H), 1.37 (t, *J* = 7.6 Hz, 3H). ^13^C NMR (101 MHz, acetone-*d*_6_) *δ* 163.67, 162.06, 160.10, 157.64, 154.80, 154.70, 135.37, 134.11, 131.74, 130.54, 130.02, 129.86, 126.90, 119.48, 117.22, 117.05, 111.70, 111.46, 29.23, 13.99. HRMS (QFT-ESI) calcd for C_19_H_13_ClF_4_N_2_O [M + Na]^+^ 419.0545, found: 419.0541.

*3-(4-bromo-3-fluorophenoxy)-6-ethyl-4-(3-(trifluoromethyl)phenyl)pyridazine* (**3e**). Yellow solid; yield 50%; ^1^H NMR (400 MHz, CD_2_Cl_2_) *δ* 7.95–7.88 (m, 2H), 7.75 (d, *J* = 8.0 Hz, 1H), 7.65 (t, *J* = 7.6 Hz, 1H), 7.61–7.55 (m, 1H), 7.44 (s, 1H), 7.06 (dd, *J* = 9.4, 2.4 Hz, 1H), 6.89–6.84 (m, 1H), 3.00 (q, *J* = 7.6 Hz, 2H), 1.37 (t, *J* = 7.6 Hz, 3H). ^13^C NMR (101 MHz, acetone-*d*_6_) *δ* 163.97, 161.92, 158.79, 135.24, 134.85, 134.09, 131.57, 131.25, 130.58, 130.56, 130.23, 130.18, 130.14, 126.99, 126.95, 126.86, 126.83, 122.97, 119.08, 109.18, 108.15, 107.91, 29.26, 14.00. HRMS (QFT-ESI) calcd for C_19_H_13_BrF_4_N_2_O [M + Na]^+^ 463.0040, found: 463.0045.

*4-((6-ethyl-4-(3-(trifluoromethyl)phenyl)pyridazine-3-yl)oxy)-2-fluorobenzonitrile* (**3f**). Yellow solid; yield 60%; ^1^H NMR (400 MHz, acetone-*d*_6_) *δ* 8.15 (d, *J* = 6.0 Hz, 2H), 7.95–7.89 (m, 2H), 7.87 (d, *J* = 8.4 Hz, 1H), 7.80 (t, *J* = 8.0 Hz, 1H), 7.47 (dd, *J* = 10.4, 2.0 Hz, 1H), 7.35 (dd, *J* = 8.4, 2.0 Hz, 1H), 3.04 (q, *J* = 7.6 Hz, 2H), 1.37 (t, *J* = 7.6 Hz, 3H). ^13^C NMR (101 MHz, acetone-*d*_6_) *δ* 165.96, 164.45, 163.41, 161.68, 160.47, 160.36, 135.57, 135.03, 134.09, 133.39, 131.60, 131.31, 130.64, 130.45, 130.03, 127.05, 126.82, 126.43, 119.00, 114.32, 110.66, 110.44, 98.05, 97.99, 97.84, 97.79, 29.26, 13.99. HRMS (QFT-ESI) calcd for C_20_H_13_F_4_N_3_O [M + Na]^+^ 410.0887, found: 410.0889.

*3-(4-chloro-2-fluorophenoxy)-6-ethyl-4-(3-(trifluoromethyl)phenyl)pyridazine* (**3g**). Yellow solid; yield 55%; ^1^H NMR (400 MHz, CD_2_Cl_2_) *δ* 7.96 (d, *J* = 8.4 Hz, 2H), 7.75 (d, *J* = 7.6 Hz, 1H), 7.66 (t, *J* = 7.6 Hz, 1H), 7.43 (s, 1H), 7.28–7.19 (m, 3H), 2.98 (q, *J* = 7.6 Hz, 2H), 1.36 (t, *J* = 7.6 Hz, 3H). ^13^C NMR (101 MHz, acetone-*d*_6_) *δ* 163.97, 161.92, 158.79, 135.24, 134.85, 134.09, 131.57, 131.25, 130.58, 130.56, 130.23, 130.18, 130.14, 126.99, 126.95, 126.86, 126.83, 122.97, 119.08, 109.18, 108.15, 107.91, 29.26, 14.00. HRMS (QFT-ESI) calcd for C_19_H_13_ClF_4_N_2_O [M + Na]^+^ 419.0545, found: 419.0547.

*6-ethyl-4-(3-(trifluoromethyl)phenyl)-3-(3,4,5-trifluorophenoxy)pyridazine* (**3h**). Yellow oil; yield 50%; ^1^H NMR (400 MHz, acetone-*d*_6_) *δ* 8.19–8.12 (m, 2H), 7.89–7.77 (m, 3H), 7.31–7.22 (m, 2H), 3.01 (q, *J* = 7.6 Hz, 2H), 1.36 (t, *J* = 7.6 Hz, 3H). ^13^C NMR (101 MHz, acetone-*d*_6_) *δ* 163.80, 161.99, 153.26, 153.20, 153.15, 153.10, 150.80, 150.74, 150.69, 150.64, 150.39, 150.35, 150.27, 150.23, 150.15, 150.11, 135.25, 134.11, 130.54, 130.10, 126.96, 126.93, 126.89, 126.85, 126.81, 108.14, 107.91, 29.20, 13.95. HRMS (QFT-ESI) calcd for C_19_H_12_F_6_N_2_O [M + Na]^+^ 421.0746, found: 427.0751.

*4-((6-ethyl-4-(3-(trifluoromethyl)phenyl)pyridazine-3-yl)oxy)benzonitrile* (**3i**). Yellow solid; yield 65%; ^1^H NMR (400 MHz, acetone-*d*_6_) *δ* 8.16 (t, *J* = 4.0 Hz, 2H), 7.91–7.85 (m, 4H), 7.79 (t, *J* = 8.4 Hz, 1H), 7.52–7.45 (m, 2H), 3.02 (q, *J* = 7.6 Hz, 2H), 1.37 (t, *J* = 7.6 Hz, 3H). ^13^C NMR (101 MHz, acetone-*d*_6_) *δ* 163.99, 161.97, 158.86, 135.35, 134.91, 134.04, 130.63, 130.24, 130.20, 127.02, 126.99, 126.95, 126.91, 126.87, 123.05, 119.11, 109.23, 29.30, 14.04. HRMS (QFT-ESI) calcd for C_20_H_14_F_3_N_3_O [M + Na]^+^ 392.0981, found: 392.0988.

*6-cyclopropyl-3-(4-(trifluoromethyl)phenoxy)-4-(3-(trifluoromethyl)phenyl)pyridazine* (**4a**). Orange oil; yield 35%; ^1^H NMR (400 MHz, acetone-*d*_6_) *δ* 8.16 (d, *J* = 9.6 Hz, 2H), 7.86 (d, *J* = 8.0 Hz, 1H), 7.78 (dd, *J* = 14.8, 6.4 Hz, 4H), 7.47 (d, *J* = 8.6 Hz, 2H), 2.32 (tt, *J* = 8.0, 5.2 Hz, 1H), 1.15–1.08 (m, 4H). ^13^C NMR (101 MHz, acetone-d_6_) *δ* 162.55, 161.04, 157.46, 134.48, 133.22, 129.63, 128.99, 127.78, 126.94, 125.94, 121.65, 14.75, 9.54. HRMS (QFT-ESI) calcd for C_21_H_14_F_6_N_2_O [M + Na]^+^ 447.0903, found: 447.0908.

*6-cyclopropyl-3-(3-fluorophenoxy)-4-(3-(trifluoromethyl)phenyl)pyridazine* (**4b**). Yellow oil; yield 25%; ^1^H NMR (400 MHz, acetone-*d*_6_) *δ* 8.15 (d, *J* = 9.4 Hz, 2H), 7.85 (d, *J* = 8.0 Hz, 1H), 7.79 (t, *J* = 7.8 Hz, 1H), 7.72 (s, 1H), 7.50–7.42 (m, 1H), 7.14–7.05 (m, 2H), 7.01 (td, *J* = 8.6, 2.4 Hz, 1H), 2.36–2.25 (m, 1H), 1.15–1.05 (m, 4H). ^13^C NMR (101 MHz, acetone-*d*_6_) *δ* 162.97, 161.76, 152.13, 149.77, 149.64, 147.34, 147.21, 134.71, 131.54, 131.52, 129.69, 128.44, 128.38, 118.16, 117.98, 112.06, 111.87, 15.67, 10.75. HRMS (QFT-ESI) calcd for C_20_H_14_F_4_N_2_O [M + Na]^+^ 397.0934, found: 397.0938.

*6-cyclopropyl-3-(3,4-difluorophenoxy)-4-(3-(trifluoromethyl)phenyl)pyridazine* (**4c**). Orange oil; yield 25%; ^1^H NMR (400 MHz, CD_2_Cl_2_) *δ* 7.97–7.89 (m, 2H), 7.76 (d, *J* = 8.0 Hz, 1H), 7.66 (t, *J* = 7.6 Hz, 1H), 7.35 (s, 1H), 7.21 (dd, *J* = 18.8, 9.2 Hz, 1H), 7.15–7.05 (m, 1H), 6.98–6.90 (m, 1H), 2.23–2.15 (m, 1H), 1.16–1.10 (m, 4H). ^13^C NMR (101 MHz, acetone-*d*_6_) *δ* 151.87, 147.86, 142.45, 142.16, 142.07, 141.04, 140.98, 139.66, 139.58, 139.40, 137.24 137.19, 125.21, 124.14, 121.87, 121.59, 121.24, 120.95, 120.80, 120.59, 119.48, 116.96, 116.92, 116.89, 116.85, 108.95, 108.91, 108.89, 108.85, 108.12 107.89, 102.44, 102.31, 14.26, 9.87. HRMS (QFT-ESI) calcd for C_20_H_13_F_5_N_2_O [M + Na]^+^ 415.0840, found: 415.0844.

*3-(4-chloro-3-fluorophenoxy)-6-cyclopropyl-4-(3-(trifluoromethyl)phenyl)pyridazine* (**4d**). Yellow oil; yield 30%; ^1^H NMR (400 MHz, CD_3_CN) *δ* 8.17–8.09 (m, 2H), 7.91 (d, *J* = 8.0 Hz, 1H), 7.81 (t, *J* = 7.6 Hz, 1H), 7.65–7.57 (m, 2H), 7.26 (dd, *J* = 10.2, 2.4 Hz, 1H), 7.15–7.10 (m, 1H), 2.40–2.31 (m, 1H), 1.24–1.17 (m, 4H). ^13^C NMR (101 MHz, acetone-*d*_6_) *δ* 162.50, 161.00, 159.18, 156.72, 154.04, 153.95, 134.44, 133.22, 130.82, 129.61, 128.79, 127.75, 118.46, 118.43, 110.64, 110.40, 14.73, 9.51. HRMS (QFT-ESI) calcd for C_20_H_13_ClF_4_N_2_O [M + Na]^+^ 431.0545, found: 431.0545.

*3-(4-bromo-3-fluorophenoxy)-6-cyclopropyl-4-(3-(trifluoromethyl)phenyl)pyridazine* (**4e**). Orange oil; yield 25%; ^1^H NMR (400 MHz, acetone-*d*_6_) *δ* 8.13 (d, *J* = 8.4 Hz, 2H), 7.85 (d, *J* = 8.0 Hz, 1H), 7.78 (t, *J* = 7.6 Hz, 1H), 7.74–7.67 (m, 2H), 7.31 (dd, *J* = 10.0, 2.4 Hz, 1H), 7.12–7.07 (m, 1H), 2.14–2.05 (m, 1H), 1.17–1.06 (m, 4H). ^13^C NMR (101 MHz, acetone-*d*_6_) *δ* 162.60, 160.96, 160.28, 157.83, 154.81, 154.71, 134.37, 133.71, 133.22, 130.93, 130.68, 130.29, 129.97, 129.63, 128.90, 127.80, 125.93, 118.83, 110.51, 110.26, 103.71, 103.50, 14.79, 9.62. HRMS (QFT-ESI) calcd for C_20_H_13_BrF_4_N_2_O [M + Na]^+^ 475.0040, found: 475.0046.

*4-((6-cyclopropyl-4-(3-(trifluoromethyl)phenyl)pyridazine-3-yl)oxy)-2-fluorobenzonitrile* (**4f**). Yellow oil; yield 20%; ^1^H NMR (400 MHz, acetone-*d*_6_) *δ* 8.12 (d, *J* = 8.4 Hz, 2H), 7.88 (dd, *J* = 16.8, 9.2 Hz, 2H), 7.83–7.75 (m, 2H), 7.43 (dd, *J* = 10.4, 2.0 Hz, 1H), 7.31 (dd, *J* = 8.4, 2.8 Hz, 1H), 2.34 (tt, *J* = 8.0, 5.2 Hz, 1H), 1.14 (m, *J* = 4.4, 2.4 Hz, 4H). ^13^C NMR (101 MHz, Acetone-*d*_6_) *δ* 165.07, 163.31, 162.52, 160.59, 159.75, 159.63, 134.66, 134.15, 133.20, 129.45, 129.36, 128.07, 126.15, 126.11, 126.07, 126.04, 126.01, 125.97, 125.93, 117.96, 113.38, 109.57, 109.35, 14.82, 9.75. HRMS (QFT-ESI) calcd for C_21_H_13_F_4_N_3_O [M + Na]^+^ 422.0887, found: 422.0895.

*3-(4-chloro-2-fluorophenoxy)-6-cyclopropyl-4-(3-(trifluoromethyl)phenyl)pyridazine* (**4g**). Orange oil; yield 30%; ^1^H NMR (400 MHz, CDCl_3_) *δ* 7.90 (d, *J* = 10.8 Hz, 2H), 7.74 (d, *J* = 7.6 Hz, 1H), 7.64 (t, *J* = 7.6 Hz, 1H), 7.36 (s, 1H), 7.29–7.25 (m, 1H), 7.13 (t, *J* = 8.8 Hz, 1H), 7.09–7.03 (m, 1H), 2.24–2.14 (m, 1H), 1.23–1.08 (m, 4H). ^13^C NMR (101 MHz, acetone-*d*_6_) *δ* 164.03, 161.89, 158.88, 135.19, 134.76, 134.13, 131.57, 131.25, 130.42, 130.38, 130.27, 130.16, 130.14, 127.03, 126.95, 126.88, 126.79, 123.12, 119.13, 109.46, 108.15, 107.98, 14.77, 9.59. HRMS (QFT-ESI) calcd for C_20_H_13_ClF_4_N_2_O [M + Na]^+^ 431.0545, found: 431.00548.

*6-cyclopropyl-4-(3-(trifluoromethyl)phenyl)-3-(3,4,5-trifluorophenoxy)pyridazine* (**4h**). Yellow oil; yield 30%; ^1^H NMR (400 MHz, acetone-*d*_6_) *δ* 8.15 (t, *J* = 10.0 Hz, 2H), 7.86 (d, *J* = 8.8 Hz, 1H), 7.79 (t, *J* = 7.6 Hz, 1H), 7.73 (d, *J* = 6.0 Hz, 1H), 7.39 (s, 1H), 7.27–7.20 (m, 1H), 2.36 –2.26 (m, 1H), 1.14–1.07 (m, 4H). ^13^C NMR (101 MHz, acetone-d_6_) *δ* 161.78, 158.97, 153.29, 153.24, 153.18, 153.16, 150.87, 150.83, 150.77, 150.70, 150.43, 150.38, 150.36, 150.26, 150.19, 150.14, 139.57, 139.40, 139.27, 137.12, 136.96, 136.82, 135.17, 134.12, 132.04, 131.71, 131.37, 130.99, 130.66, 129.67, 127.07, 127.08, 127.99, 126.96, 126.92, 126.90, 126.86, 126.55, 123.85, 108.12, 108.06, 107.95, 107.89, 15.71, 10.46. HRMS (QFT-ESI) calcd for C_20_H_12_F_6_N_2_O [M + Na]^+^ 433.0746, found: 433.0752.

*4-((6-cyclopropyl-4-(3-(trifluoromethyl)phenyl)pyridazine-3-yl)oxy)benzonitrile* (**4i**). Orange oil; yield 30%; ^1^H NMR (400 MHz, acetone-*d*_6_) *δ* 8.15 (dd, *J* = 6.0, 5.2 Hz, 2H), 7.88–7.81 (m, 3H), 7.82–7.75 (m, 2H), 7.48–7.43 (m, 2H), 2.24–2.15 (m, 1H), 1.17–1.08 (m, 4H). ^13^C NMR (101 MHz, acetone-*d*_6_) *δ* 162.85, 160.84, 158.03, 134.33, 133.96, 133.20, 130.95, 130.63, 130.31, 129.99, 129.67, 129.18, 127.90, 121.89, 118.20, 108.15, 14.83, 9.69. HRMS (QFT-ESI) calcd for C_21_H_14_F_3_N_3_O [M + Na]^+^ 404.0981, found: 404.0988.

*6-isopropyl-3-(4-(trifluoromethyl)phenoxy)-4-(3-(trifluoromethyl)phenyl)pyridazine* (**5a**). Yellow solid; yield 40%; ^1^H NMR (400 MHz, CD_2_Cl_2_) *δ* 7.89–7.84 (m, 2H), 7.68 (d, *J* = 8.0 Hz, 1H), 7.59 (dd, *J* = 14.8, 8.0 Hz, 3H), 7.38 (s, 1H), 7.25 (d, *J* = 8.4 Hz, 2H), 3.29–3.16 (m, 1H), 1.31 (d, *J* = 7.2 Hz, 6H). ^13^C NMR (101 MHz, acetone-*d*_6_) *δ* 167.26, 162.24, 158.23, 135.53, 134.18, 131.64, 131.32, 130.57, 130.26, 128.96, 127.92, 127.88, 127.85, 127.81, 127.01, 126.97, 126.93, 126.90, 122.80, 35.16, 22.69. HRMS (QFT-ESI) calcd for C_21_H_16_F_6_N_2_O [M + Na]^+^ 449.1059, found: 449.1066.

*3-(3-fluorophenoxy)-6-isopropyl-4-(3-(trifluoromethyl)phenyl)pyridazine* (**5b**). Yellow oil; yield 60%; ^1^H NMR (400 MHz, CD_2_Cl_2_) *δ* 7.90–7.83 (m, 2H), 7.67 (d, *J* = 8.0 Hz, 1H), 7.57 (t, *J* = 7.6 Hz, 1H), 7.36 (s, 1H), 7.33–7.26 (m, 1H), 6.95–6.83 (m, 3H), 3.27–3.17 (m, 1H), 1.30 (d, *J* = 6.6 Hz, 6H). ^13^C NMR (101 MHz, acetone-*d*_6_) *δ* 166.10, 164.36, 161.93, 161.37, 155.46, 155.35, 134.69, 133.25, 130.97, 130.67, 130.58, 130.33, 129.61, 129.15, 127.90, 117.23, 111.69, 111.48, 109.20, 108.96, 34.21, 21.79. HRMS (QFT-ESI) calcd for C_20_H_16_F_4_N_2_O [M + Na]^+^ 399.1091, found: 399.1095.

*3-(3,4-difluorophenoxy)-6-isopropyl-4-(3-(trifluoromethyl)phenyl)pyridazine* (**5c**). Yellow solid; yield 60%; ^1^H NMR (400 MHz, acetone-*d*_6_) *δ* 8.17 (d, *J* = 7.6 Hz, 2H), 7.89–7.76 (m, 3H), 7.45–7.35 (m, 2H), 7.17–7.11 (m, 1H), 3.37–3.27 (m, 1H), 1.38 (d, *J* = 7.2 Hz, 6H). ^13^C NMR (101 MHz, acetone-*d*_6_) *δ* 180.47, 174.53, 169.50, 167.01, 153.83, 153.71, 148.08, 147.68, 144.60, 144.51, 144.20, 144.11, 143.89, 142.97, 142.27, 140.44, 140.19, 139.90, 139.68, 131.73, 131.52, 48.03, 36.33. HRMS (QFT-ESI) calcd for C_20_H_15_F_5_N_2_O [M + Na]^+^ 417.0997, found: 417.0999.

*3-(4-chloro-3-fluorophenoxy)-6-isopropyl-4-(3-(trifluoromethyl)phenyl)pyridazine* (**5d**). Yellow oil; yield 45%; ^1^H NMR (400 MHz, CD_2_Cl_2_) *δ* 7.88–7.81 (m, 2H), 7.68 (d, *J* = 7.6 Hz, 1H), 7.58 (t, *J* = 7.6 Hz, 1H), 7.39–7.33 (m, 2H), 7.01 (dd, *J* = 9.6, 2.4 Hz, 1H), 6.93–6.89 (m, 1H), 3.28–3.16 (m, 1H), 1.30 (d, *J* = 6.8 Hz, 6H). ^13^C NMR (101 MHz, acetone-*d*_6_) *δ* 167.17, 162.16, 160.11, 157.65, 154.75, 154.65, 135.43, 134.15, 131.74, 130.53, 130.03, 128.92, 126.96, 126.92, 126.90, 126.86, 119.56, 117.31, 117.13, 111.77, 111.54, 35.11, 22.67. HRMS (QFT-ESI) calcd for C_20_H_15_ClF_4_N_2_O [M + Na]^+^ 433.0701, found: 433.0709.

*3-(4-bromo-3-fluorophenoxy)-6-isopropyl-4-(3-(trifluoromethyl)phenyl)pyridazine* (**5e**). Yellow oil; yield 55%; ^1^H NMR (400 MHz, acetone-*d*_6_) *δ* 8.16 (d, *J* = 8.0 Hz, 2H), 7.88–7.84 (m, 2H), 7.79 (t, *J* = 7.6 Hz, 1H), 7.75–7.70 (m, 1H), 7.35 (dd, *J* = 9.6, 2.4 Hz, 1H), 7.13–7.16 (m, 1H), 3.38–3.27 (m, 1H), 1.38 (d, *J* = 6.8 Hz, 6H). ^13^C NMR (101 MHz, acetone-*d*_6_) *δ* 167.17, 162.09, 161.18, 158.73, 155.53, 155.43, 135.40, 134.60, 134.13, 131.53, 131.22, 130.51, 130.04, 128.91, 119.97, 111.64, 111.39, 104.77, 104.57, 35.09, 22.67. HRMS (QFT-ESI) calcd for C_20_H_15_BrF_4_N_2_O [M + Na]^+^ 477.0197, found: 477.0199.

*2-fluoro-4-((6-isopropyl-4-(3-(trifluoromethyl)phenyl)pyridazine-3-yl)oxy)benzonitrile* (**5f**). Yellow oil, yield 50%; ^1^H NMR (400 MHz, CD_2_Cl_2_) *δ* 7.82 (s, 1H), 7.79 (d, *J* = 7.6 Hz, 1H), 7.68 (d, *J* = 8.0 Hz, 1H), 7.61 = 7.57 (m, 2H), 7.40 (s, 1H), 7.09–7.02 (m, 2H), 3.31–3.20 (m, 1H), 1.31 (d, *J* = 7.2 Hz, 6H). ^13^C NMR (101 MHz, acetone-*d*_6_) *δ* 166.95, 165.07, 162.52, 160.87, 159.57, 159.46, 134.67, 134.26, 133.24, 130.99, 130.68, 130.36, 130.04, 129.69, 128.35, 125.87, 118.24, 113.39, 109.87, 109.64, 97.15, 96.99, 34.23, 21.70. HRMS (QFT-ESI) calcd for C_21_H_15_F_4_N_3_O [M + Na]^+^ 424.1043, found: 424.1048.

*3-(4-chloro-2-fluorophenoxy)-6-isopropyl-4-(3-trifluoromethyl)phenyl)pyridazine* (**5g**). Yellow solid; yield 45%; ^1^H NMR (400 MHz, CDCl_3_) *δ* 7.99 (d, *J* = 7.6 Hz, 2H), 7.77 (d, *J* = 7.6 Hz, 1H), 7.67 (t, *J* = 7.6 Hz, 1H), 7.45 (s, 1H), 7.31–7.15 (m, 3H), 3.35 –3.25 (m, 1H), 1.42 (d, *J* = 6.8 Hz, 6H).^13^C NMR (101 MHz, acetone-*d*_6_) *δ* 166.99, 162.36, 152.31, 152.17, 151.02, 151.00, 150.94, 150.91, 149.86, 149.76, 149.72, 149.63, 147.34, 147.22, 135.57, 134.20, 130.54, 129.86, 128.85, 127.03, 127.00, 126.96, 126.92, 126.89, 126.85, 126.82, 119.08, 119.04, 119.02, 118.98, 118.47, 118.28, 112.64, 112.44, 35.12, 22.68. HRMS (QFT-ESI) calcd for C_20_H_15_ClF_4_N_2_O [M + H]^+^ 410.0882, found: 410.0885.

*6-isopropyl-4-(3-(trifluoromethyl)phenyl)-3-(3,4,5-trifluorophenoxy)pyridazine* (**5h**). Yellow solid; yield 50%; ^1^H NMR (400 MHz, CD_2_Cl_2_) *δ* 7.84–7.77 (m, 2H), 7.67 (d, *J* = 7.6 Hz, 1H), 7.57 (t, *J* = 7.6 Hz, 1H), 7.35 (s, 1H), 6.83 (dd, *J* = 8.0, 6.0 Hz, 2H), 3.29–3.16 (m, 1H), 1.28 (d, *J* = 6.8 Hz, 6H). ^13^C NMR (101 MHz, acetone-*d*_6_) *δ* 167.29, 162.32, 154.64, 154.61, 154.52, 154.49, 154.40, 154.37, 153.44, 153.38, 153.33, 153.17, 151.00, 150.94, 150.90, 150.86, 150.81, 150.76, 150.70, 150.05, 150.01, 149.93, 149.89, 149.81, 149.77, 134.97, 134.16, 130.52, 129.69, 127.16, 127.12, 127.09, 127.05, 127.02, 126.99, 126.95, 126.91, 108.23, 108.17, 108.06, 108.00, 100.94, 100.88, 100.77, 100.71, 34.87, 22.47. HRMS (QFT-ESI) calcd for C_20_H_14_F_6_N_2_O [M + Na]^+^ 435.0903, found: 433.0709.

*4-((6-isopropyl-4-(3-(trifluoromethyl)phenyl)pyridazine-3-yl)oxy)benzonitrile* (**5i**). Yellow oil, yield 65%; ^1^H NMR (400 MHz, acetone-*d*_6_) *δ* 8.16 (d, *J* = 8.4 Hz, 2H), 7.92–7.82 (m, 4H), 7.79 (dd, *J* = 9.6, 5.6 Hz, 1H), 7.52–7.46 (m, 2H), 3.35–3.33 (m, 1H), 1.39 (d, *J* = 6.8 Hz, 6H). ^13^C NMR (101 MHz, acetone-*d*_6_) *δ* 163.98, 163.83, 162.01, 161.92, 158.80, 135.28, 134.86, 134.09, 130.59, 130.24, 130.18, 130.14, 126.91, 122.98, 119.09, 109.18, 108.15, 107.92, 29.32, 29.27, 29.23, 14.00. HRMS (QFT-ESI) calcd for C_21_H_16_F_3_N_3_O [M + Na]^+^ 406.1138, found: 406.1132.

### 4.2. Protein Overexpression and Purification

Plasmid DNA (pET15b-Synechococcus PDS) was transformed into *BL21(DE3)* chemically competent cells (Tsingke Biotechnology, Beijing, China). One colony in the plate was picked and cultured overnight in 3 mL of LB medium, containing 100 μg/mL ampicillin at 37 °C. After 12 h, the bacterial culture was transferred into 500 mL of 2× YT medium containing 100 μg/mL ampicillin. When the cell culture density reached 0.6 at OD_600_, the culture temperature was then adjusted to 22 °C, and isopropyl-β-D-thio-galactoside (IPTG, 1 mM final) was supplemented to induce the expression of PDS proteins [21]. After 16 h of cell culture, the cells were harvested by centrifugation at 13,000 rpm at 4 °C. The harvested cell pellet was suspended in 20 mL lysis buffer (100 mM phosphate buffer, 500 mM NaCl, 5 mM dithiothreitol, pH 7.4), which contained one piece of a protease inhibitor cocktail (Roche complete, EDTA-free) and l mg/mL lysozyme (Sigma, Darmstadt, Germany). The cell suspension was placed on ice for 15 min and lysed by sonication (on 1 s and off 9 s, 15 min). After sonication, the lysate was centrifuged at 13,000 rpm at 4 °C for 30 min to collect the supernatant, which was mixed with Ni–NTA agarose (Qiagen), and incubated by slow shaking at 4 °C for 2 h. The mixture of proteins and the Ni–NTA agarose were treated with the wash buffer (100 mM phosphate buffer, 500 mM NaCl, 50 mM imidazole, pH 7.4) 5 times, and the PDS protein was finally collected with the elution buffer (100 mM phosphate buffer, 500 mM NaCl, 250 mM imidazole). All the collected proteins in different fractions were combined, and concentrated with an Ultra-15 centrifugal filter unit with a 10 kDa cutoff (Millipore). The concentrated proteins were then treated with a desalting column (7K MWCO Zeba Spin Desalting Columns, Thermo Scientific, Waltham, MA, USA) to remove the excessive imidazole. The final PDS protein was stored at −80 °C for the subsequent binding assay.

### 4.3. Surface Plasmon Resonance Assay

The interactions between the obtained proteins from purification and compounds were analyzed by an SPR assay, using the Biacore 8K (Cytiva, Washington, USA) system at 25 °C [22]. The *Synechococcus* PDS protein was immobilized on a CM5 sensor chip, using the amine-coupling method. The protein immobilization level was ∼8000 RU. For the binding studies, the test compounds with different concentrations were injected at a flow rate of 30 μL/min in a running buffer of PBS-P (10 mM phosphate buffer with 2.7 mM KCl, 137 mM NaCl, and 0.05% Surfactant P20) containing 5% DMSO for the interaction measurements. Both the association time and the dissociation time were 60 s. The equilibrium dissociation constant, *K*_D_, values were calculated via the Biacore 8K evaluation software.

### 4.4. Herbicidal Activity Assay

The pre- and post-emergence herbicidal activity of the newly prepared compounds was evaluated against six kinds of weeds: *Setaria faberii* (SETFA), *Echinochloa crus-galli* (ECHCG), *Digitaria sanguinalis* (DIGSA), *Eclipta prostrate* (ECLPR), *Abutilon juncea* (ABUJU), and *Amaranthus retroflexus* (AMARE). Compound **1** and diflufenican were used as positive controls. The tested methods were the same as our previous reports [17,23,24,25,26]. Briefly, for the pre-emergence herbicidal activity assays, the tested compounds in solvent (containing Tween-80 + DMF) were sprayed on the surface of the soil in the cups. After 21 days, the herbicidal activity was evaluated (Table S2). For the post-emergence herbicidal activity tests, the weeds were treated with the tested compounds, at rates ranging from 150 to 700 g ai/ha at the 3–4-leaf stage. After 21 days post-treatment, the results were evaluated (Table 1).

### 4.5. Crop Selectivity

The pre- and post-emergence crop selectivity of compound **2a** and diflufenican were tested using similar methods to our previous reports [9,13,17]. Briefly, the seeds of six crops: maize, rice, wheat, rape, soybean, and cotton were sown in the cups, and treated with the solutions of the tested inhibitors at 300 g ai/ha. The results regarding crop safety were determined 24 days post-treatment (Table 2).

### 4.6. Mocelcuar Simulation Studies

The structures of the compounds **1** and **2a** were constructed by SYBYL6.9 [7]. The structure of *Synechococcus* PDS was built based on chain A of the structure of OsPDS (PDB id: 5MOG) by MODELER 9v19 [14,15], then optimized using Amber 14 [27,28]. The docking of **1** and **2a** to the catalytic site of *Synechococcus* PDS was performed using Autodock 4 [16], and the best binding mode of each molecule was selected based on the docking score [19,29]. The parameters for the molecular dynamic (MD) simulations were prepared using the tleap program, the optimization of the **2a**–*Synechococcus* PDS complex was performed using Sander, and the production of MD was performed using the pmemd module. The set of detailed parameters, the binding free energy calculation, and energy decomposition were the same as in the previous reports [25,30,31].

### 4.7. clogP Calculation

The clog*P* values of the compounds **1**–**5** and diflufenican were calculated using Chemdraw 19.1.

## 5. Conclusions

Based on the discussions in the above sections, we can draw the following conclusions:

1. Among the newly designed analogs, the compounds **2a**–**h** showed a higher herbicidal activity than the compounds **3a**–**i**, **4a**–**i**, and **5a**–**i** (**2** > **3** > **4** > **5**), suggesting that the substituents at 3-phenoxy-4-(3-trifluoromethylphenyl)pyridazine scaffold are crucial to the bioactivity.

2. The substituents at R^1^ had a considerable effect on the herbicidal activity of the target compounds. The effect of R^1^ on herbicidal activity decreased in the order of -CH_3_, -CH_2_CH_3_ > cyclopropyl group > -CH(CH_3_)_2_.

3. The synthesized 3-phenoxy-4-(3-trifluoromethylphenyl)pyridazines are PDS inhibitors, because these compounds showed the same whitening symptoms on the treated weeds as diflufenican, and could bind to the PDS in vitro.

4. Compound **2a** showed a higher post-emergence herbicidal activity than diflufenican at the rates of 300–750 g ai/ha, and a comparable pre-emergence herbicidal activity to diflufenican at 300–750 g ai/ha.

5. Wheat was selective to the compound **2a** by both pre- and post-emergence applications at 300 g ai/ha.

6. Our MD results showed that the hydrophilic interactions between the inhibitors and PDS are important for their binding affinity.

## Figures and Tables

**Table 1:** The post-emergence herbicidal activity of compounds **1**–**5** and diflufenican.

Compd	R^1^	R^2^	Dosage g ai/ha	ECHCG *^a^*	DIGSA	SETFA	ABUJU	AMARE	ECLPR	clog*P*
**2a**	-CH_3_	4-CF_3_	750	8 *^b^*	9	8	10	9	8	5.16
			600	7	9	7	10	9	8	
			300	6	8	6	6	8	7	
			150	2	6	2	1	3	3	
**2b**	-CH_3_	3-F	750	2	4	4	7	7	2	5.32
**2c**	-CH_3_	3,4-diF	750	3	7	5	10	9	5	5.44
**2d**	-CH_3_	3-F,4-Cl	750	7	8	8	10	9	7	6.08
			600	7	8	7	5	7	3	
**2e**	-CH_3_	3-F,4-Br	750	5	5	5	10	8	7	6.23
**2f**	-CH_3_	3-F,4-CN	750	6	6	6	10	8	6	5.00
**2g**	-CH_3_	2-F,4-Cl	750	5	5	5	10	8	7	5.85
**2h**	-CH_3_	3,4,5-triF	750	7	8	8	10	9	7	5.53
			600	7	8	7	5	7	3	
**3a**	-CH_2_CH_3_	4-CF_3_	750	3	8	5	7	9	5	6.70
**3b**	-CH_2_CH_3_	3-F	750	3	7	8	3	7	3	5.85
**3c**	-CH_2_CH_3_	3,4-diF	750	1	8	8	1	10	1	5.97
			600	1	8	7	1	5	1	
**3d**	-CH_2_CH_3_	3-F,4-Cl	750	5	8	7	10	8	5	6.61
			600	1	7	1	6	8	1	
**3e**	-CH_2_CH_3_	3-F,4-Br	750	1	3	3	1	7	2	6.76
**3f**	-CH_2_CH_3_	3-F,4-CN	750	1	2	4	5	7	1	5.52
**3g**	-CH_2_CH_3_	2-F,4-Cl	750	3	3	3	5	6	5	5.84
**3h**	-CH_2_CH_3_	3,4,5-triF	750	8	9	9	10	9	8	6.06
			600	8	8	7	10	9	6	
			300	5	8	6	9	8	1	
**3i**	-CH_2_CH_3_	4-CN	750	5	5	5	8	6	2	5.33
**4a**	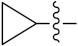	4-CF_3_	750	7	6	7	10	10	7	6.66
**4b**	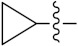	3-F	750	1	1	1	1	1	1	5.81
**4c**	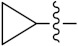	3,4-diF	750	5	8	7	10	6	1	5.94
**4d**	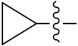	3-F,4-Cl	750	8	10	8	8	10	7	6.58
			600	3	9	4	1	7	1	
**4e**	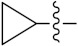	3-F,4-Br	750	1	1	1	1	1	1	6.73
**4f**	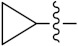	3-F,4-CN	750	1	1	1	1	1	1	5.48
**4g**	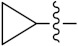	2-F,4-Cl	750	1	1	1	1	1	1	6.35
**4h**	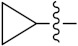	3,4,5-triF	750	7	5	4	10	7	3	6.03
**4i**	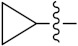	4-CN	750	2	4	3	3	7	10	5.29
**5a**	-CH(CH_3_)_2_	4-CF_3_	750	1	1	1	1	1	1	6.09
**5b**	-CH(CH_3_)_2_	3-F	750	1	1	1	1	1	1	5.35
**5c**	-CH(CH_3_)_2_	3,4-diF	750	1	1	1	1	1	1	5.42
**5d**	-CH(CH_3_)_2_	3-F,4-Cl	750	1	1	1	1	1	1	6.06
**5e**	-CH(CH_3_)_2_	3-F,4-Br	750	1	1	1	1	1	1	6.21
**5f**	-CH(CH_3_)_2_	3-F,4-CN	750	1	1	1	1	1	1	4.78
**5g**	-CH(CH_3_)_2_	2-F,4-Cl	750	1	1	1	1	1	1	5.86
**5h**	-CH(CH_3_)_2_	3,4,5-triF	750	1	1	1	1	1	1	5.49
**5i**	-CH(CH_3_)_2_	2-CN	750	1	1	1	1	1	1	6.78
**1**	-CH_3_	4-CN	750	3	7	5	6	7	5	4.80
diflufenican			750	6	6	6	10	8	6	4.23
			600	2	7	6	5	9	1	
			300	1	6	5	2	9	1	
			150	1	5	2	1	9	1	

*^a^* Abbreviation: *Echinochloa crus-galli*: ECHCG; *Digitaria sanguinalis*: DIGSA; *Setaria faberii*: SETFA; *Abutilon juncea:* ABUJU; *Eclipta prostrate*: ECLPR; *Amaranthus retroflexus*: AMARE;. *^b^* Rating scale of herbcidal activity: 10, 100%; 9, 99–90%; 8, 89–80%; 7, 79–70%; 6, 69–60%; 5, 59–50%; 4, 49–40%; 3, 39–30%; 2, 29–20%; 1, 19–0%.

**Table 2:** Post- and pre-emergence crop selectivity results of compounds **2a** and diflufenican.

Compd	Dosage g ai/ha	Maize		Rice		Wheat		Rape		Soybean		Cotton	
		Post	Pre	Post	Pre	Post	Pre	Post	Pre	Post	Pre	Post	Pre
**2a**	300	40	0	50	0	0	0	40	0	70	0	60	0
diflufenican	300	5	0	30	0	40	0	40	0	50	0	50	0

## Data Availability

Not available.

## Supplementary Materials

The supplementary Information is available online. Table S1: The structures, yields, and clogP of compounds **2**–**5**; Table S2: The pre-emergence herbicidal activity of compounds **1**–**5** and diflufenican; Table S3: Calculated binding free energies (kcal/mol) of **2a** with Synechococcus PDS; General method to synthesize intermediates **7**–**12**; The 1H NMR, 13C NMR, 19F NMR and HRMS spectrum of representative compounds.

## Sample Availability

Samples of the compounds **2**–**5** are available from the authors.
