# Supplementary material for: In Silico Structure-Guided Optimization and Molecular Simulation Studies of 3-Phenoxy-4-(3-trifluoromethylphenyl)pyridazines as Potent Phytoene Desaturase Inhibitors
Source: Molecules. 2021 Nov 19;26(22):6979. doi: 10.3390/molecules26226979 (PMC8618034; doi:10.3390/molecules26226979)
Supplement: Supplementary file 1 [file molecules-26-06979-s001.zip › molecules-1469540-supplementary.pdf]

Supporting Information for:

In silico Structure-guided Optimization and Molecular Simulation Studies of 3-Phenoxy-4-(3-Trifluoromethylphenyl)pyridazines as Potent Phytoene Desaturase Inhibitors

Lijun Yang [1,†], Dawei Wang [1,†], Dejun Ma [1], Di Zhang [1], Nuo Zhou [1], Jing Wang [2], Han Xu [1,*] and Zhen Xi [1,*]

[1] National Pesticide Engineering Research Center, Department of Chemical Biology, State Key Laboratory of Elemento-Organic Chemistry, and College of Chemistry, Nankai University, Tianjin 300071, China; younglj1997@163.com (L.Y.); wangdw@nankai.edu.cn (D.W.); madejun@nankai.edu.cn (D.M.); zhangdi022@126.com (D.Z.); zhounuo2345@163.com (N.Z.)

[2] State Key Laboratory of Natural and Biomimetic Drugs, School of Pharmaceutical Sciences, Peking University, No. 38, Xueyuan Road, Beijing 100191, China; wangjing1988@bjmu.edu.cn

* Correspondence: xuhan@nankai.edu.cn (H.X.); zhenxi@nankai.edu.cn (Z.X.)

† These authors contributed equally to this work

Contents:

Table S1. The structures, yields, and clog*P* of compounds **2**–**5**. S2

Table S2. The pre-emergence herbicidal activity of compounds **1**–**5** and diflufenican. S3

Table S3. Calculated binding free energies (kcal/mol) of **2a** with *Synechococcus* PDS. S4

1 General method S5

The ^{1}H NMR, ^{13}C NMR, ^{19}F NMR, and HRMS spectrum of representative compounds S7

Table S1. The structures, yields, and clog*P* of compounds **2-5**.

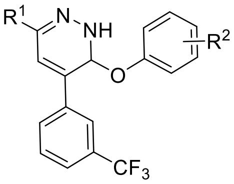

compd	R^1	R^2	yield/%	clog*P*
**2a**	$-CH_3$	$4-CF_3$	85	5.16
**2b**	$-CH_3$	3-F	60	5.32
**2c**	$-CH_3$	3,4-diF	85	5.44
**2d**	$-CH_3$	3-F,4-Cl	75	6.08
**2e**	$-CH_3$	3-F,4-Br	82	6.23
**2f**	$-CH_3$	3-F,4-CN	75	5.00
**2g**	$-CH_3$	2-F,4-Cl	78	5.85
**2h**	$-CH_3$	3,4,5-triF	65	5.53
**3a**	$-CH_2CH_3$	$4-CF_3$	60	6.70
**3b**	$-CH_2CH_3$	3-F	60	5.85
**3c**	$-CH_2CH_3$	3,4-diF	50	5.97
**3d**	$-CH_2CH_3$	3-F,4-Cl	50	6.61
**3e**	$-CH_2CH_3$	3-F,4-Br	50	6.76
**3f**	$-CH_2CH_3$	3-F,4-CN	60	5.52
**3g**	$-CH_2CH_3$	2-F,4-Cl	55	5.84
**3h**	$-CH_2CH_3$	3,4,5-triF	50	6.06
**3i**	$-CH_2CH_3$	4-CN	65	5.33
**4a**	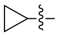	$4-CF_3$	35	6.66
**4b**	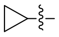	3-F	25	5.81
**4c**	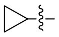	3,4-diF	25	5.94
**4d**	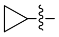	3-F,4-Cl	30	6.58
**4e**	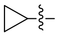	3-F,4-Br	25	6.73
**4f**	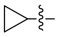	3-F,4-CN	20	5.48
**4g**	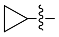	2-F,4-Cl	30	6.35
**4h**	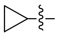	3,4,5-triF	30	6.03
**4i**	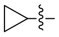	4-CN	30	5.29
**5a**	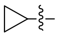	$4-CF_3$	40	6.09
**5b**	$-CH(CH_3)_2$	3-F	60	5.35
**5c**	$-CH(CH_3)_2$	3-F,4-F	60	5.42
**5d**	$-CH(CH_3)_2$	3-F,4-Cl	45	6.06
**5e**	$-CH(CH_3)_2$	3-F,4-Br	55	6.21
**5f**	$-CH(CH_3)_2$	3-F,4-CN	50	4.78
**5g**	$-CH(CH_3)_2$	2-F,4-Cl	45	5.86
**5h**	$-CH(CH_3)_2$	3,4,5-triF	50	5.49
**5i**	$-CH(CH_3)_2$	4-CN	65	6.78

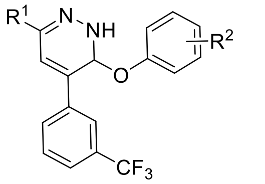

Table S2. The pre-emergence herbicidal activity of compounds **1-5** and diflufenican.

compd	R^1	R^2	dosage g ai /ha	ECHCG	DIGSA	SETFA	ABUJU	AMARE	ECLPR
**2a**	$-CH_3$	$4-CF_3$	750	8	10	9	5	10	9
			600	8	10	9	1	10	8
			300	7	10	9	1	10	7
			150	3	10	4	1	9	3
**2b**	$-CH_3$	3-F	750	1	4	1	1	3	1
**2c**	$-CH_3$	3,4-diF	750	7	8	4	1	9	5
**2d**	$-CH_3$	3-F,4-Cl	750	2	9	5	1	9	3
**2e**	$-CH_3$	3-F,4-Br	750	3	7	3	1	9	1
**2f**	$-CH_3$	3-F,4-CN	750	2	8	2	1	9	7
**2g**	$-CH_3$	2-F,4-Cl	750	1	1	2	1	8	1
**2h**	$-CH_3$	3,4,5-triF	750	3	10	9	1	9	2
			600	5	10	9	1	8	7
**3a**	$-CH_2CH_3$	$4-CF_3$	750	2	9	5	1	9	3
**3b**	$-CH_2CH_3$	3-F	750	1	7	2	1	5	1
**3c**	$-CH_2CH_3$	3,4-diF	750	1	8	8	1	10	1
			600	1	8	7	1	5	1
**3d**	$-CH_2CH_3$	3-F,4-Cl	750	1	5	2	1	1	1
**3e**	$-CH_2CH_3$	3-F,4-Br	750	1	1	1	1	1	1
**3f**	$-CH_2CH_3$	3-F,4-CN	750	1	7	2	1	1	1
**3g**	$-CH_2CH_3$	2-F,4-Cl	750	1	1	1	1	1	1
**3h**	$-CH_2CH_3$	3,4,5-triF	750	7	10	9	1	10	6
			600	7	10	8	1	10	5
			300	2	9	3	1	8	3
**3i**	$-CH_2CH_3$	4-CN	750	2	3	2	1	8	1
**4a**	▷-	$4-CF_3$	750	1	7	2	1	7	1
**4b**	▷-	3-F	750	1	1	1	1	1	1
**4c**	▷-	3,4-diF	750	1	1	1	1	1	1
**4d**	▷-	3-F,4-Cl	750	1	1	1	1	1	1
**4e**	▷-	3-F,4-Br	750	1	1	1	1	1	1
**4f**	▷-	3-F,4-CN	750	1	1	1	1	1	1
**4g**	▷-	2-F,4-Cl	750	1	1	1	1	1	1
**4h**	▷-	3,4,5-triF	750	1	8	3	1	8	1
**4i**	▷-	4-CN	750	1	4	1	1	4	2
**5a**	▷-	$4-CF_3$	750	1	1	1	1	1	1
**5b**	$-CH(CH_3)_2$	3-F	750	1	1	1	1	1	1
**5c**	$-CH(CH_3)_2$	3-F,4-F	750	1	1	1	1	1	1
**5d**	$-CH(CH_3)_2$	3-F,4-Cl	750	1	1	1	1	1	1
**5e**	$-CH(CH_3)_2$	3-F,4-Br	750	1	1	1	1	1	1
**5f**	$-CH(CH_3)_2$	3-F,4-CN	750	1	1	1	1	1	1
**5g**	$-CH(CH_3)_2$	2-F,4-Cl	750	1	1	1	1	1	1

**5h**	-$CH(CH_3)_2$	3,4,5-triF	750	1	1	1	1	1	1
**5i**	-$CH(CH_3)_2$	4-CN	750	1	1	1	1	1	1
**1**	-CH_3	4-CN	750	5	5	5	10	10	7
diflufenican			750	7	10	8	1	10	2
			600	10	10	8	1	9	2
			300	10	10	8	1	9	1
			150	10	10	7	1	9	1

[a]Abbreviation: *Echinochloa crus-galli*: ECHCG; *Digitaria sanguinalis*: DIGSA; *Setaria faberii*: SETFA; *Abutilon juncea:* ABUJU; *Eclipta prostrate*: ECLPR; *Amaranthus retroflexus*: AMARE;. [b]Rating scale of herbcidal activity: 10, 100%; 9, 99−90%; 8, 89−80%; 7, 79−70%; 6, 69−60%; 5, 59−50%; 4, 49−40%; 3, 39−30%; 2, 29−20%; 1, 19−0%.

Table S3. Calculated binding free energies (kcal/mol) of **2a** with *Synechococcus* PDS.

compd	ΔE_{ele}	ΔE_{VDW}	ΔE_{MM}	ΔG_{sol}	ΔE_{bind}	$-T\Delta S$	ΔG_{bind}[a]
**2a**	-12.95±2.28	-52.04±1.93	-64.99±2.66	35.54±2.71	-29.45 ± 2.48	10.08±2.43	-19.37±2.46

[a]$\Delta G_{bind} = \Delta E_{ele} + \Delta E_{vdw} + \Delta G\mathrm{sol} + (-T\Delta S)$.

1 General method

*1.1 General method to synthesize intermediates **7-12**.*

1.1.1 Preparation of ethyl 2-(3-(trifluoromethyl)phenyl)acetate (**7**).

H_2SO_4 (0.5 mL) was added to a solution of 2-oxo-3-(3-(trifluoromethyl)phenyl)propanoic acid **6** (20.0 g, 98 mmol) and C_2H_5OH (100 mL) at room temperature and then heated to 80 °C for 12 h. Then, the reaction cooled to 25 °C and concentrated in vacuo. The resulting residue was dissolved in CH_2Cl_2 (150 mL), and the organic layer was washed with H_2O (100 mL) and saturated Na_2CO_3 solution (100 mL), dried over magnesium sulfate and filtrated. The filtrate was concentrated to give 18.2 g yellow oil of intermediate **7**. Yield, 80%, ^1H NMR (400 MHz, $CDCl_3$) δ 7.57 – 7.42 (m, 4H), 4.17 (q, J = 7.2 Hz, 2H), 3.67 (s, 2H), 1.26 (t, J = 7.2 Hz, 3H).

1.2 Preparation of ethyl 2-bromo-2-(3-(trifluoromethyl)phenyl)acetate (**8**)

A solution of ethyl 2-(3-(trifluoromethyl)phenyl)acetate **7** (10.0 g, 32 mmol), *N*-bromosuccinimide (NBS, 6.3 g, 35.2 mmol), and azobisisobutyronitrile (AIBN, 5.2 g，3.2 mmol) was heated to reflux for 10 h with stirring. Then, the reaction solution was cooled to 25 °C. The resulting undissolved substance was removed by filtration. The filtrate was concentrated and purified by column chromatography to afford 7.1 g of orange oil **8**. Yield, 70%, ^1H NMR (400 MHz, $CDCl_3$) δ 7.81 (s, 1H), 7.77 (d, J = 7.8 Hz, 1H), 7.61 (d, J = 7.8 Hz, 1H), 7.50 (t, J = 7.8 Hz, 1H), 5.36 (s, 1H), 4.31 – 4.21 (m, 2H), 1.29 (t, J = 7.2 Hz, 3H).

1.3 The synthetic methods for intermediates **9-12** *were the same as our previous work.*

6-methyl-4-(3-(trifluoromethyl)phenyl)pyridazin-3(2H)-one (**11a**). Yellow soild, 50% yield; ^1H NMR (400 MHz, $CDCl_3$) δ 11.77 (s, 1H), 8.10 (d, J = 7.6 Hz, 1H), 8.02 (s, 1H), 7.69 (d, J = 7.6 Hz, 1H), 7.59 (t, J = 7.8 Hz, 1H), 7.32 (s, 1H), 2.42 (s, 3H).

6-ethyl-4-(3-(trifluoromethyl)phenyl)pyridazin-3(2H)-one (**11b**). Yellow soild, 50% yield; ^1H NMR (400 MHz, DMSO-d_6) δ 12.98 (s, 1H), 8.32 (s, 1H), 8.27 (d, J = 7.6 Hz, 1H), 7.96 (d, J = 7.6 Hz, 1H), 7.82 (s, 1H), 7.79(t, J = 7.6 Hz, 1H), 2.98 (q, J = 7.6 Hz, 2H), 1.19 (t, J = 7.6 Hz, 3H).

6-cyclopropyl-4-(3-(trifluoromethyl)phenyl)pyridazin-3(2H)-one (**11c**). Yellow soild, 30% yield; ^1H NMR (400 MHz, DMSO-d_6) δ 12.99 (s, 1H), 8.30 (s, 1H), 8.17 (d, J = 8.0 Hz, 1H), 7.80 (d, J = 8.0 Hz, 1H), 7.70 (t, J = 7.8 Hz, 1H), 7.61 (s, 1H), 2.01 (tt, J = 8.3, 5.0 Hz, 1H), 0.97 – 0.85 (m, 4H).

6-isopropyl-4-(3-(trifluoromethyl)phenyl)pyridazin-3(2H)-one (**11d**). Yellow soild, 35% yield; ^1H NMR (400 MHz, DMSO-d_6) δ 13.04 (s, 1H), 8.31 (s, 1H), 8.18 (d, J = 7.6 Hz, 1H), 7.79 (d, J = 10.0 Hz, 2H), 7.69 (t, J = 7.6 Hz, 1H), 3.01 – 2.93 (m, 1H), 1.22 (d, J = 6.8 Hz, 6H).

3-chloro-6-methyl-4-(3-(trifluoromethyl)phenyl)pyridazine (**12a**). White soild, 50% yield; ^1H NMR (400 MHz, $CDCl_3$) δ 7.77 (d, J = 7.6 Hz, 1H), 7.73 (s, 1H), 7.71 (d, J = 7.6 Hz, 1H), 7.65 (t, J = 7.6 Hz, 1H), 7.35 (s, 1H), 2.78 (s, 3H).

3-chloro-6-ethyl-4-(3-(trifluoromethyl)phenyl)pyridazine (**12b**). Yellow soild; 70% yield; ^1H NMR (400 MHz, DMSO) δ 8.26 (s, 1H), 8.15 (d, J = 7.9 Hz, 1H), 7.83 (d, J = 7.8 Hz, 1H), 7.78 (s, 1H), 7.72 (t, J = 7.9 Hz, 1H), 2.62 (q, J = 7.5 Hz, 2H), 1.12 (t, J = 7.5 Hz, 3H).

3-chloro-6-cyclopropyl-4-(3-(trifluoromethyl)phenyl)pyridazine (**12c**). Yellow soild; 30% yield; ^1H NMR (400 MHz, DMSO-d_6) δ 8.00 (s, 1H), 7.95 – 7.88 (m, 2H), 7.79 (t, J = 7.6 Hz, 1H), 7.74 (s, 1H), 2.34

(tt, $J = 7.6, 5.2$ Hz, 1H), 1.24 – 1.10 (m, 4H).

3-chloro-6-isopropyl-4-(3-(trifluoromethyl)phenyl)pyridazine (**12d**). Yellow oil, 70% yield; ^1H NMR (400 MHz, $CDCl_3$) δ 7.78 – 7.71 (m, 2H), 7.69 (d, $J = 8.0$ Hz, 1H), 7.64 (t, $J = 7.6$ Hz, 1H), 7.32 (s, 1H), 3.40 – 3.32 (m, 1H), 1.39 (d, $J = 7.2$ Hz, 6H).

The ^{1}H NMR, ^{13}C NMR, ^{19}F NMR, and HRMS spectrum of representative compounds.

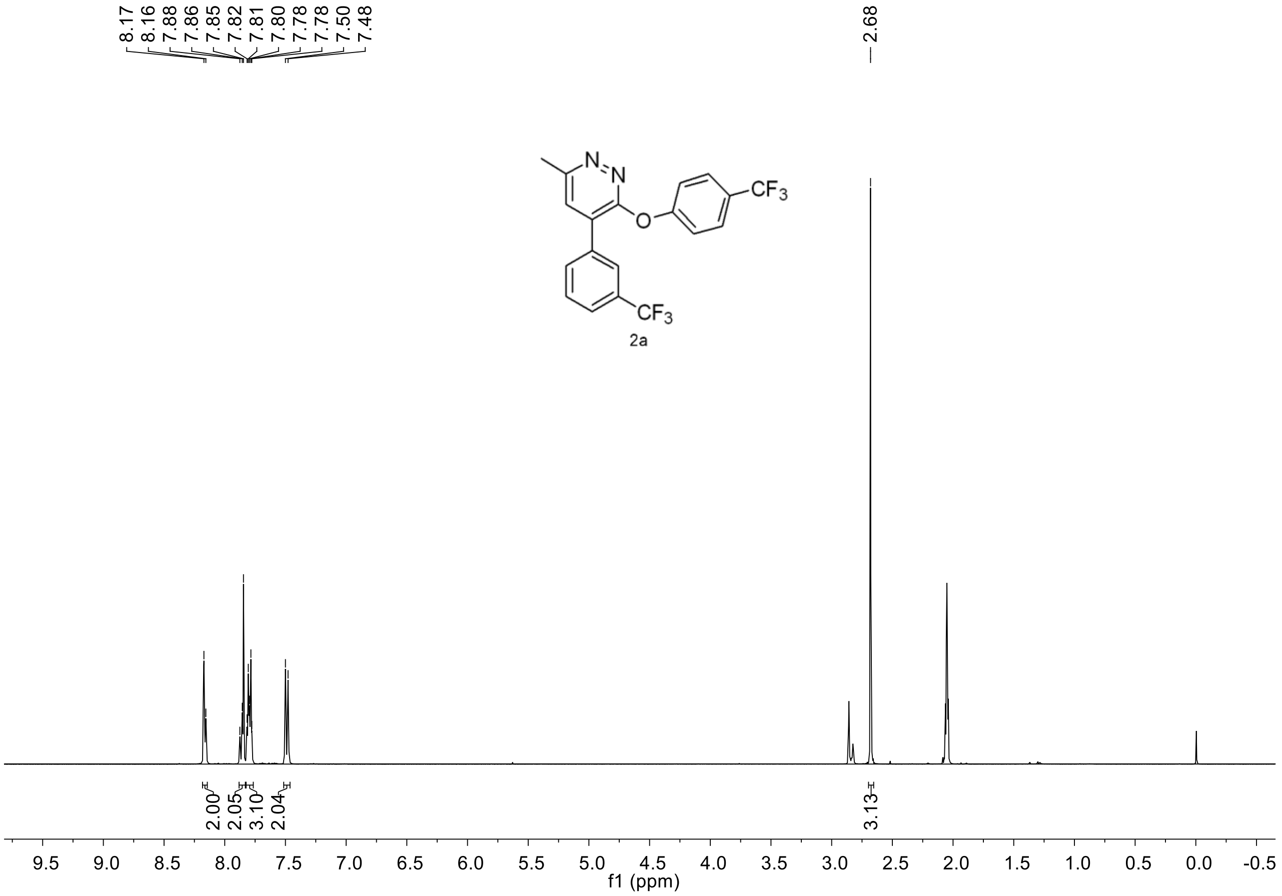

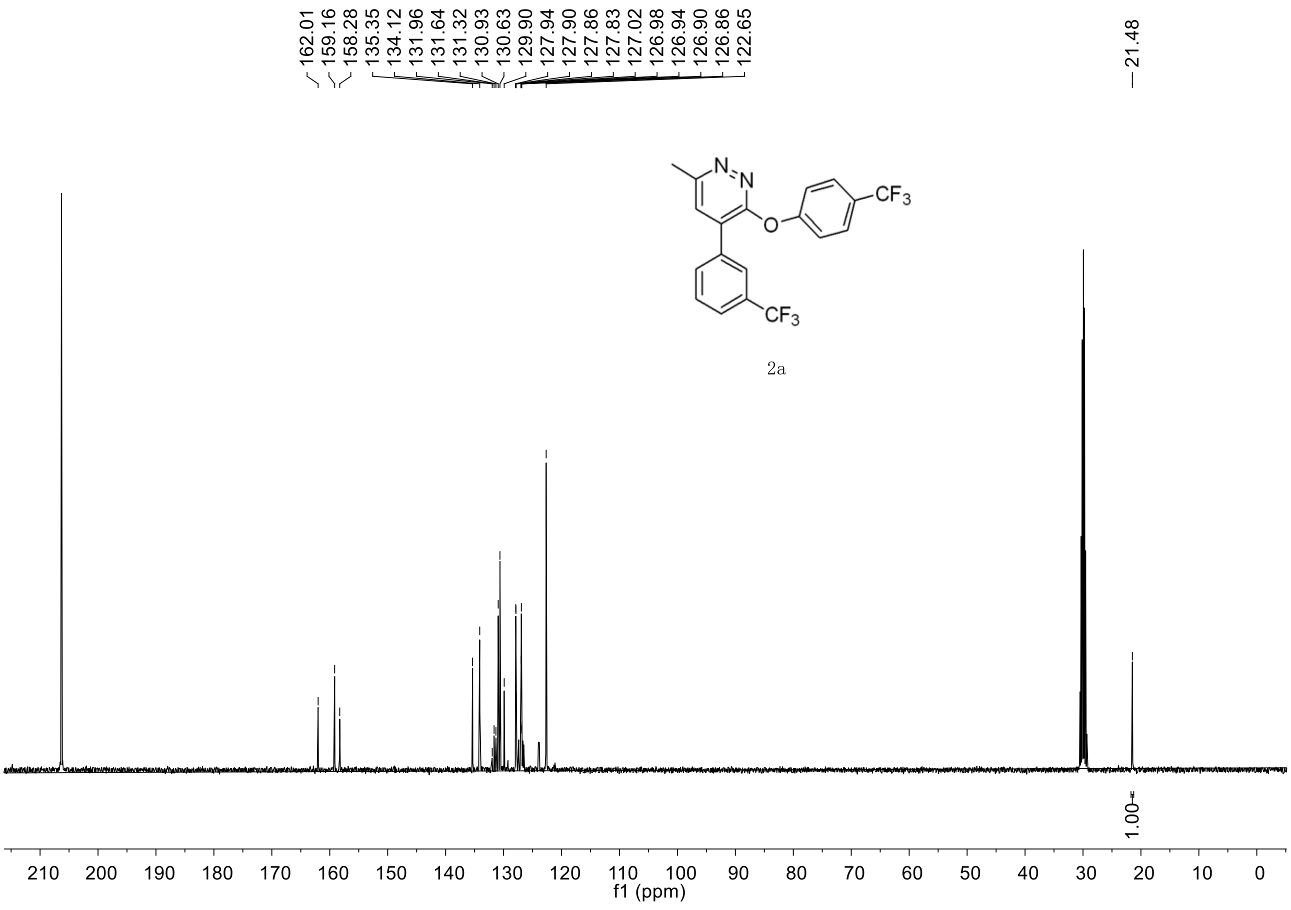

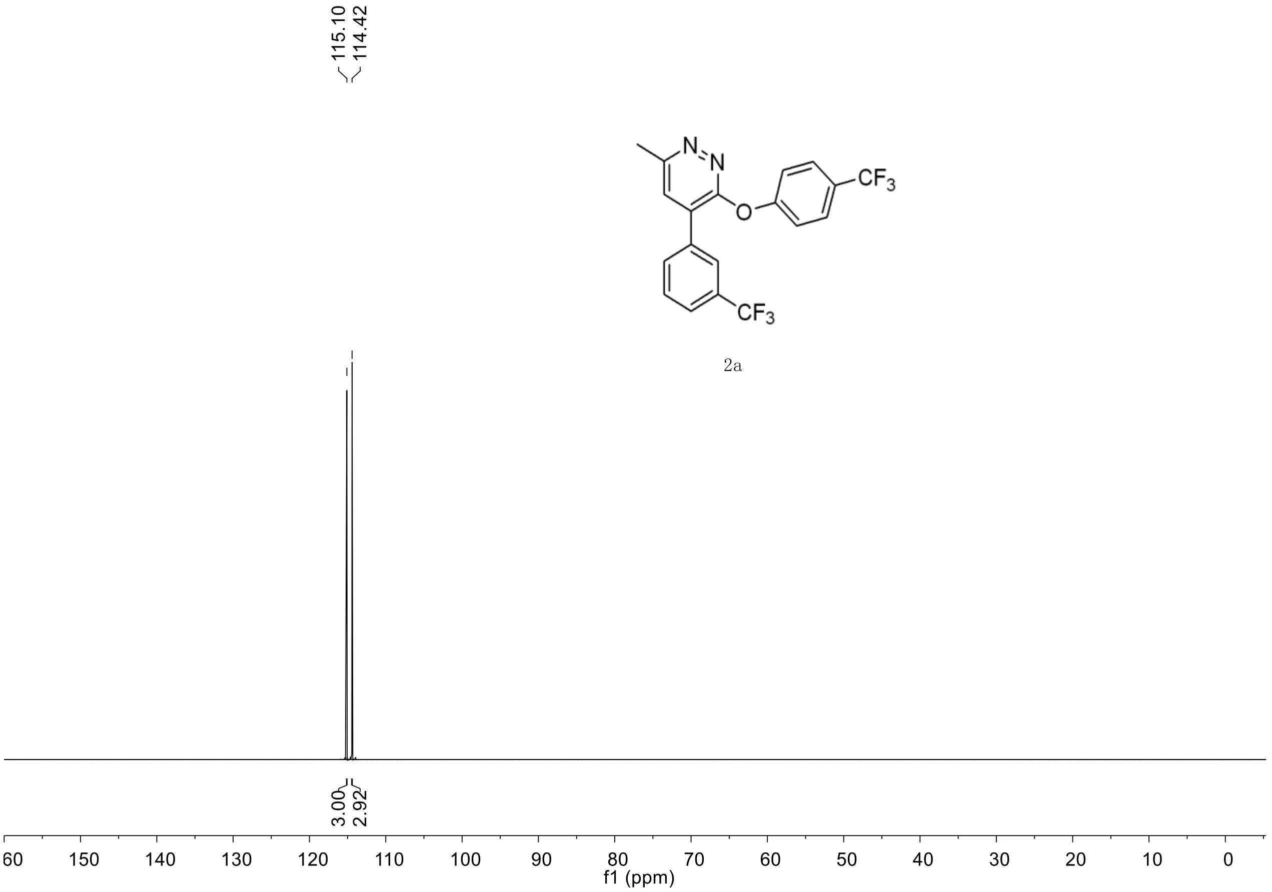

115.10
114.42
CF_3
CF_3
2a
3.00
2.92
60 150 140 130 120 110 100 90 80 70 60 50 40 30 20 10 0
f1 (ppm)

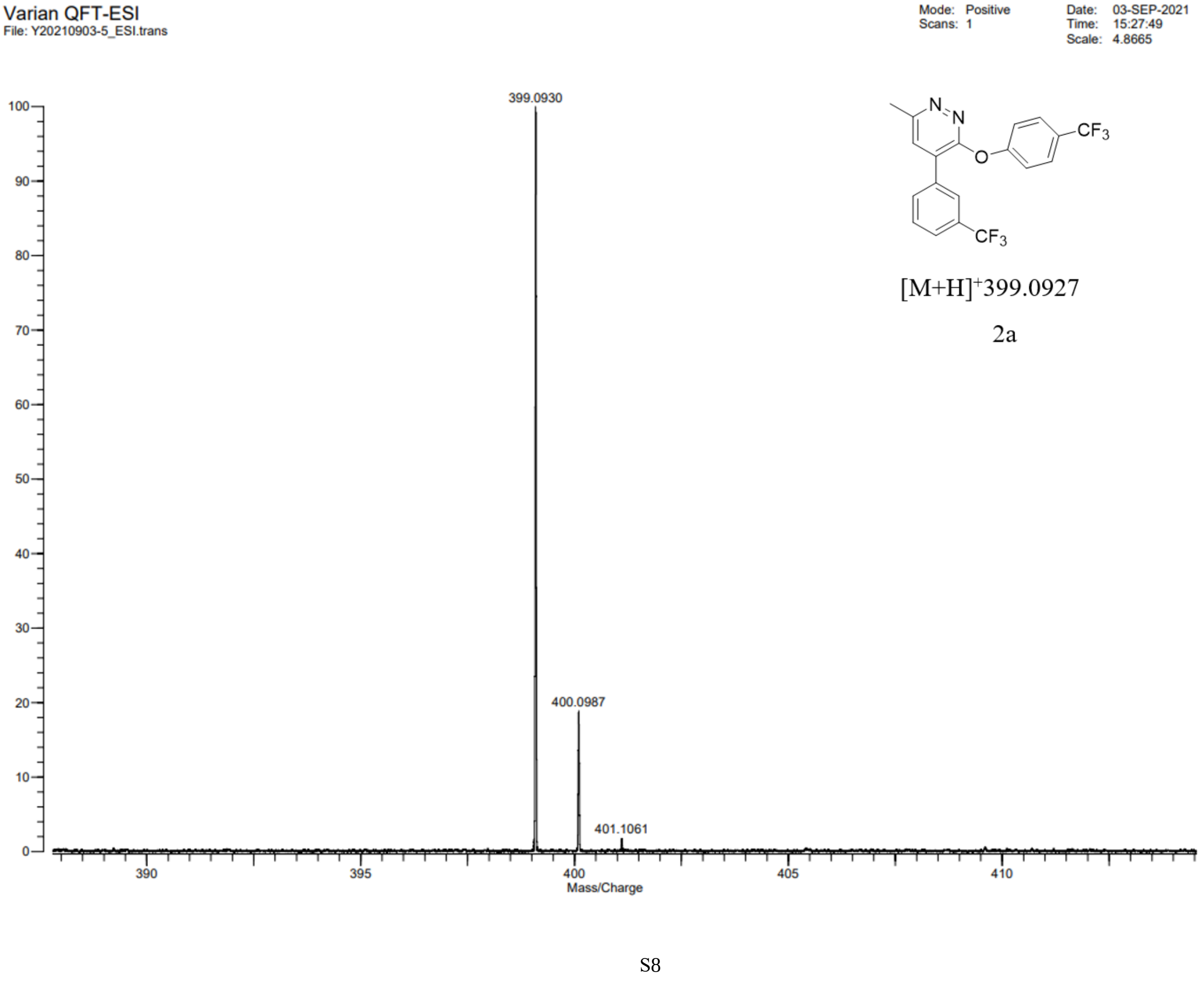

Varian QFT-ESI
File: Y20210903-5_ESI.trans
Mode: Positive
Scans: 1
Date: 03-SEP-2021
Time: 15:27:49
Scale: 4.8665
399.0930
400.0987
401.1061
Mass/Charge
$[M+H]^+$399.0927
2a

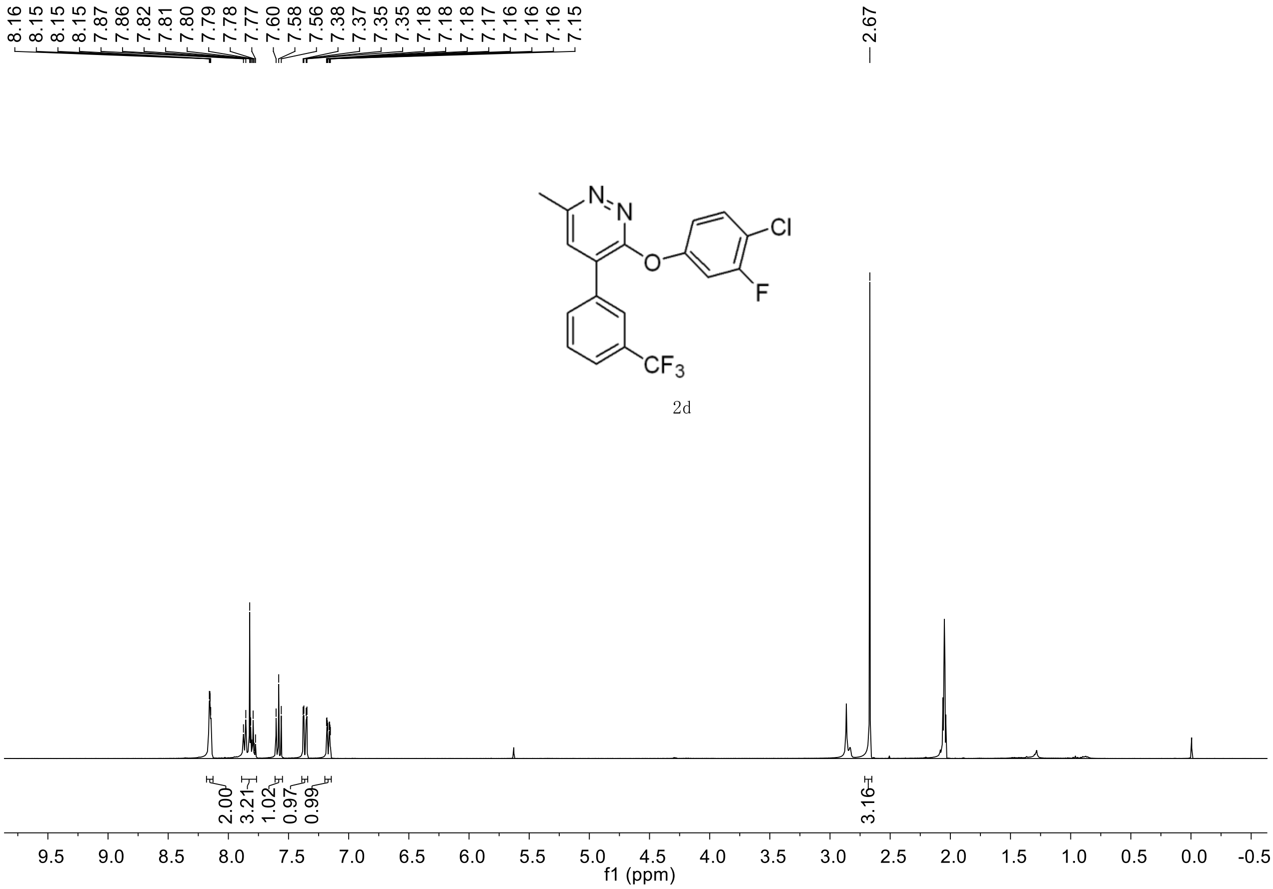
8.16 8.15 8.15 8.15 7.87 7.86 7.82 7.81 7.80 7.79 7.78 7.77 7.60 7.58 7.56 7.38 7.37 7.35 7.35 7.18 7.18 7.18 7.17 7.16 7.16 7.16 7.15
2.67
Cl
F
O
N
N
CF3
2d
2.00 3.21 1.02 0.97 0.99
3.16
9.5 9.0 8.5 8.0 7.5 7.0 6.5 6.0 5.5 5.0 4.5 4.0 3.5 3.0 2.5 2.0 1.5 1.0 0.5 0.0 -0.5
f1 (ppm)

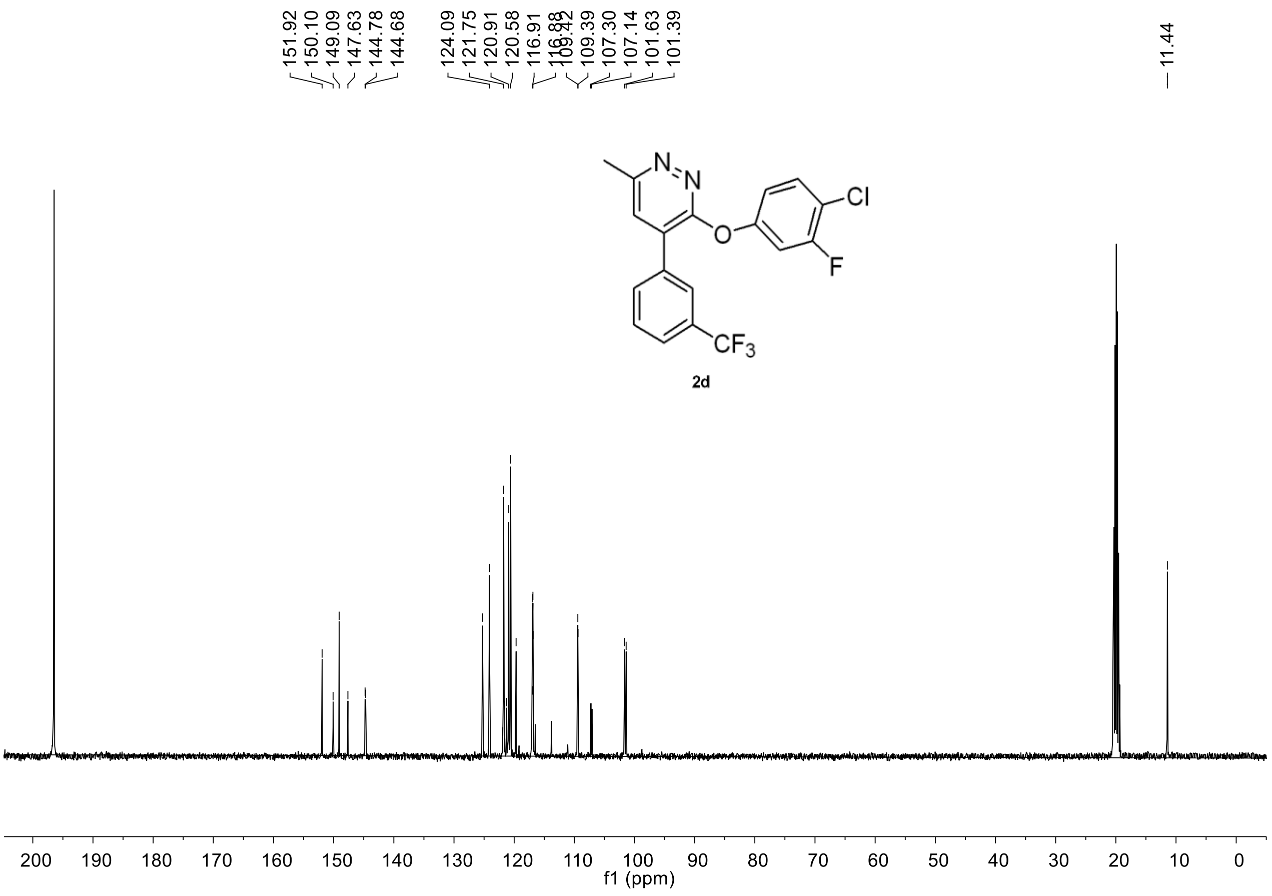
151.92 150.10 149.09 147.63 144.78 144.68
124.09 121.75 120.91 120.58 116.91 116.88 109.42 109.39 107.30 107.14 101.63 101.39
11.44
Cl
F
O
N
N
CF3
2d
200 190 180 170 160 150 140 130 120 110 100 90 80 70 60 50 40 30 20 10 0
f1 (ppm)

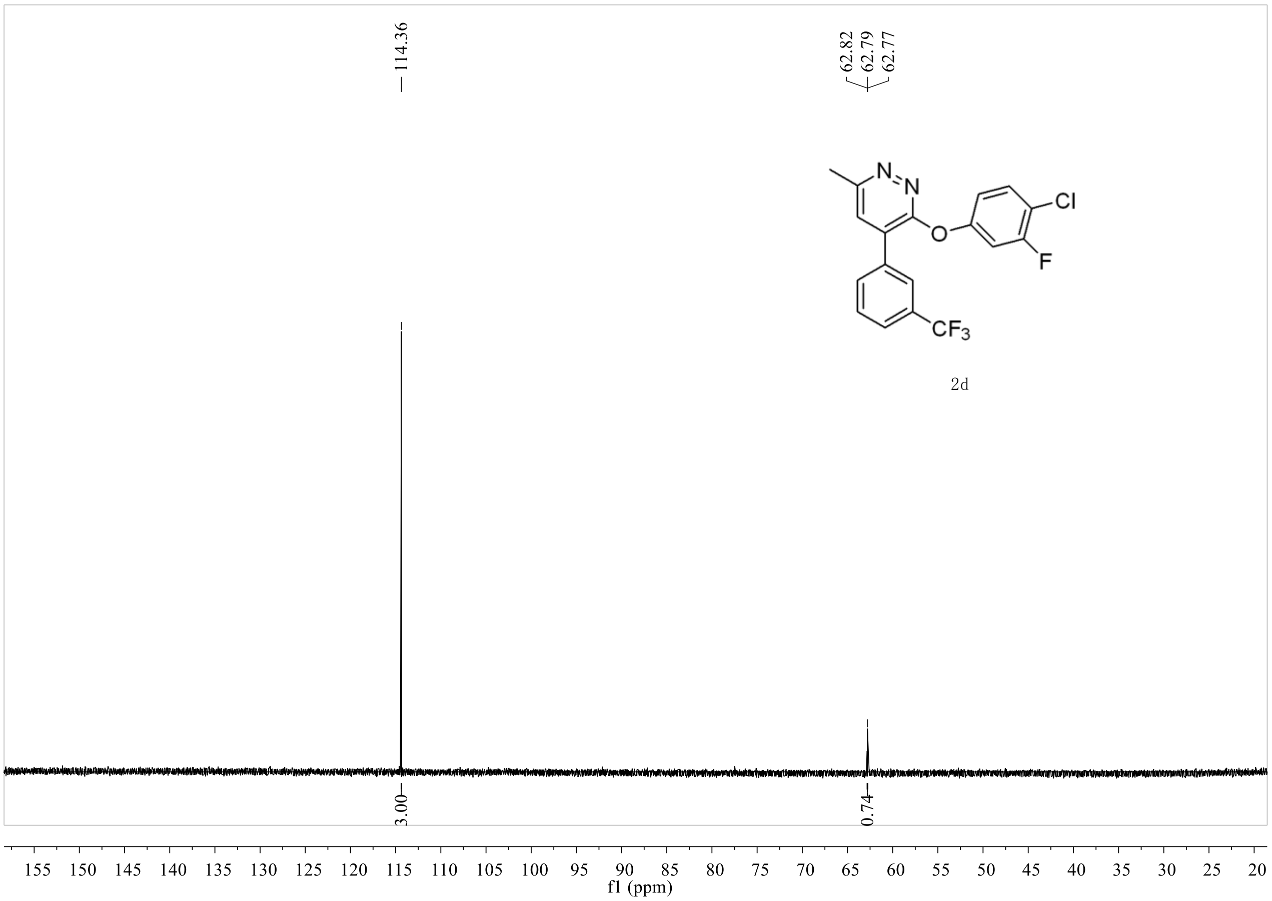

114.36
62.82
62.79
62.77
2d
3.00
0.74
155 150 145 140 135 130 125 120 115 110 105 100 95 90 85 80 75 70 65 60 55 50 45 40 35 30 25 20
f1 (ppm)

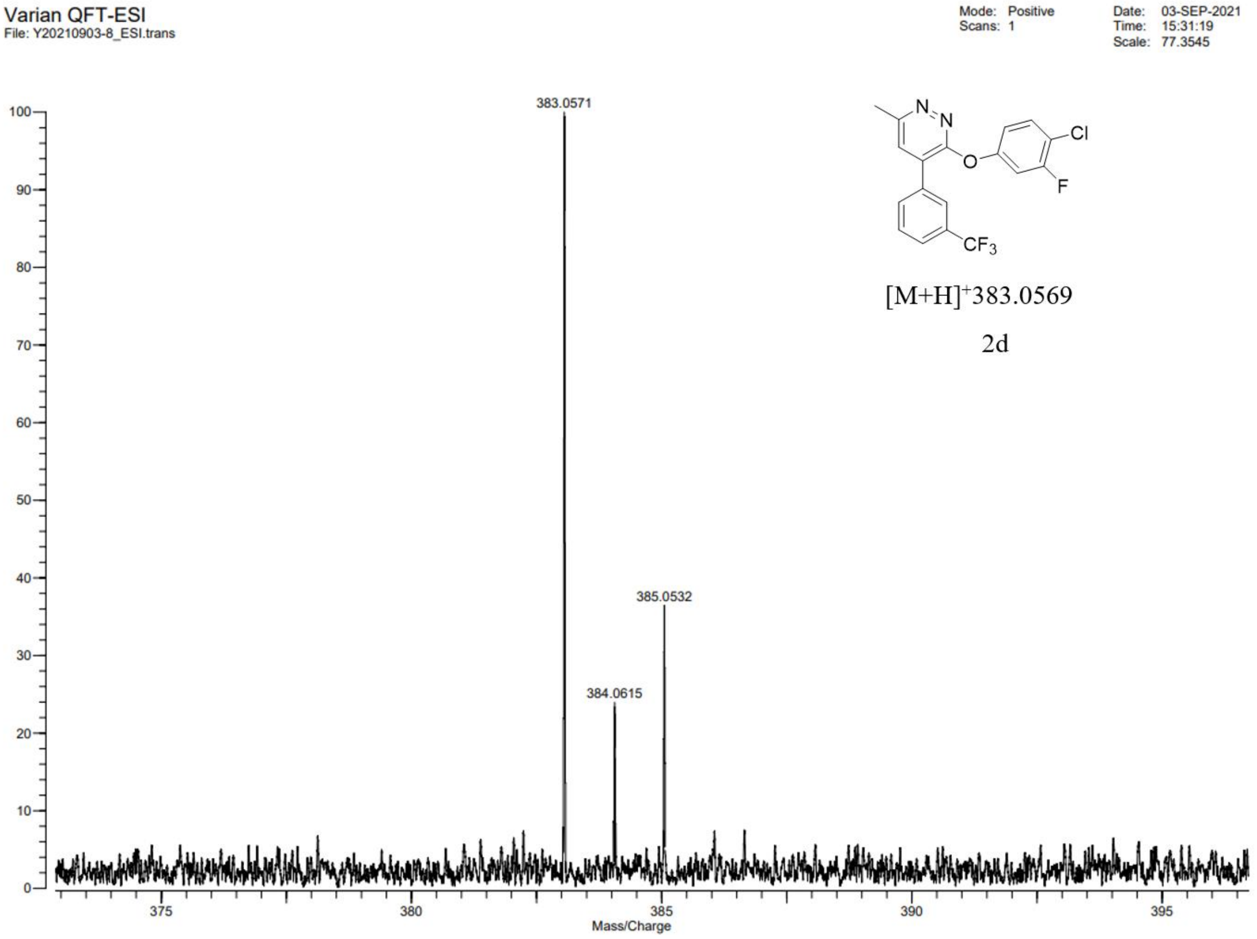

Varian QFT-ESI
File: Y20210903-8_ESI.trans
Mode: Positive
Scans: 1
Date: 03-SEP-2021
Time: 15:31:19
Scale: 77.3545
383.0571
385.0532
384.0615
[M+H]+383.0569
2d
Mass/Charge

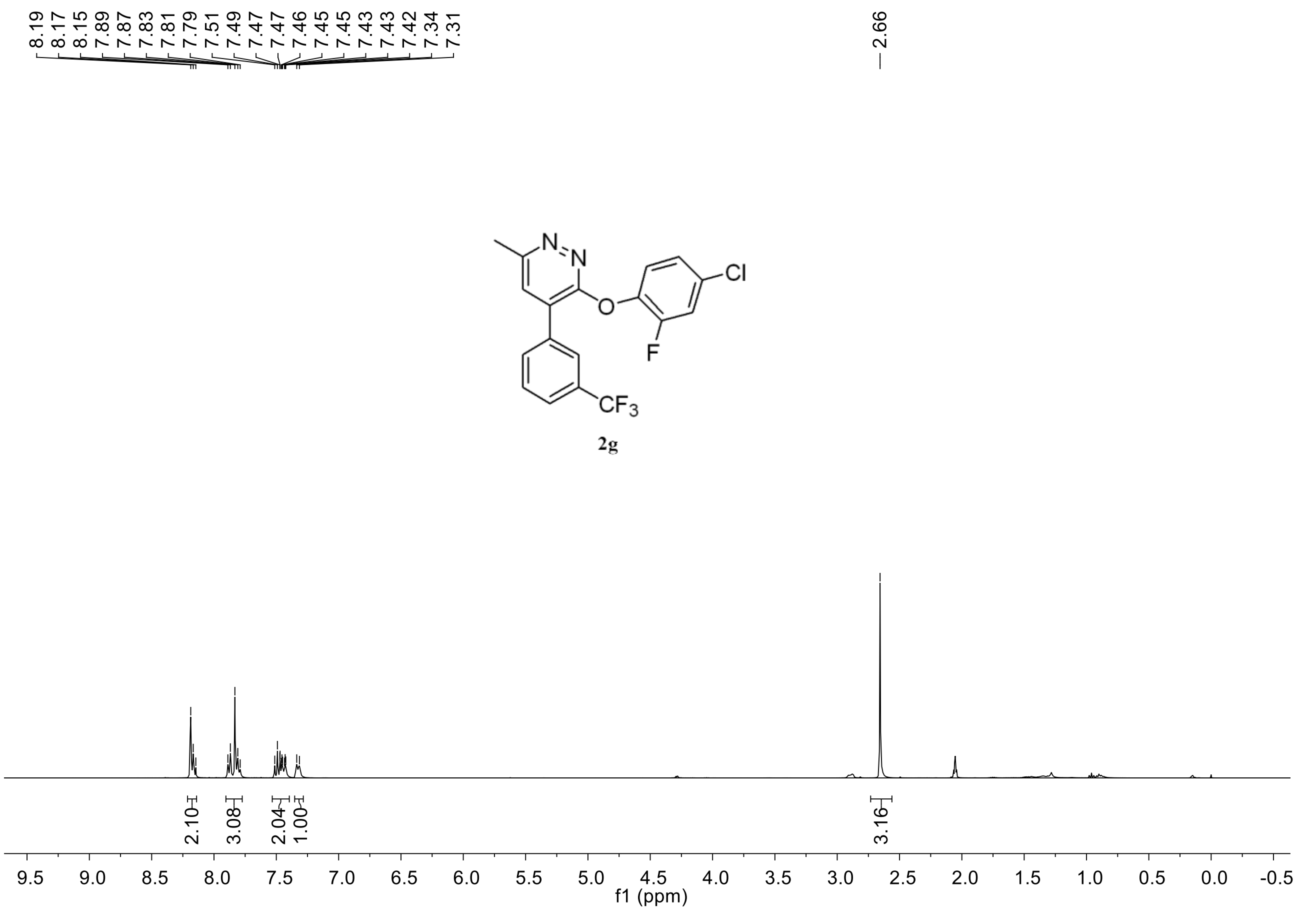
8.19
8.17
8.15
7.89
7.87
7.83
7.81
7.79
7.51
7.49
7.47
7.46
7.45
7.45
7.43
7.43
7.42
7.34
7.31
2.66
N
N
O
Cl
F
CF3
2g
2.10
3.08
2.04
1.00
3.16
9.5 9.0 8.5 8.0 7.5 7.0 6.5 6.0 5.5 5.0 4.5 4.0 3.5 3.0 2.5 2.0 1.5 1.0 0.5 0.0 -0.5
f1 (ppm)

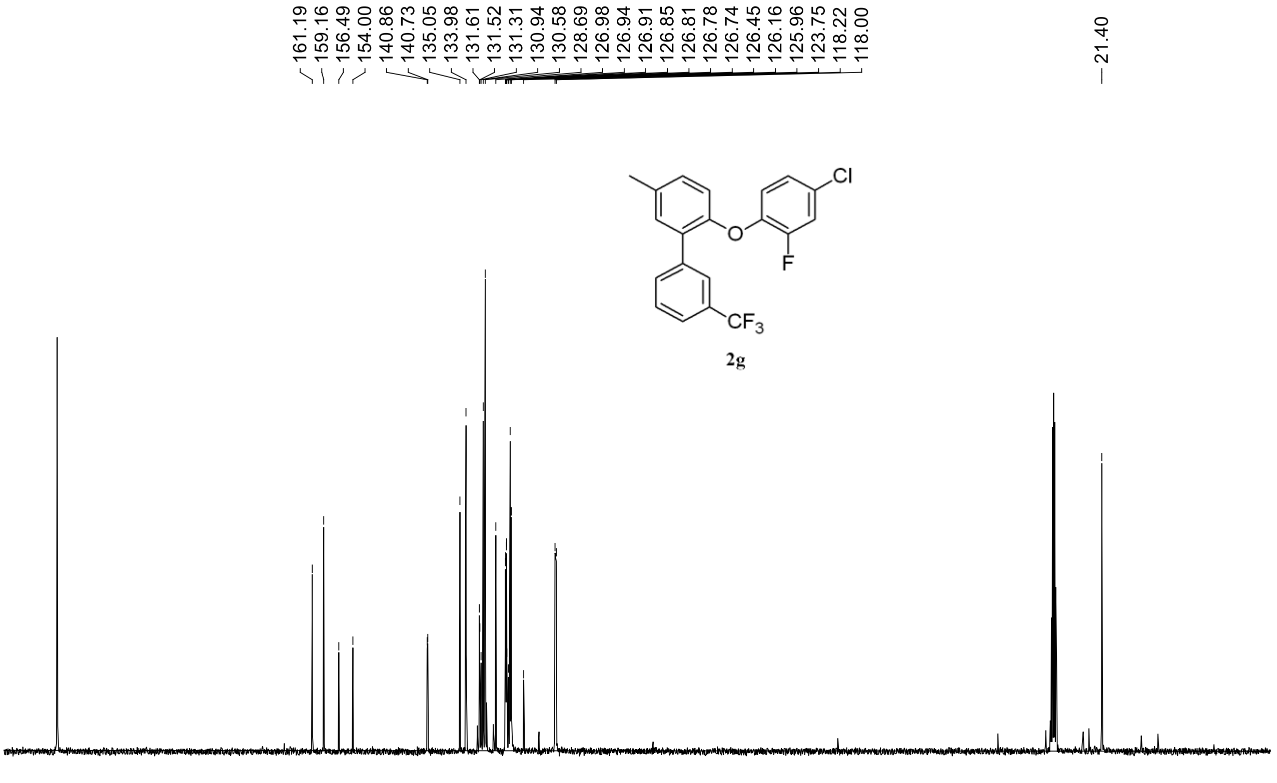
161.19
159.16
156.49
154.00
140.86
140.73
135.05
133.98
131.61
131.52
131.31
130.94
130.58
128.69
126.98
126.94
126.91
126.85
126.81
126.78
126.74
126.45
126.16
125.96
123.75
118.22
118.00
21.40
O
Cl
F
CF3
2g
210 200 190 180 170 160 150 140 130 120 110 100 90 80 70 60 50 40 30 20 10 0
f1 (ppm)

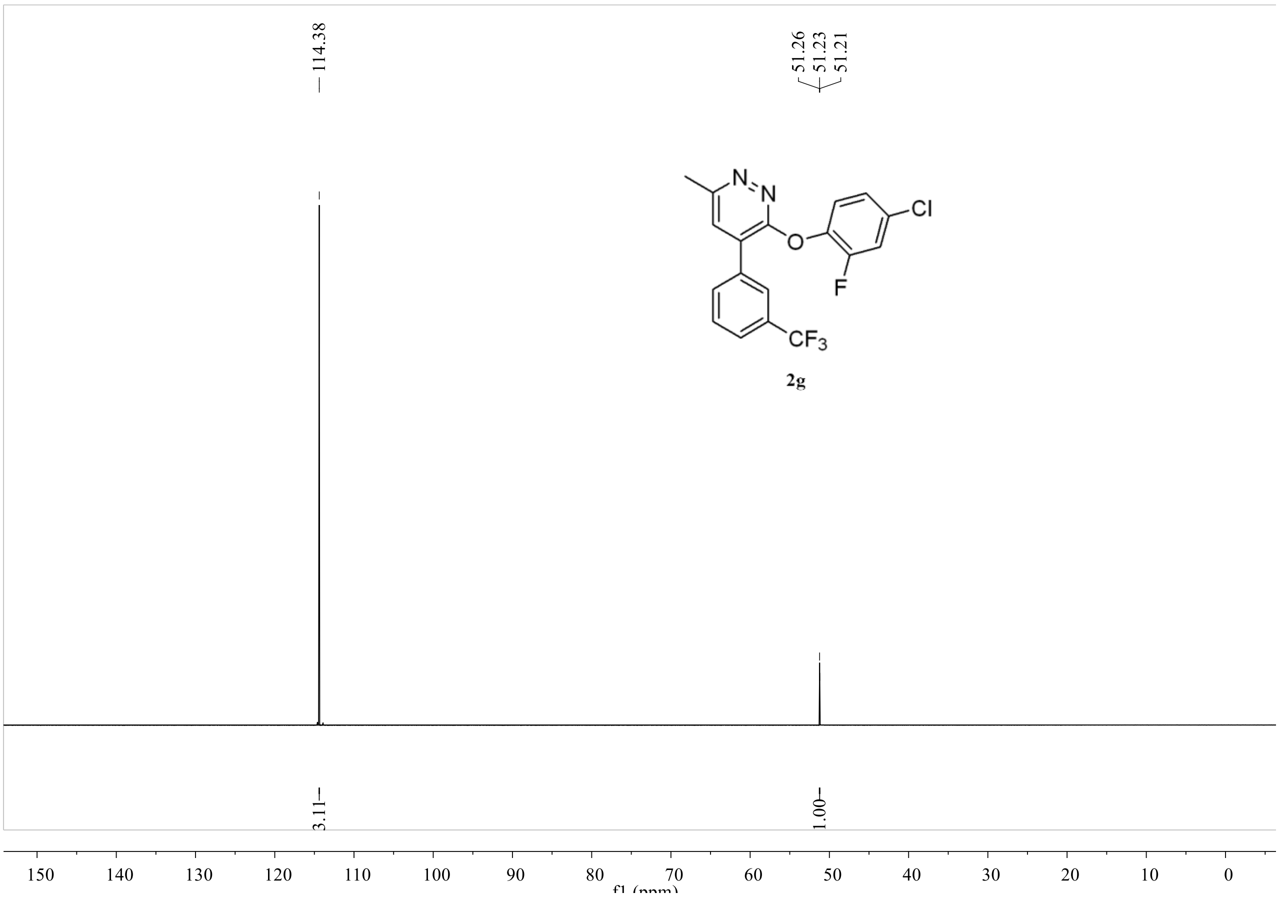
114.38
51.26
51.23
51.21
CF3
2g
3.11
1.00
150
140
130
120
110
100
90
80
70
60
50
40
30
20
10
0
f1 (ppm)

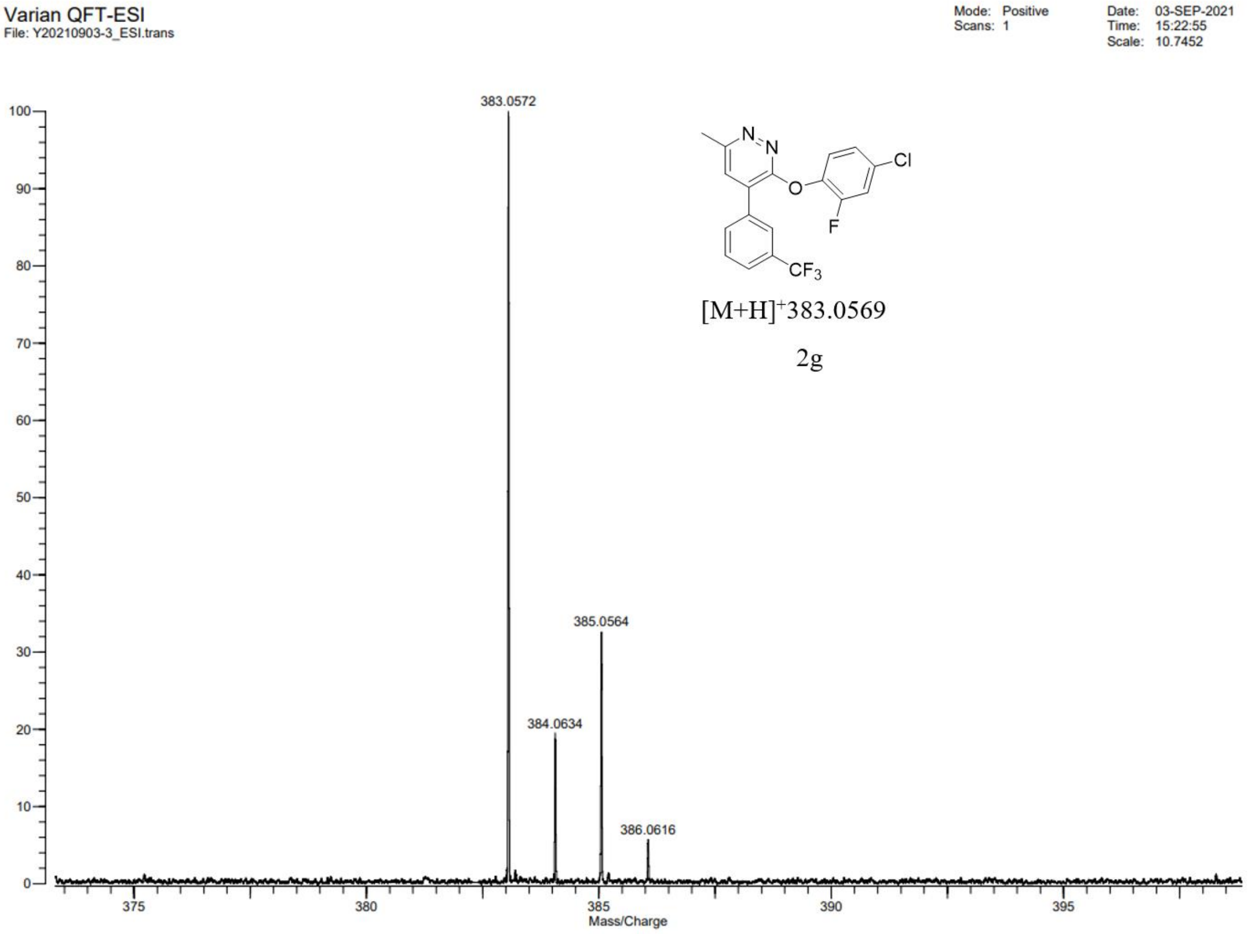
Varian QFT-ESI
File: Y20210903-3_ESI.trans
Mode: Positive
Scans: 1
Date: 03-SEP-2021
Time: 15:22:55
Scale: 10.7452
383.0572
384.0634
385.0564
386.0616
[M+H]+383.0569
2g
Mass/Charge

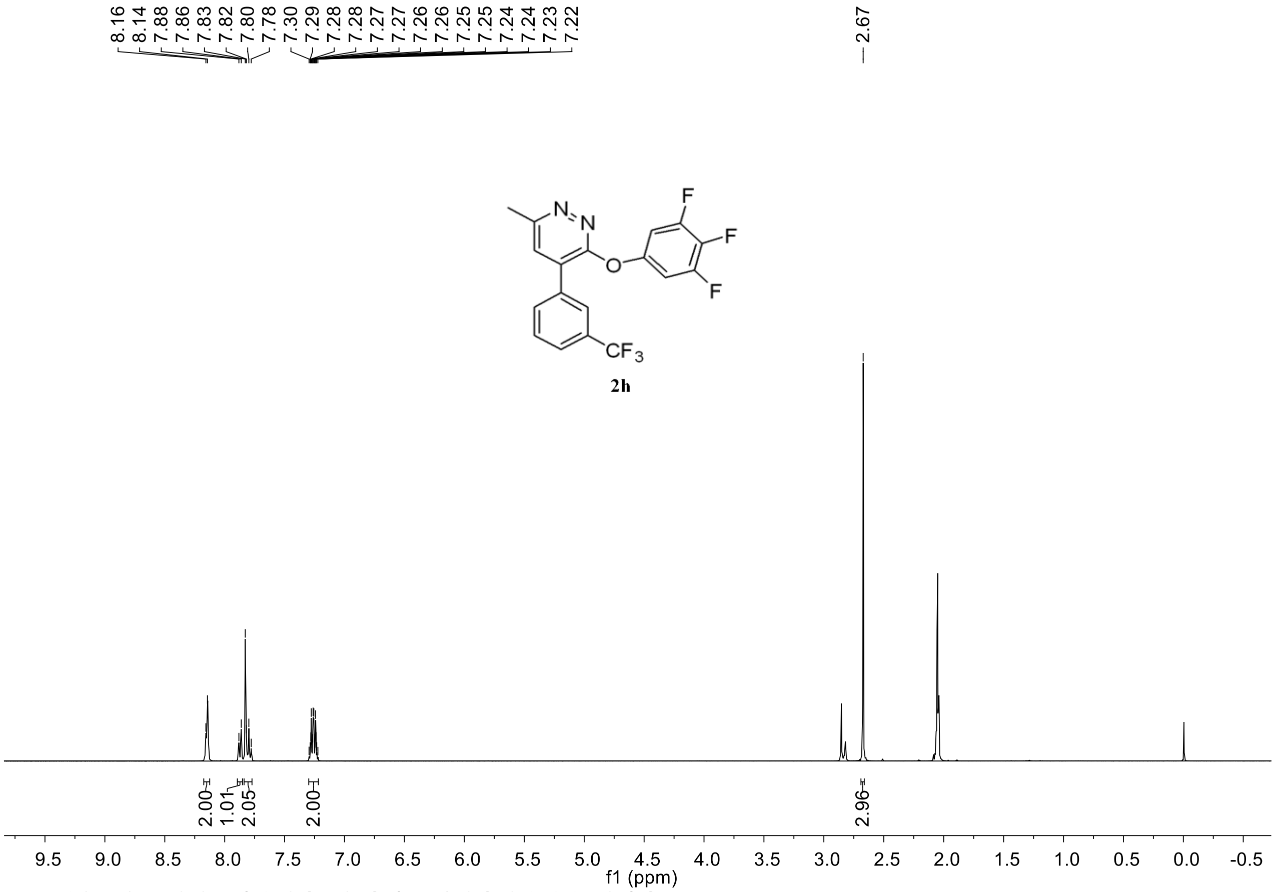
8.16
8.14
7.88
7.86
7.83
7.82
7.80
7.78
7.30
7.29
7.28
7.28
7.27
7.27
7.26
7.26
7.25
7.25
7.24
7.24
7.23
7.22
2.67
F
F
F
N
N
O
CF3
2h
2.00
1.01
2.05
2.00
2.96
9.5 9.0 8.5 8.0 7.5 7.0 6.5 6.0 5.5 5.0 4.5 4.0 3.5 3.0 2.5 2.0 1.5 1.0 0.5 0.0 -0.5
f1 (ppm)

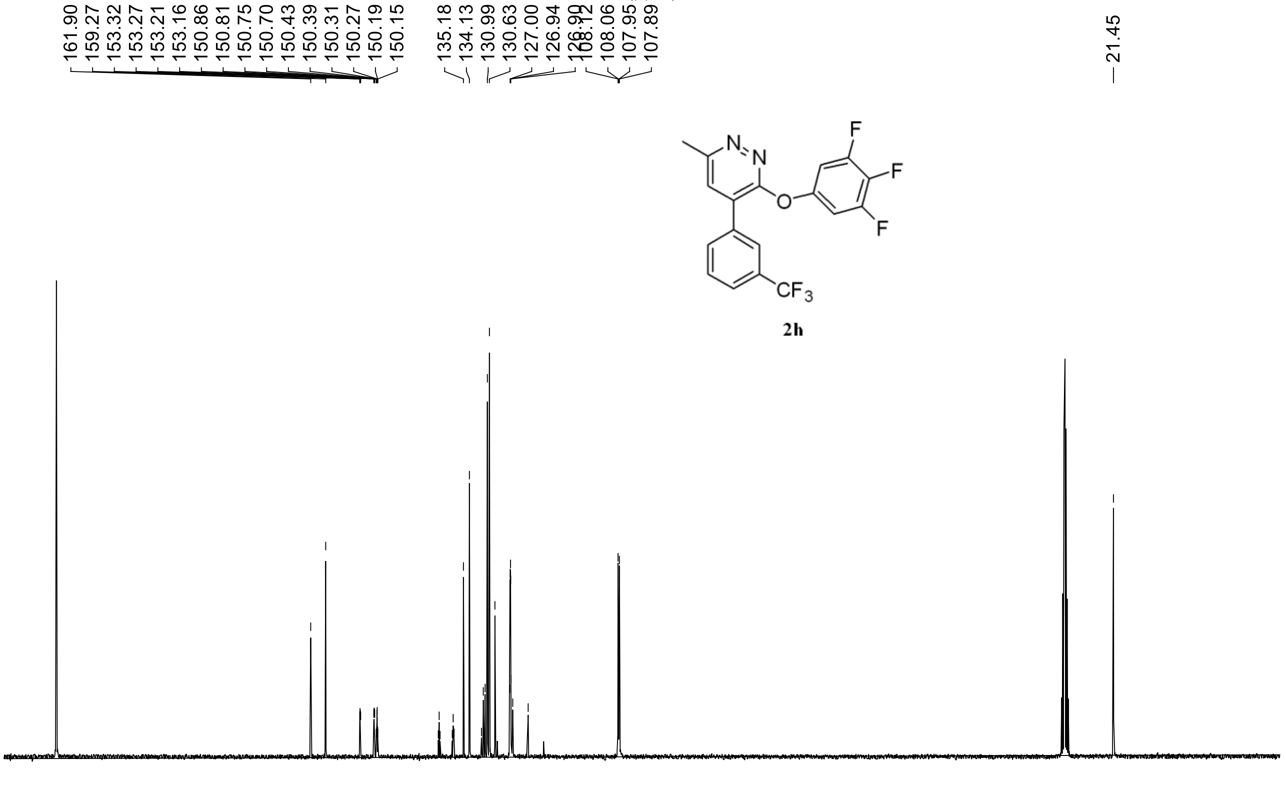
161.90
159.27
153.32
153.27
153.21
153.16
150.86
150.81
150.75
150.70
150.43
150.39
150.31
150.27
150.19
150.15
135.18
134.13
130.99
130.63
127.00
126.94
126.90
108.12
108.06
107.95
107.89
21.45
F
F
F
N
N
O
CF3
2h
210 200 190 180 170 160 150 140 130 120 110 100 90 80 70 60 50 40 30 20 10 0
f1 (ppm)

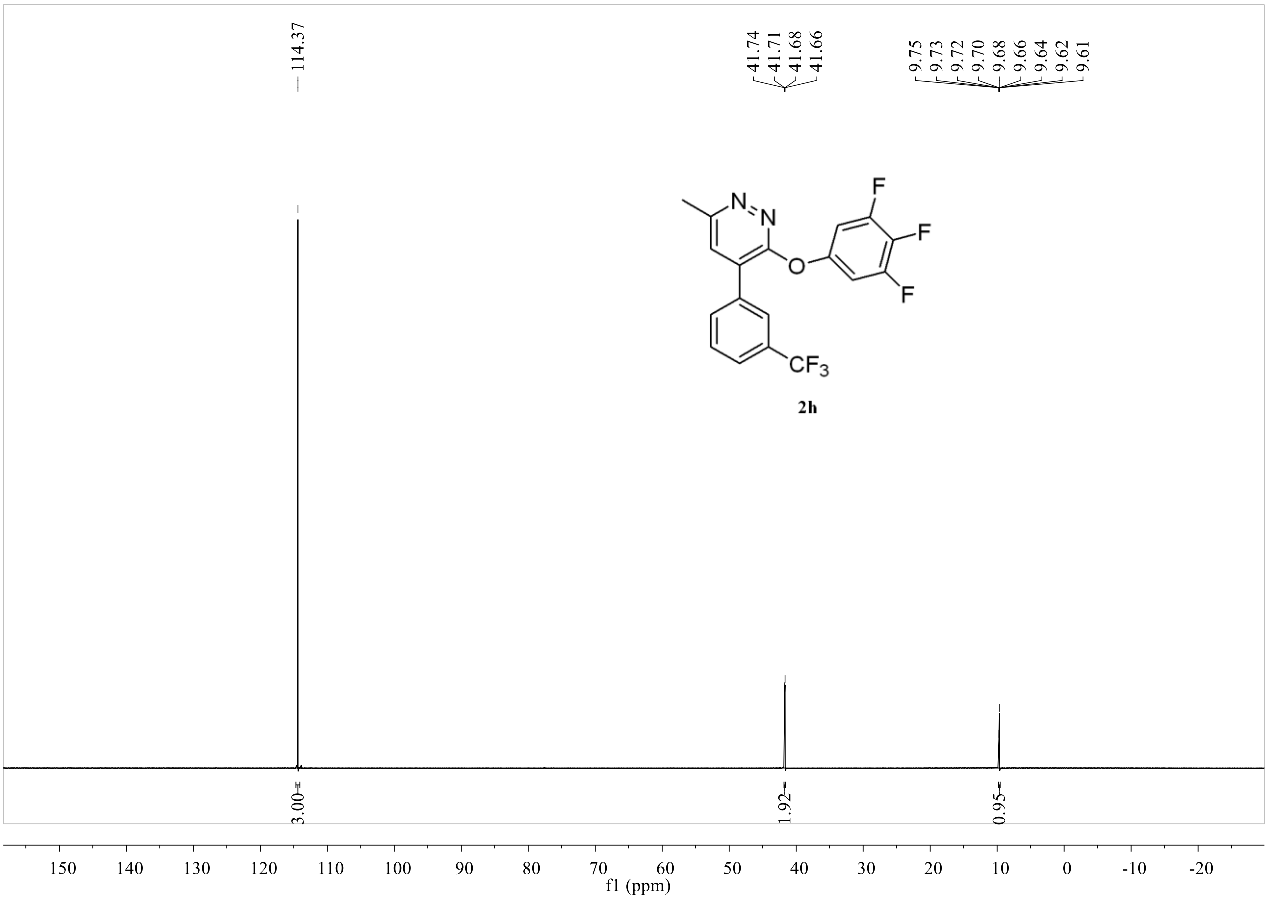

114.37
41.74
41.71
41.68
41.66
9.75
9.73
9.72
9.70
9.68
9.66
9.64
9.62
9.61
2h
3.00
1.92
0.95
150
140
130
120
110
100
90
80
70
60
50
40
30
20
10
0
-10
-20
f1 (ppm)

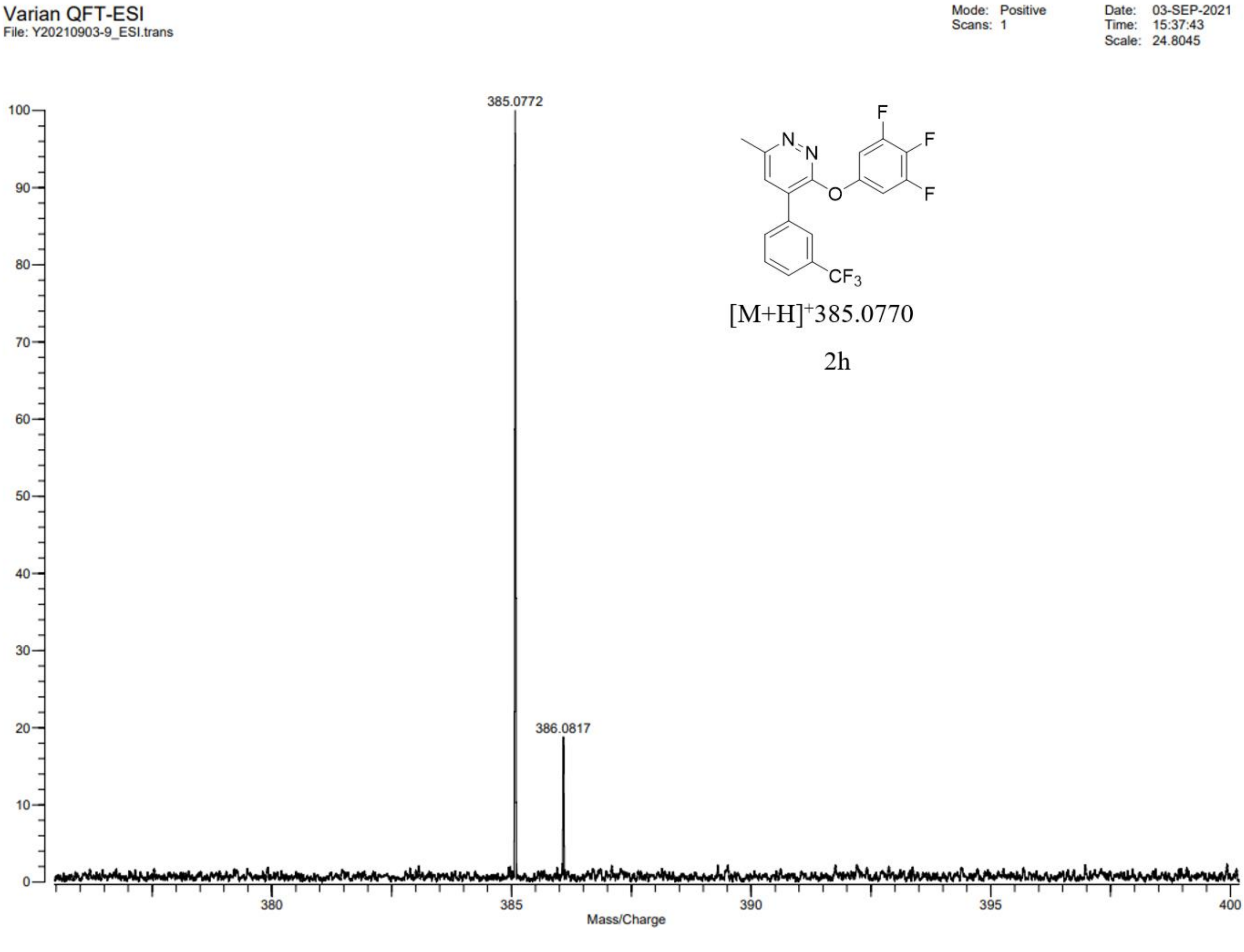

Varian QFT-ESI
File: Y20210903-9_ESI.trans
Mode: Positive
Scans: 1
Date: 03-SEP-2021
Time: 15:37:43
Scale: 24.8045
385.0772
386.0817
[M+H]+385.0770
2h
Mass/Charge

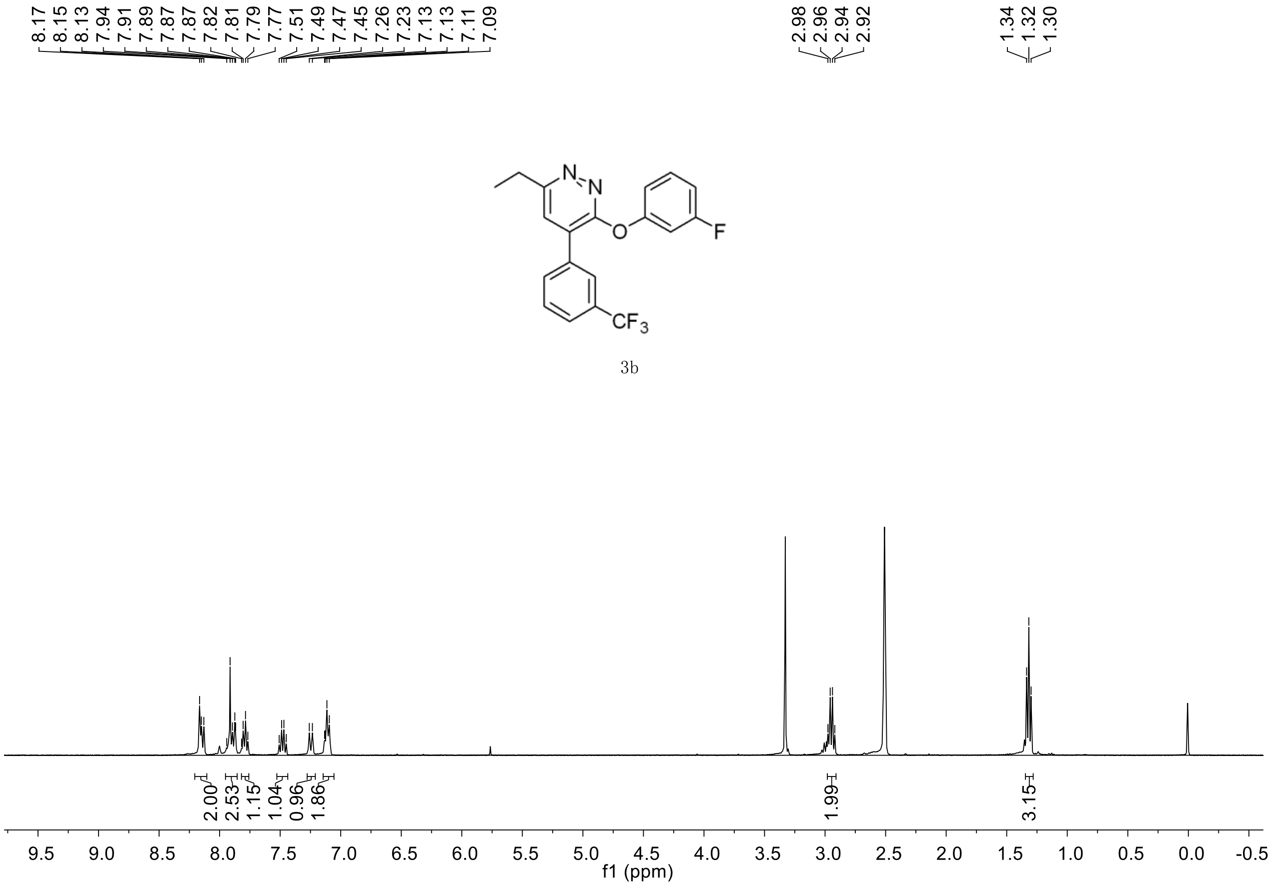
8.17 8.15 8.13 7.94 7.91 7.89 7.87 7.87 7.82 7.81 7.79 7.77 7.51 7.49 7.47 7.45 7.26 7.23 7.13 7.11 7.09
2.98 2.96 2.94 2.92
1.34 1.32 1.30
3b
2.00 2.53 1.15 1.04 0.96 1.86
1.99
3.15
f1 (ppm)

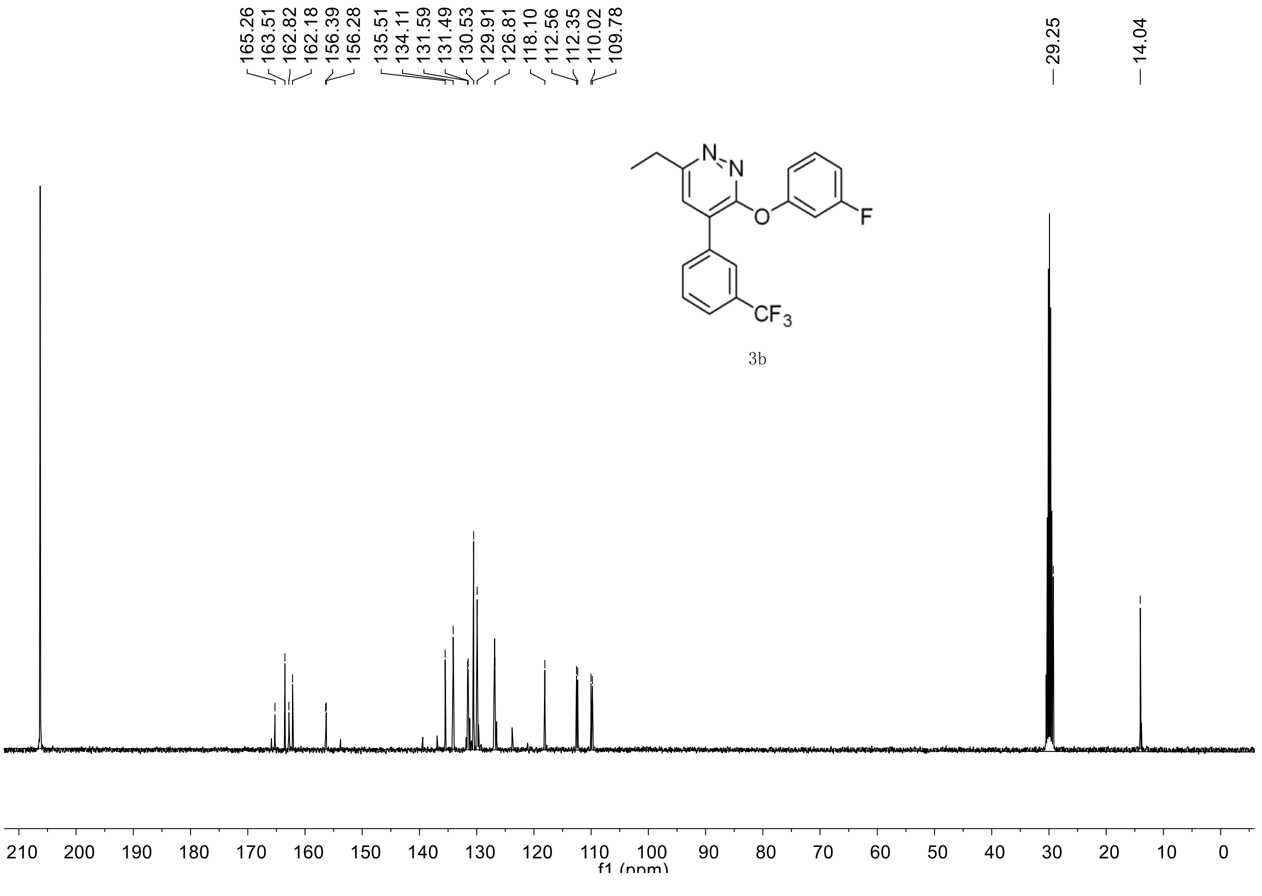
165.26 163.51 162.82 162.18 156.39 156.28 135.51 134.11 131.59 131.49 130.53 129.91 126.81 118.10 112.56 112.35 110.02 109.78
29.25
14.04
3b
f1 (ppm)

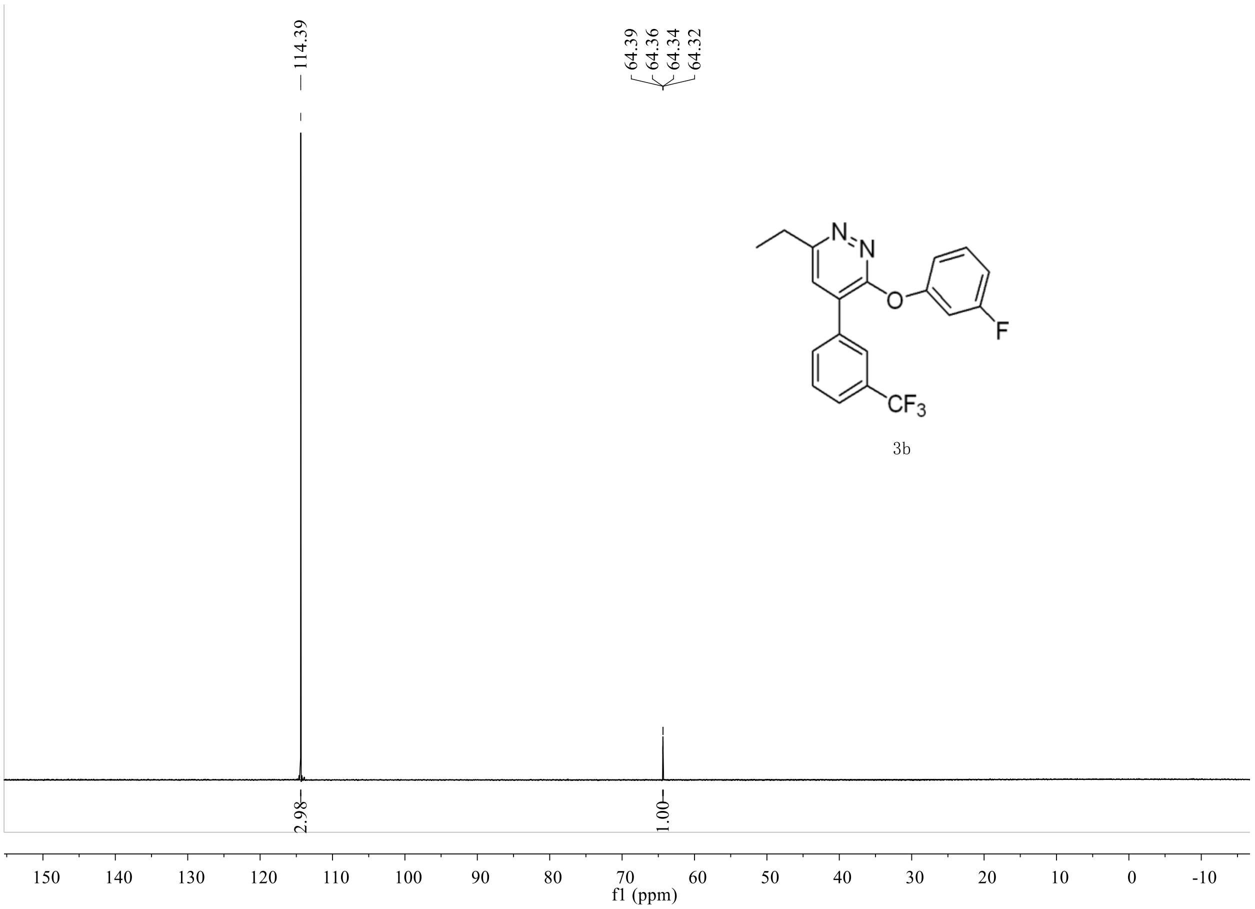
114.39
64.39
64.36
64.34
64.32
N
N
O
F
CF3
3b
2.98
1.00
150 140 130 120 110 100 90 80 70 60 50 40 30 20 10 0 -10
f1 (ppm)

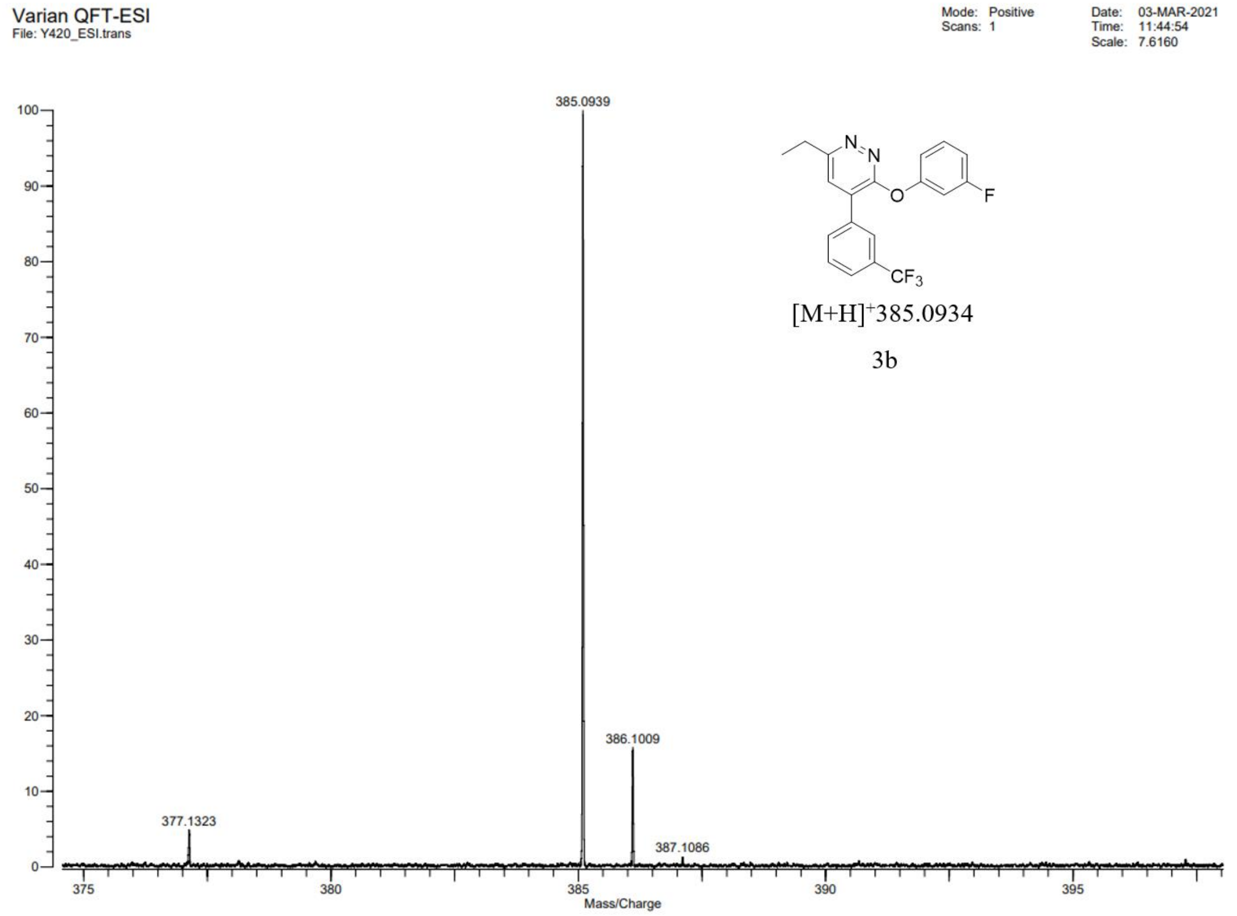
Varian QFT-ESI
File: Y420_ESI.trans
Mode: Positive
Scans: 1
Date: 03-MAR-2021
Time: 11:44:54
Scale: 7.6160
385.0939
386.1009
377.1323
387.1086
N
N
O
F
CF3
[M+H]+385.0934
3b
Mass/Charge
0 10 20 30 40 50 60 70 80 90 100
375 380 385 390 395

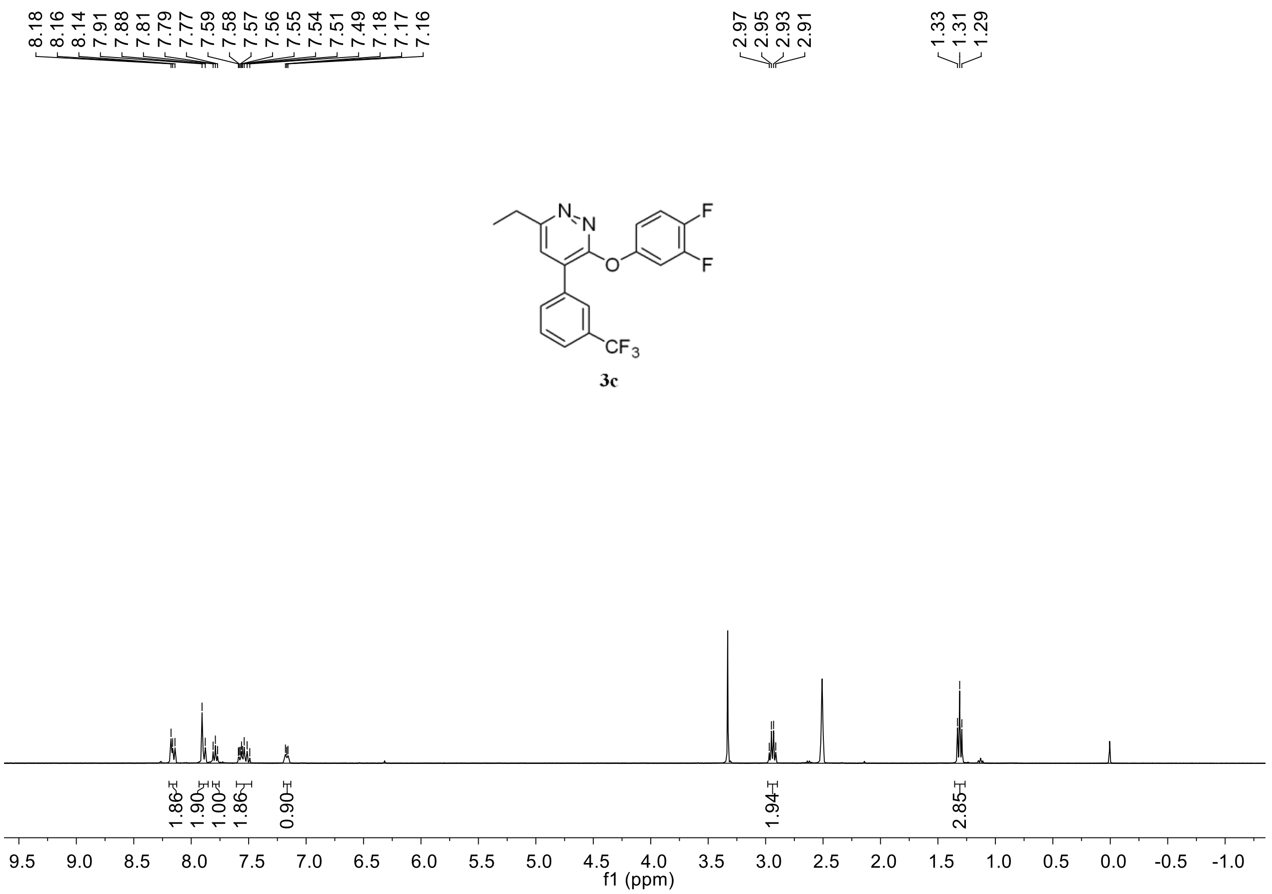
8.18 8.16 8.14 7.91 7.88 7.81 7.79 7.77 7.59 7.58 7.57 7.56 7.55 7.54 7.51 7.49 7.18 7.17 7.16
2.97 2.95 2.93 2.91
1.33 1.31 1.29
N N O F F CF3
3c
1.86 1.90 1.00 1.86 0.90 1.94 2.85
9.5 9.0 8.5 8.0 7.5 7.0 6.5 6.0 5.5 5.0 4.5 4.0 3.5 3.0 2.5 2.0 1.5 1.0 0.5 0.0 -0.5 -1.0
f1 (ppm)

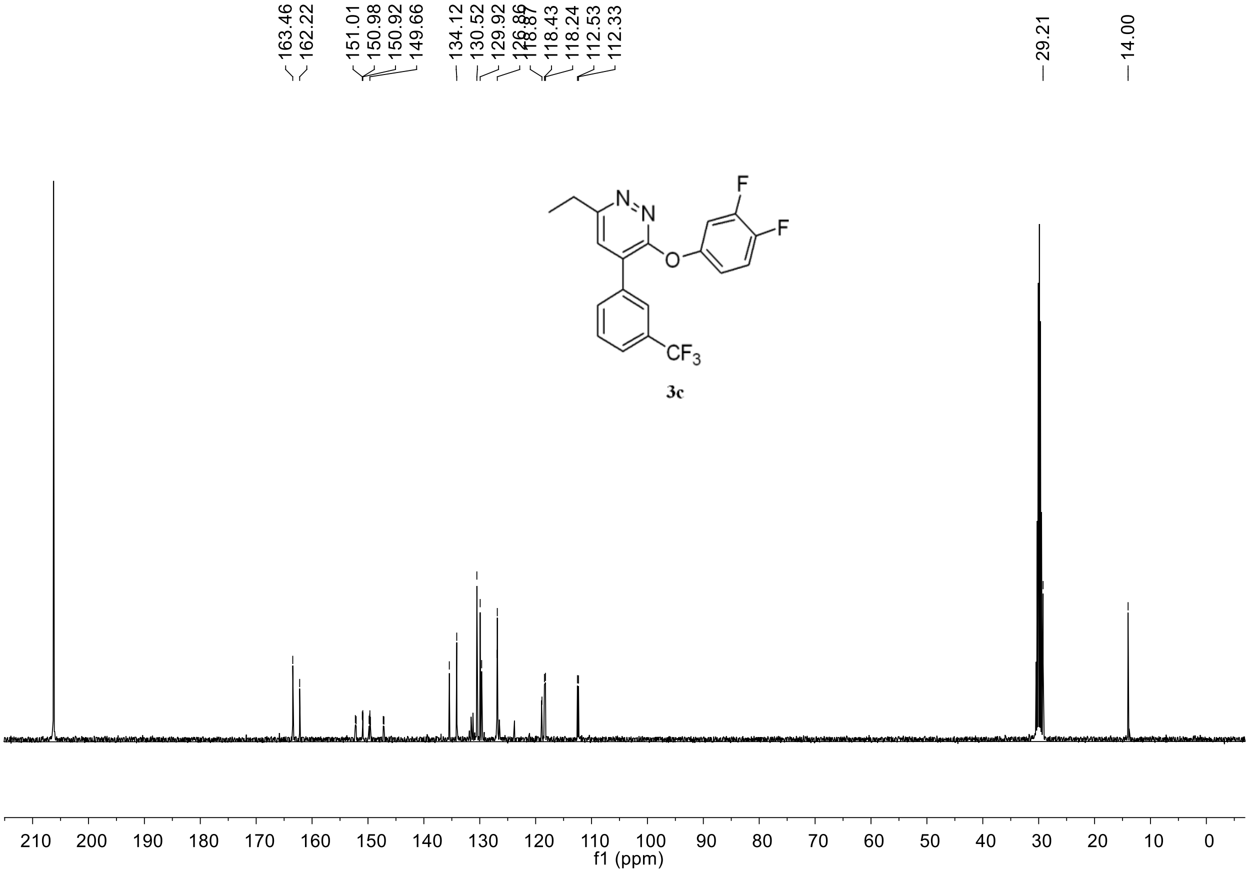
163.46 162.22 151.01 150.98 150.92 149.66 134.12 130.52 129.92 126.86 118.87 118.43 118.24 112.53 112.33
29.21 14.00
N N O F F CF3
3c
210 200 190 180 170 160 150 140 130 120 110 100 90 80 70 60 50 40 30 20 10 0
f1 (ppm)

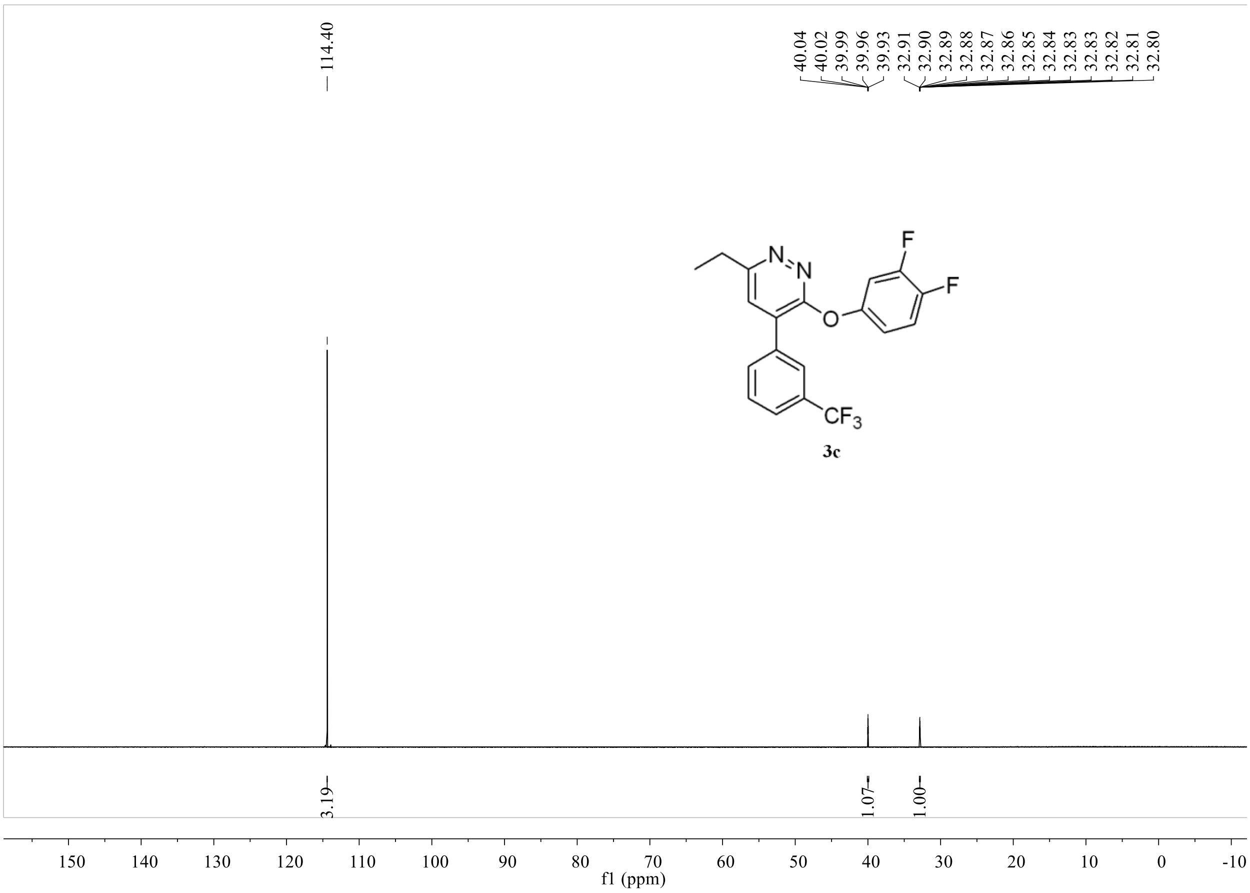

114.40
40.04
40.02
39.99
39.96
39.93
32.91
32.90
32.89
32.88
32.87
32.86
32.85
32.84
32.83
32.83
32.82
32.81
32.80
N
N
O
F
F
CF3
3c
3.19
1.07
1.00
150 140 130 120 110 100 90 80 70 60 50 40 30 20 10 0 -10
f1 (ppm)

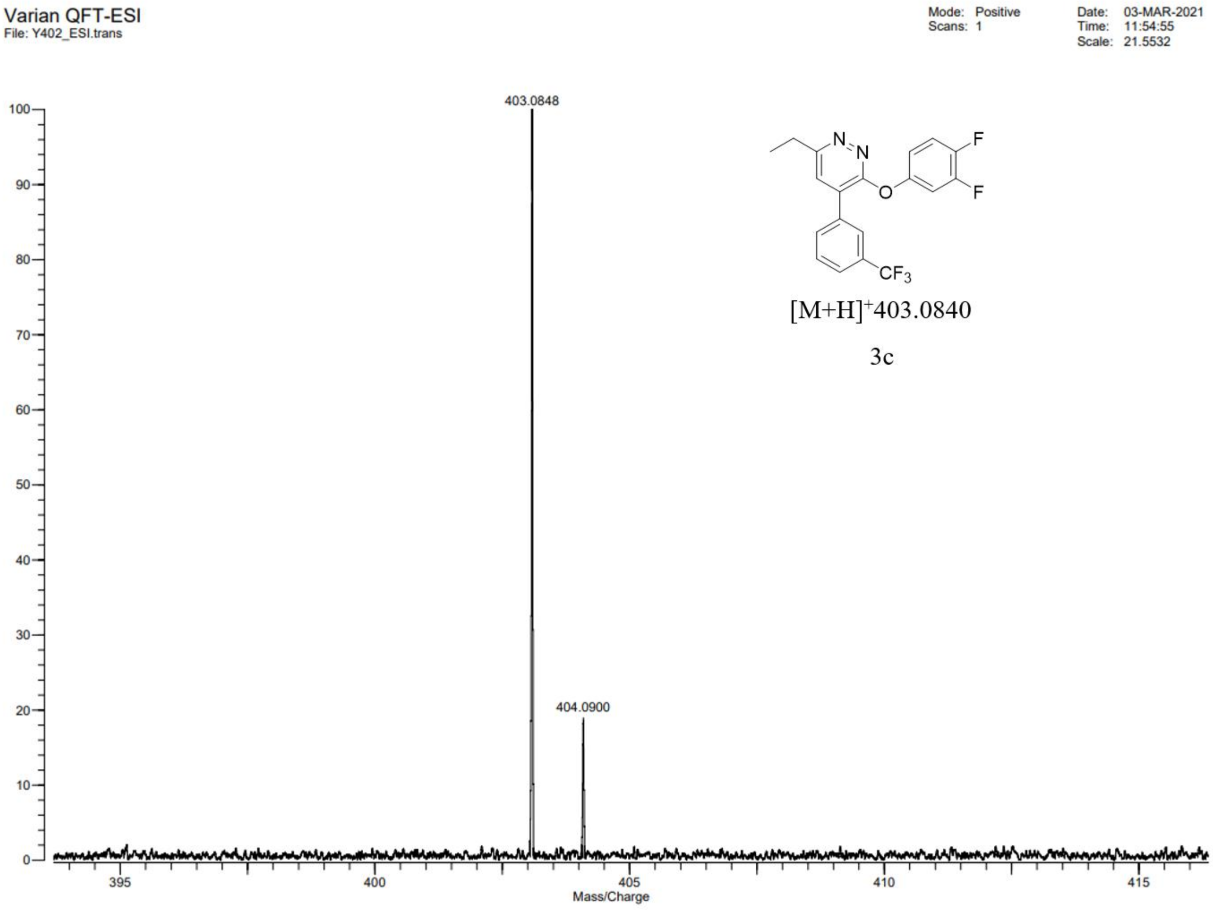

Varian QFT-ESI
File: Y402_ESI.trans
Mode: Positive
Scans: 1
Date: 03-MAR-2021
Time: 11:54:55
Scale: 21.5532
403.0848
404.0900
N
N
O
F
F
CF3
[M+H]+403.0840
3c
Mass/Charge

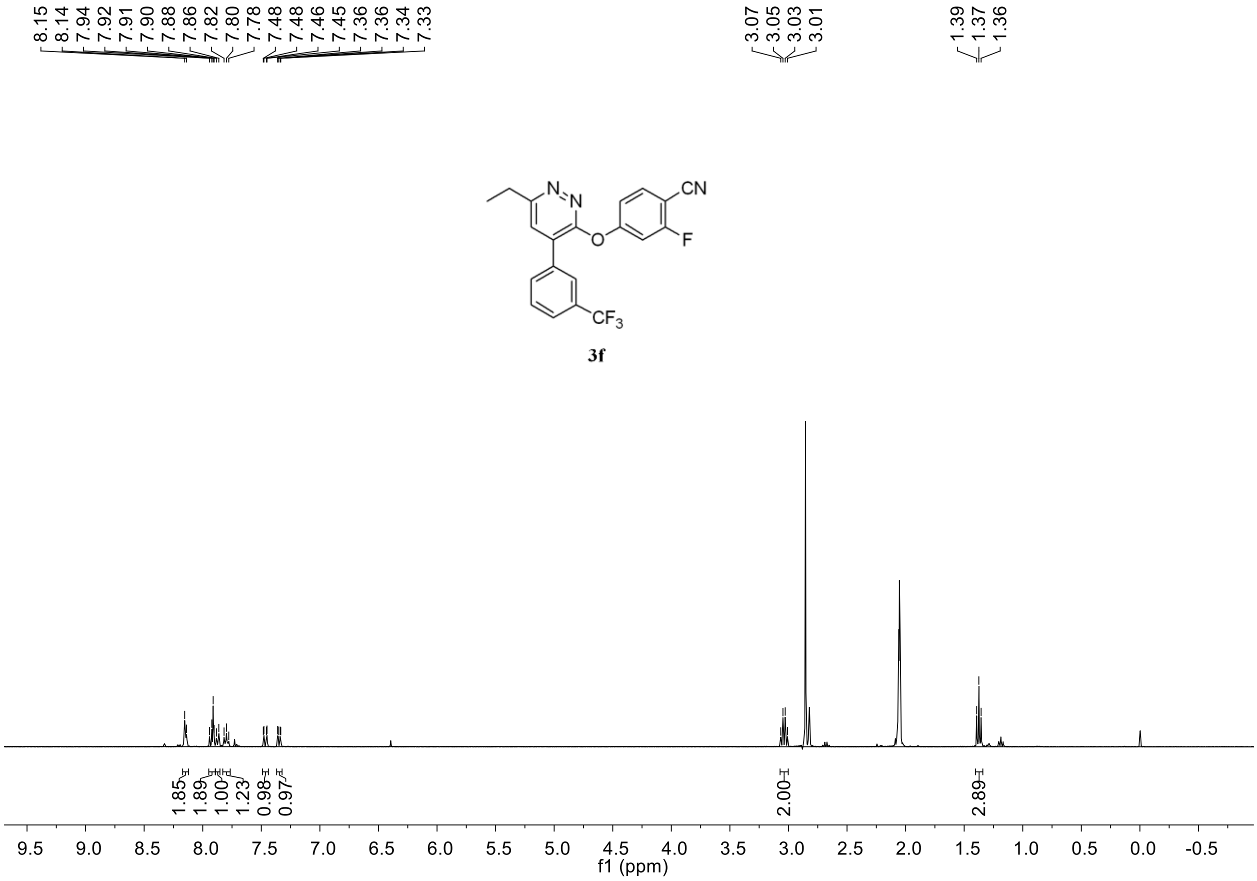
8.15
8.14
7.94
7.92
7.91
7.90
7.88
7.86
7.82
7.80
7.78
7.48
7.48
7.46
7.45
7.36
7.36
7.34
7.33
3.07
3.05
3.03
3.01
1.39
1.37
1.36
CN
F
CF3
3f
1.85
1.89
1.00
1.23
0.98
0.97
2.00
2.89
f1 (ppm)

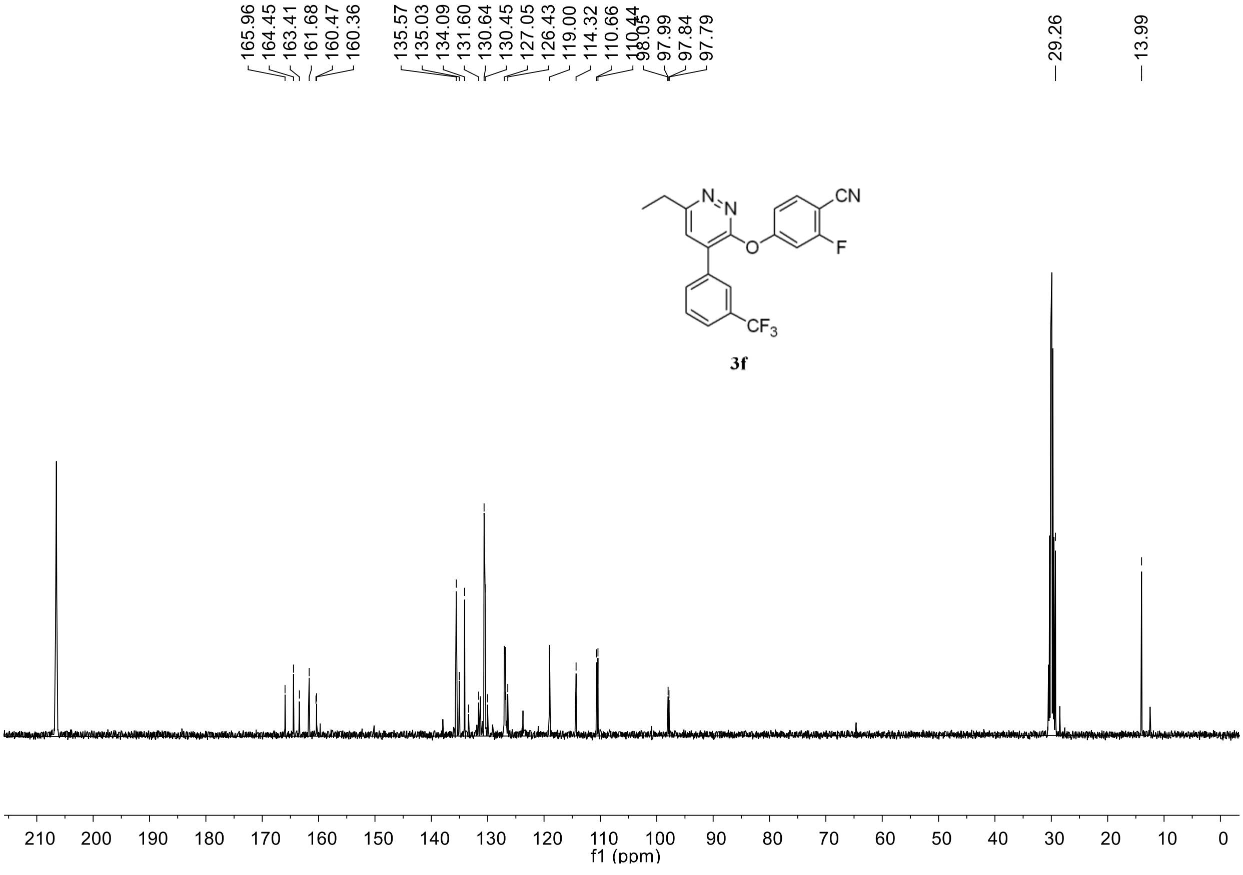
165.96
164.45
163.41
161.68
160.47
160.36
135.57
135.03
134.09
131.60
130.64
130.45
127.05
126.43
119.00
114.32
110.66
110.44
98.05
97.99
97.84
97.79
29.26
13.99
CN
F
CF3
3f
f1 (ppm)

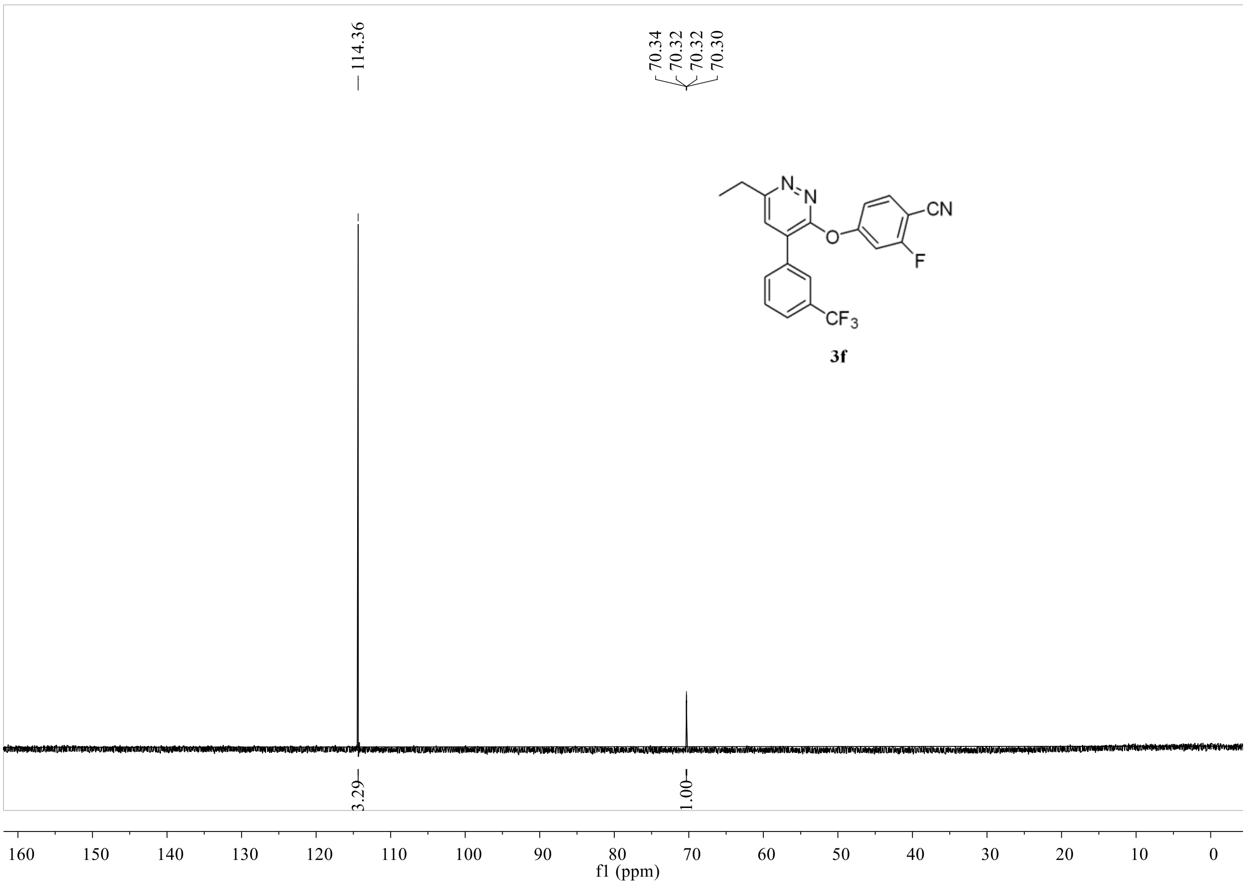
114.36
70.34
70.32
70.32
70.30
CN
F
O
N
N
CF3
3f
3.29
1.00
160
150
140
130
120
110
100
90
80
70
60
50
40
30
20
10
0
f1 (ppm)

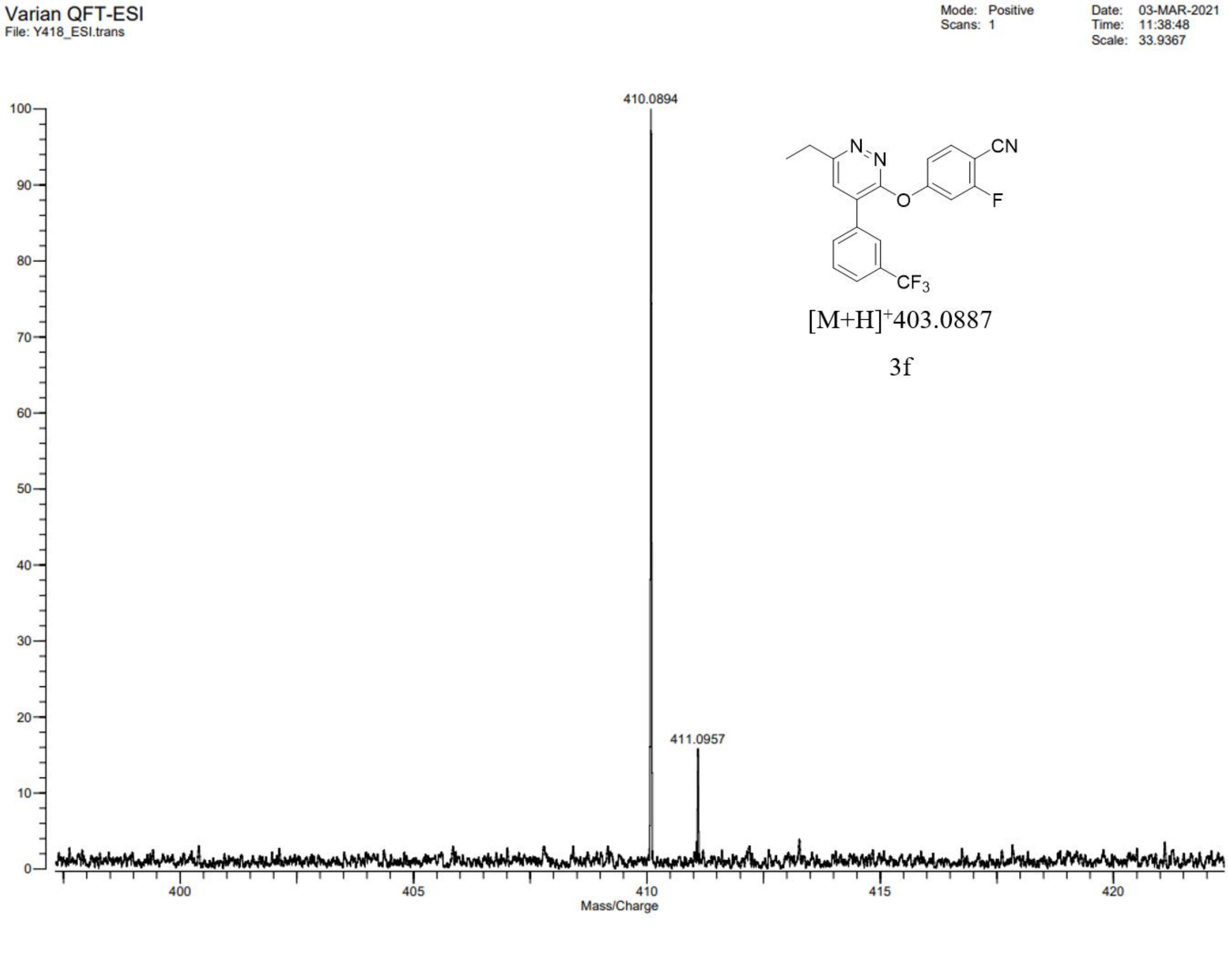
Varian QFT-ESI
File: Y418_ESI.trans
Mode: Positive
Scans: 1
Date: 03-MAR-2021
Time: 11:38:48
Scale: 33.9367
410.0894
411.0957
CN
F
O
N
N
CF3
[M+H]+403.0887
3f
100
90
80
70
60
50
40
30
20
10
0
400
405
410
415
420
Mass/Charge

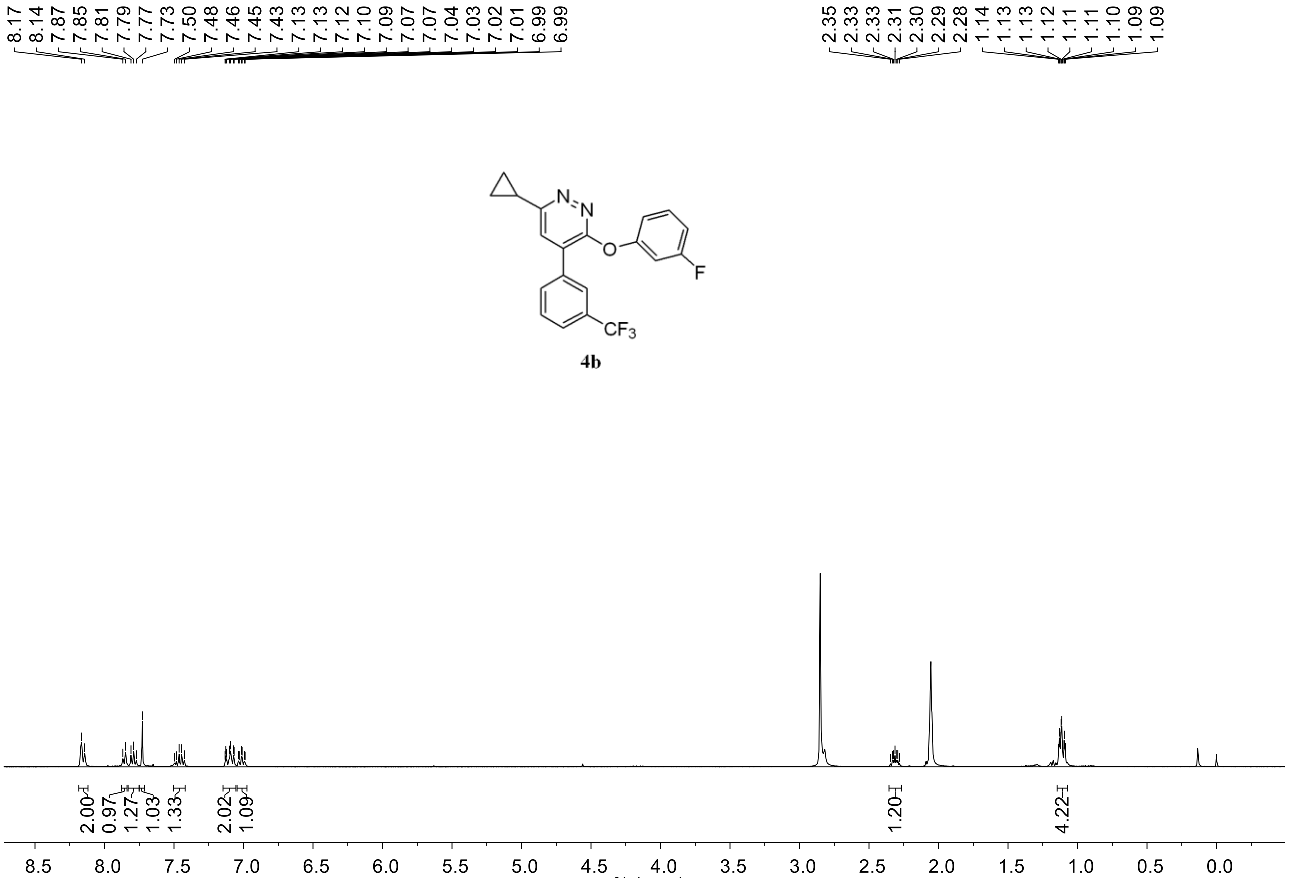
8.17 8.14 7.87 7.85 7.81 7.79 7.77 7.73 7.50 7.48 7.46 7.45 7.43 7.13 7.13 7.12 7.10 7.09 7.07 7.07 7.04 7.03 7.02 7.01 6.99 6.99
2.35 2.33 2.33 2.31 2.30 2.29 2.28 1.14 1.13 1.13 1.12 1.11 1.11 1.10 1.09 1.09
N
N
O
F
CF3
4b
2.00 0.97 1.27 1.03 1.33 2.02 1.09 1.20 4.22
f1 (ppm)

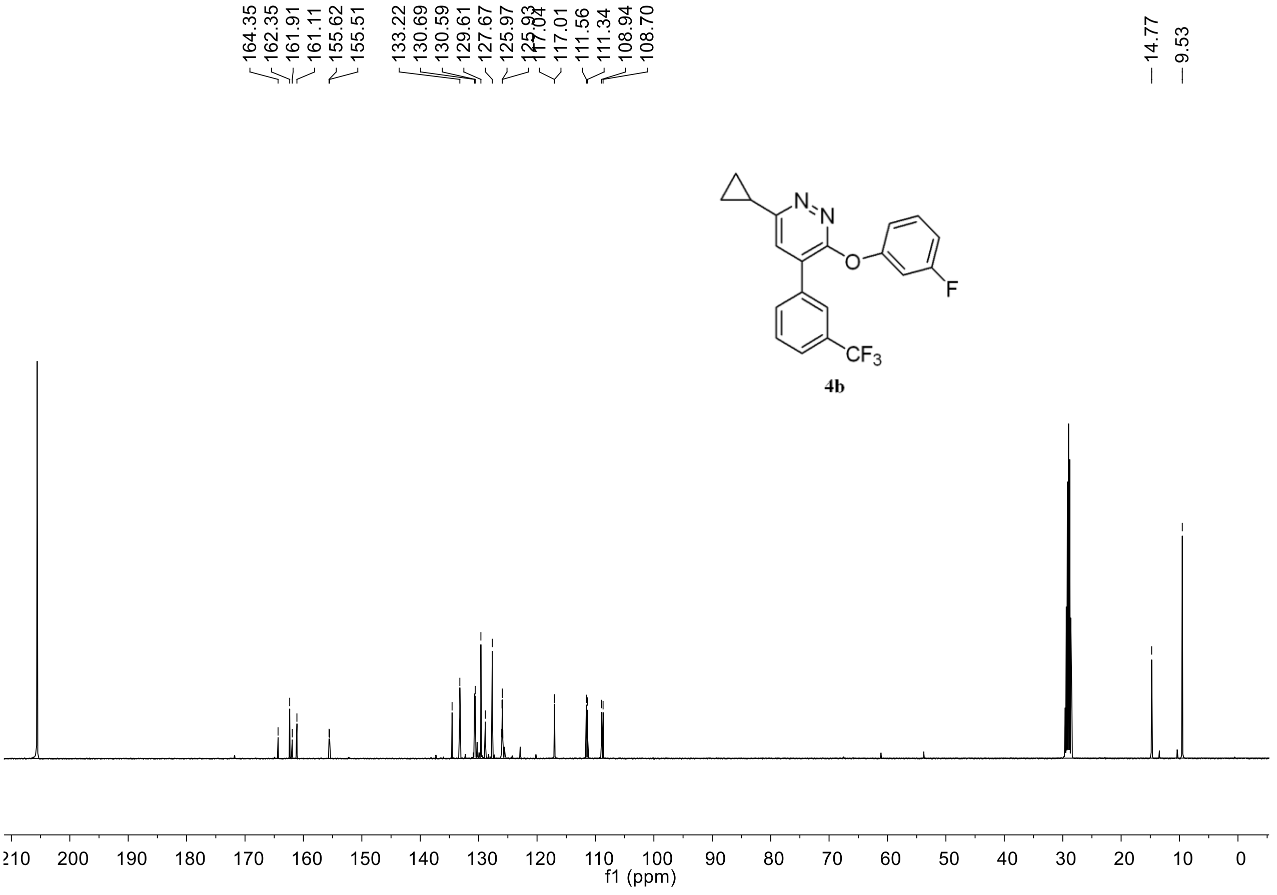
164.35 162.35 161.91 161.11 155.62 155.51 133.22 130.69 130.59 129.61 127.67 125.97 125.93 117.04 117.01 111.56 111.34 108.94 108.70
14.77 9.53
N
N
O
F
CF3
4b
f1 (ppm)

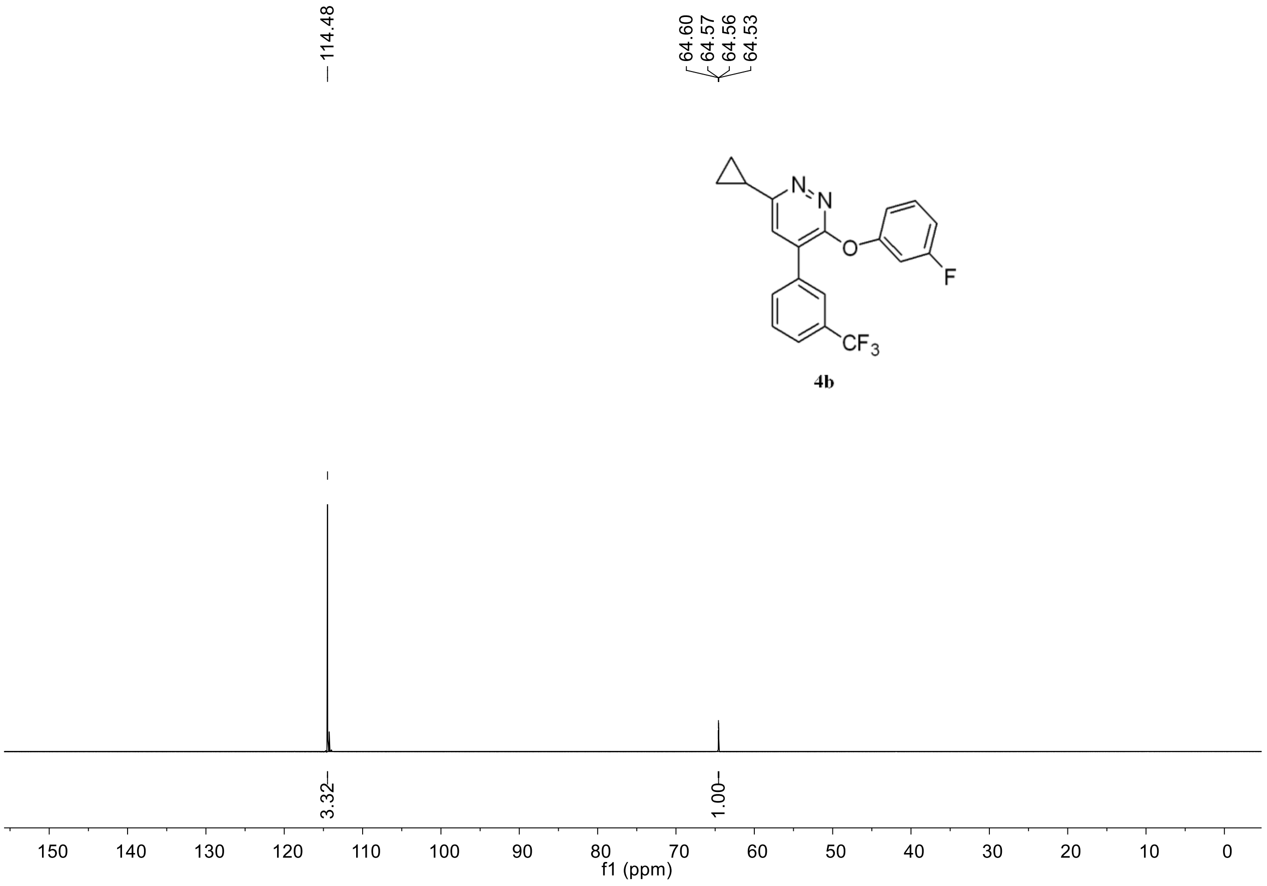

4b

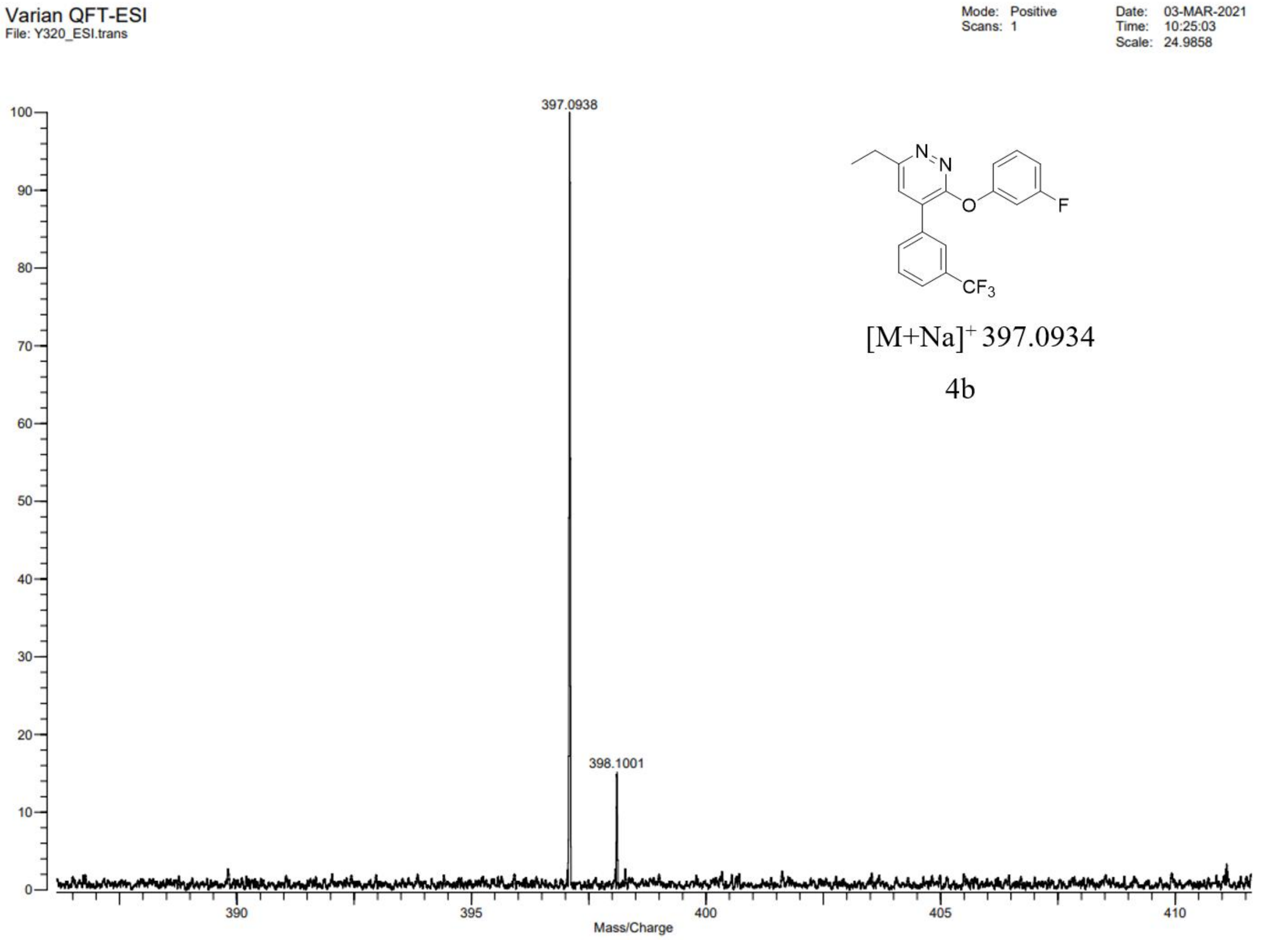

[M+Na]+ 397.0934

4b

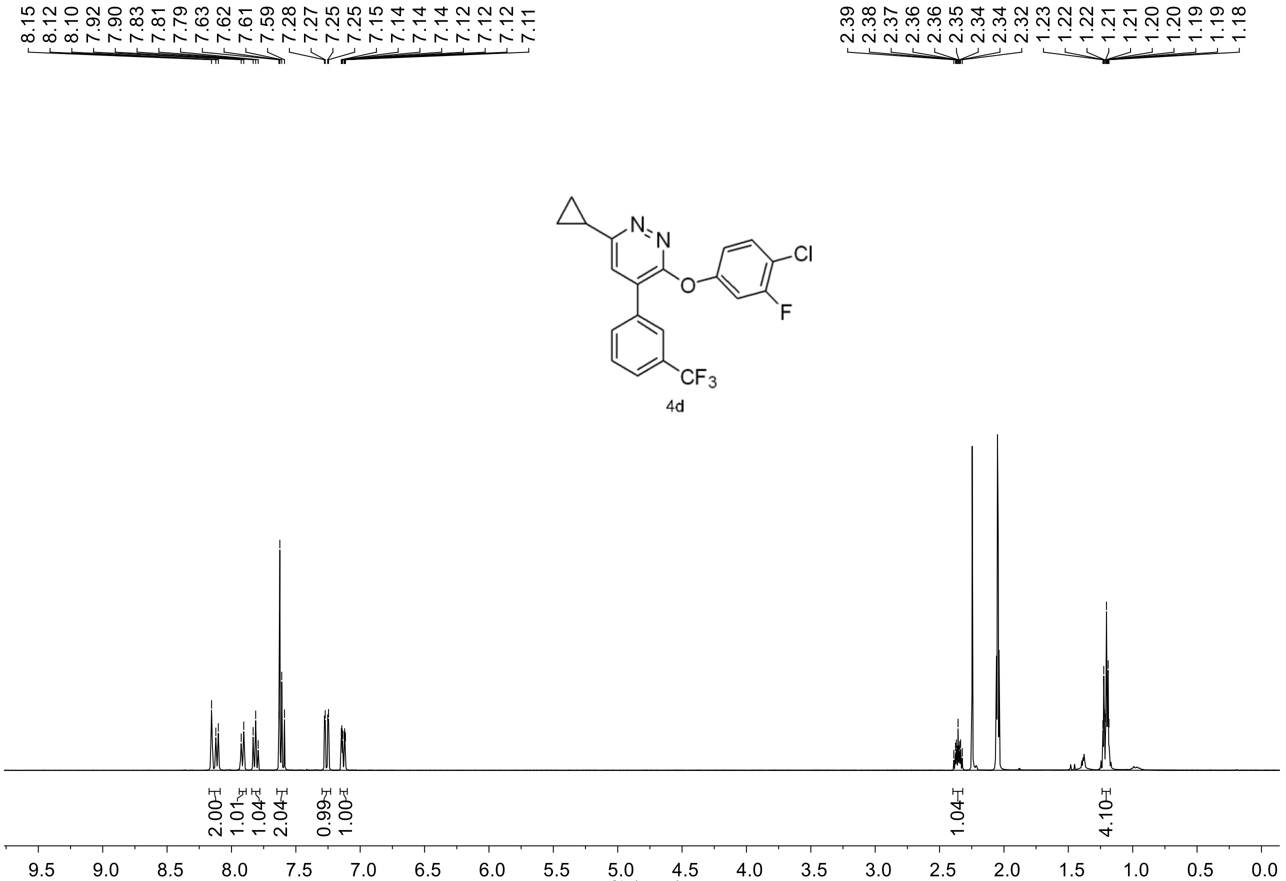
8.15
8.12
8.10
7.92
7.90
7.83
7.81
7.79
7.63
7.62
7.61
7.59
7.28
7.27
7.25
7.25
7.15
7.14
7.14
7.14
7.12
7.12
7.12
7.11
2.39
2.38
2.37
2.36
2.36
2.35
2.34
2.34
2.32
1.23
1.22
1.22
1.21
1.21
1.20
1.20
1.19
1.18
Cl
F
O
CF3
4d
2.00
1.01
1.04
2.04
0.99
1.00
1.04
4.10
f1 (ppm)

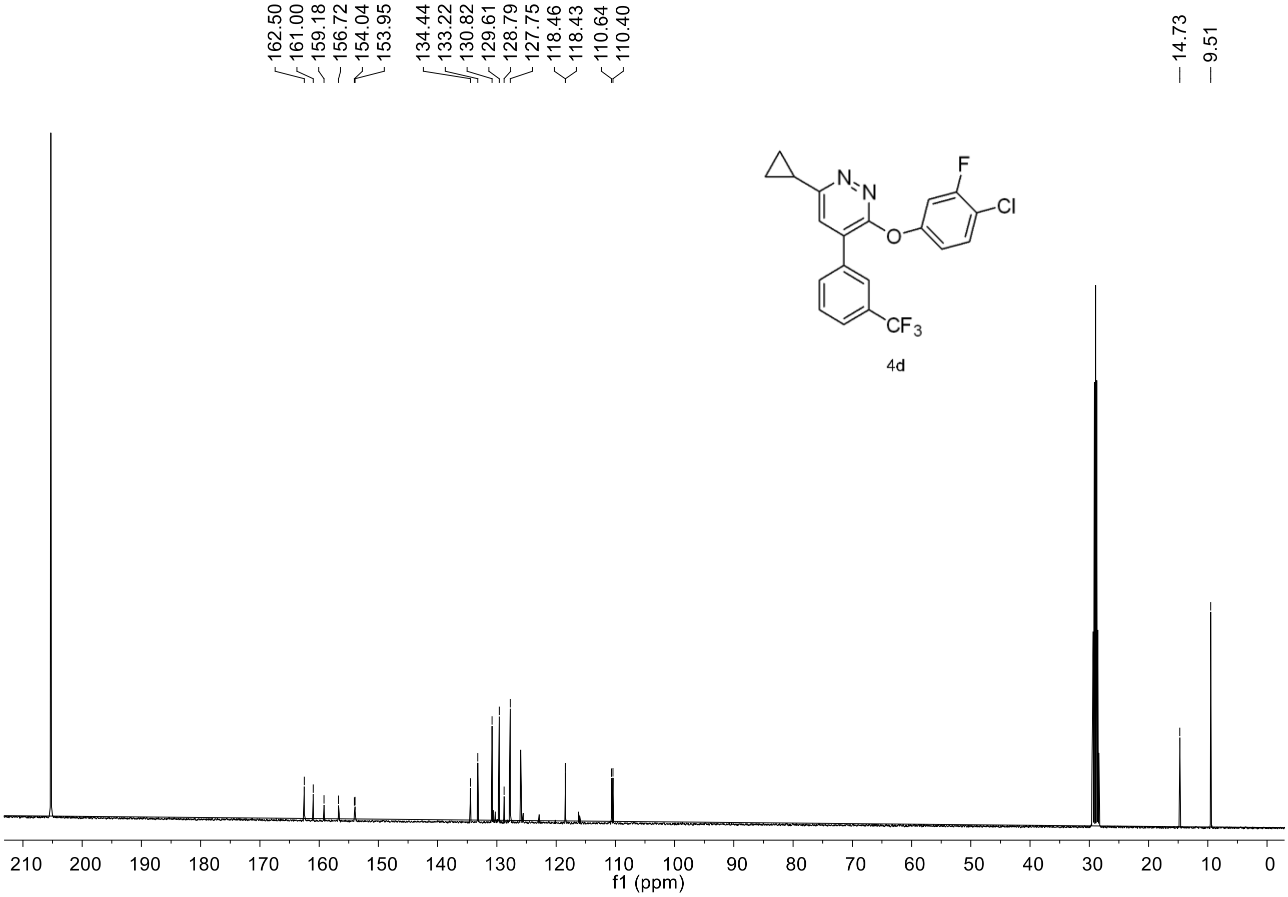
162.50
161.00
159.18
156.72
154.04
153.95
134.44
133.22
130.82
129.61
128.79
127.75
118.46
118.43
110.64
110.40
14.73
9.51
F
Cl
O
CF3
4d
f1 (ppm)

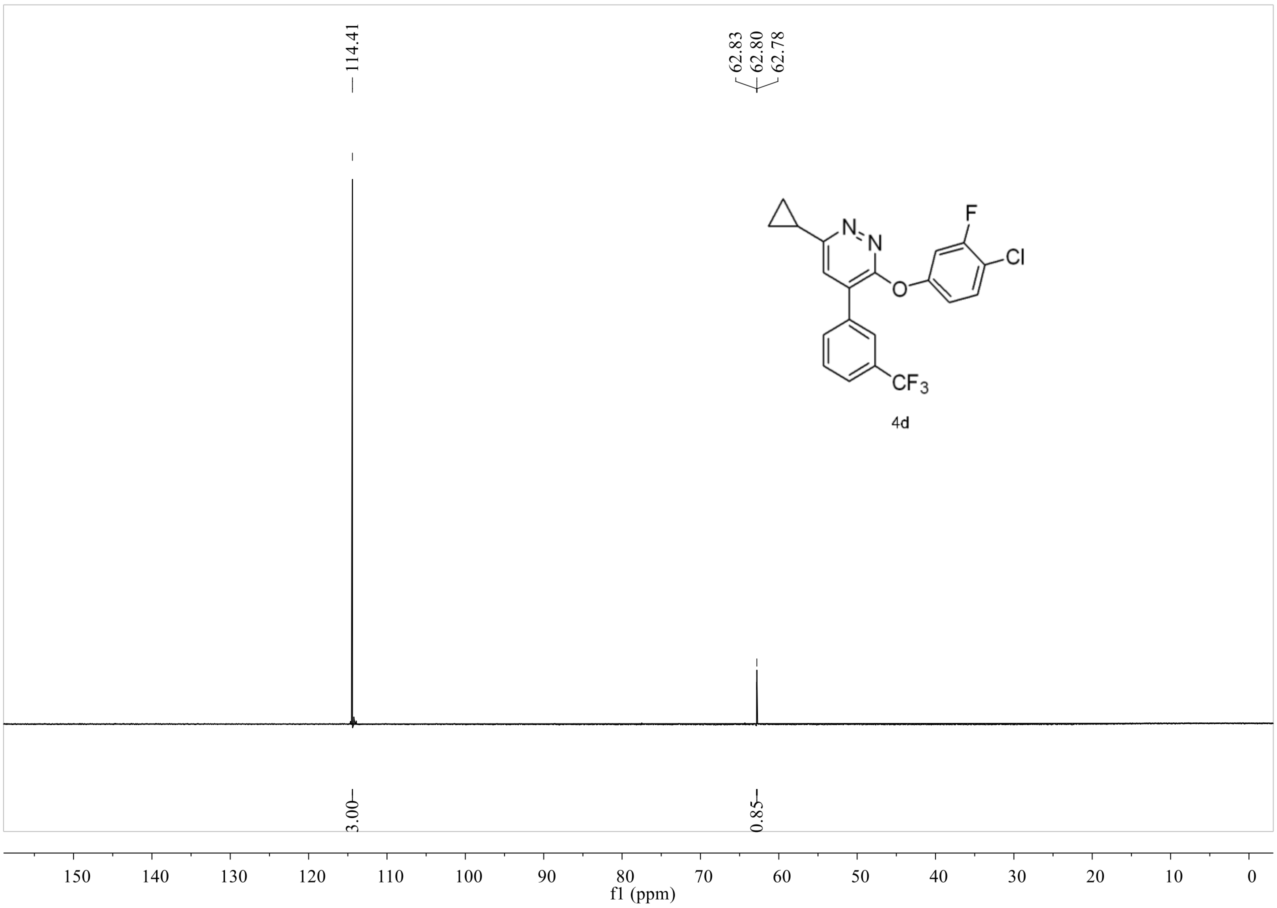

114.41
62.83
62.80
62.78
N
N
F
Cl
O
CF3
4d
3.00
0.85
150
140
130
120
110
100
90
80
70
60
50
40
30
20
10
0
f1 (ppm)

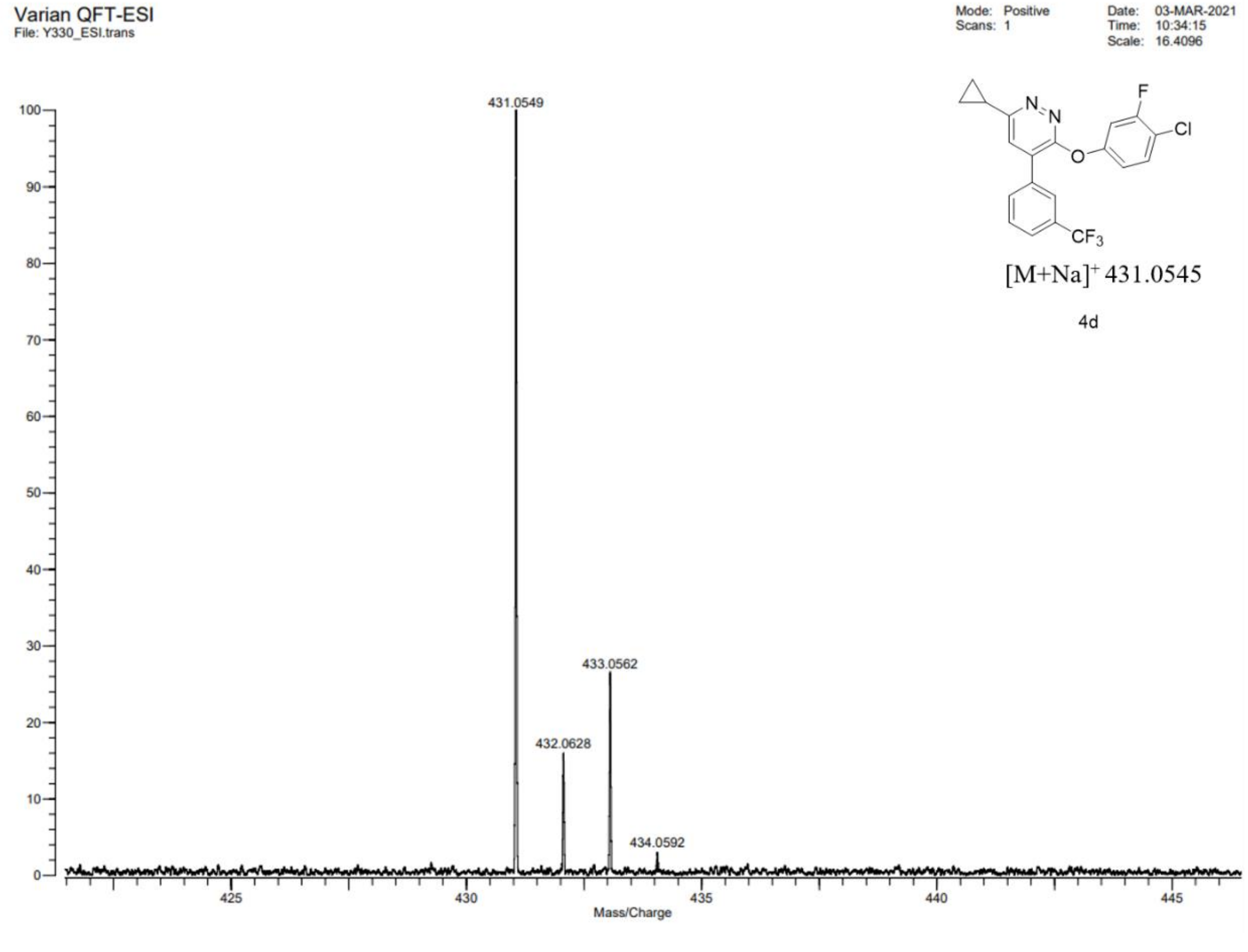

Varian QFT-ESI
File: Y330_ESI.trans
Mode: Positive
Scans: 1
Date: 03-MAR-2021
Time: 10:34:15
Scale: 16.4096
431.0549
432.0628
433.0562
434.0592
[M+Na]+ 431.0545
4d
Mass/Charge

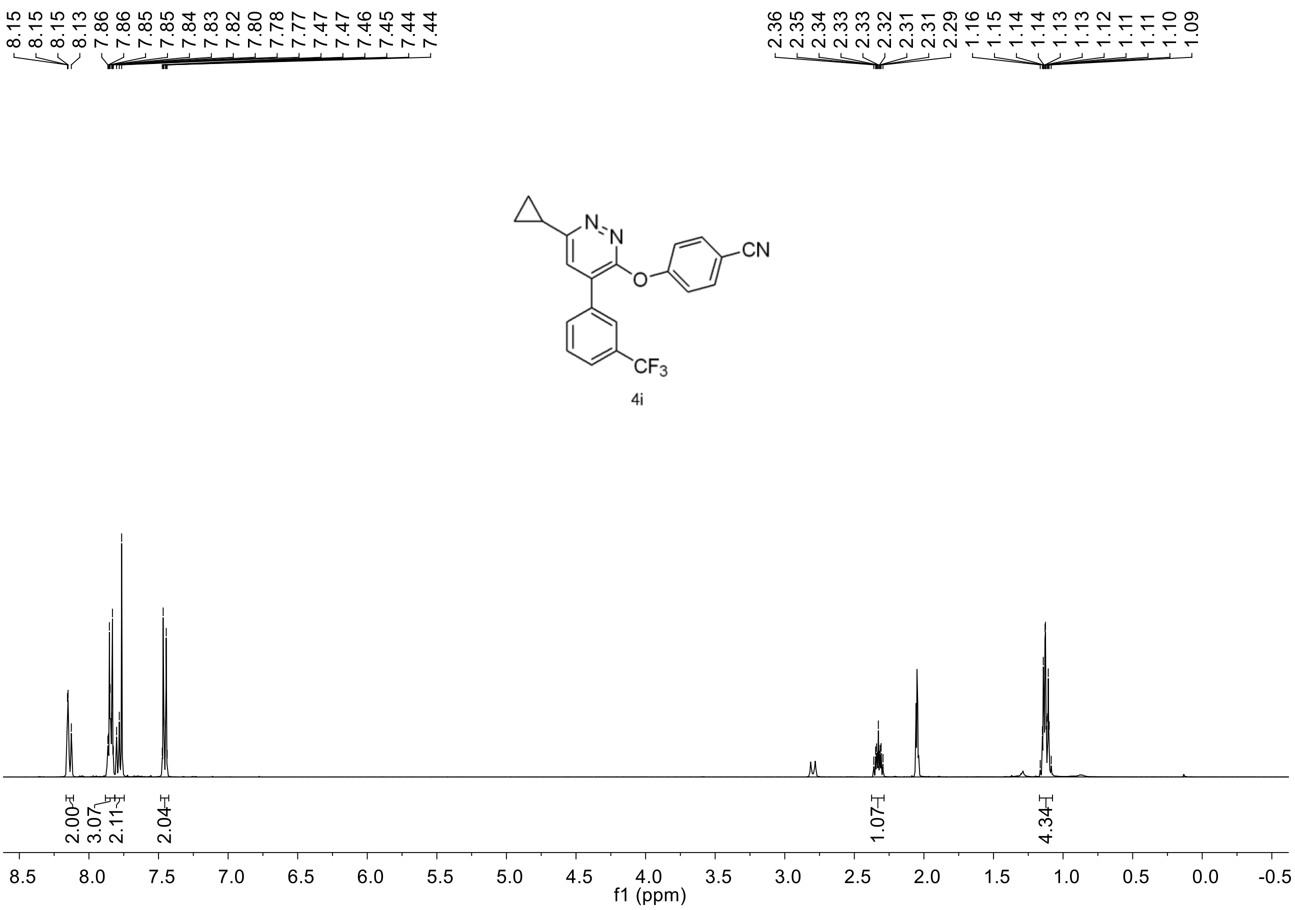
8.15
8.15
8.15
8.13
7.86
7.86
7.85
7.85
7.84
7.83
7.82
7.80
7.78
7.77
7.47
7.47
7.46
7.45
7.44
7.44
2.36
2.35
2.34
2.33
2.33
2.32
2.31
2.31
2.29
1.16
1.15
1.14
1.14
1.13
1.13
1.12
1.11
1.11
1.10
1.09
CN
O
N
N
CF3
4i
2.00
3.07
2.11
2.04
1.07
4.34
8.5 8.0 7.5 7.0 6.5 6.0 5.5 5.0 4.5 4.0 3.5 3.0 2.5 2.0 1.5 1.0 0.5 0.0 -0.5
f1 (ppm)

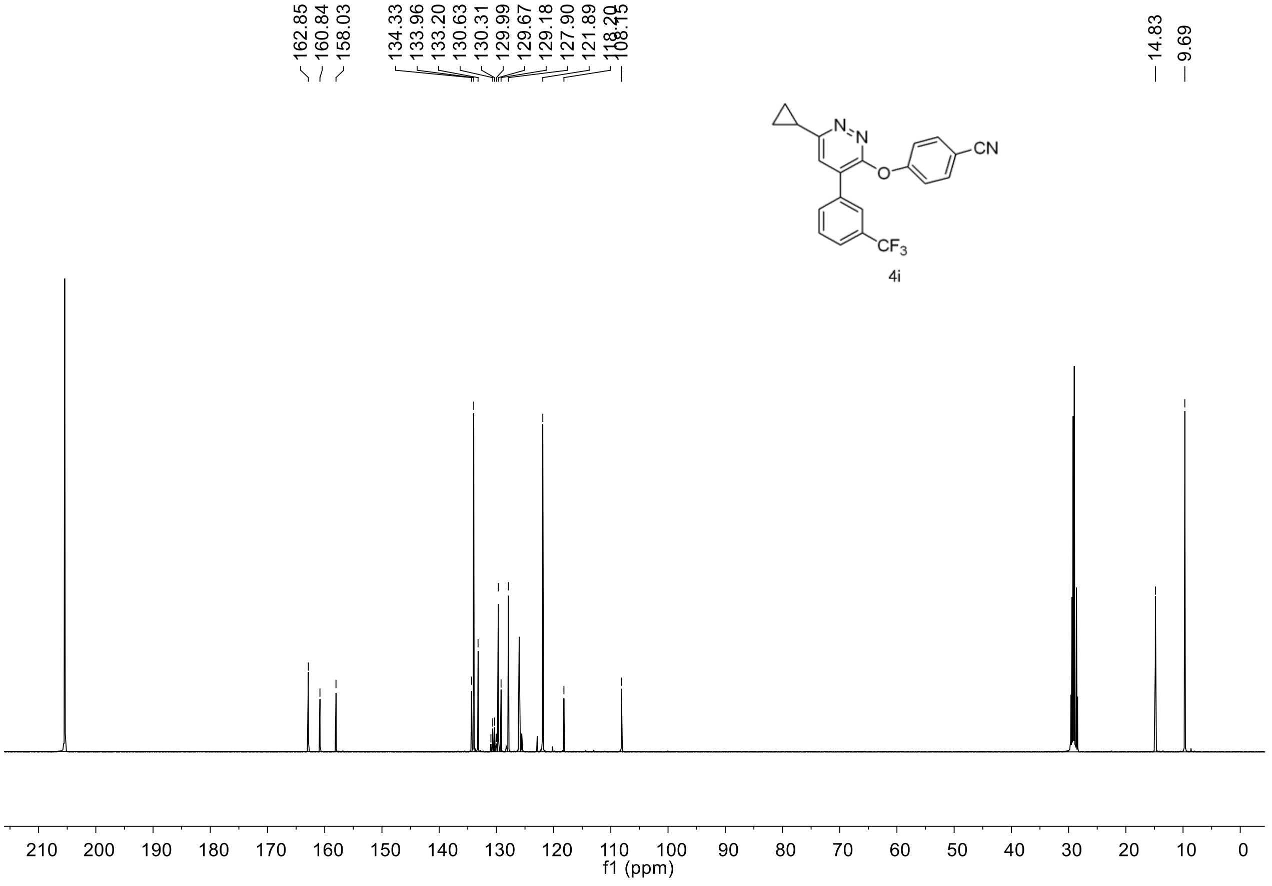
162.85
160.84
158.03
134.33
133.96
133.20
130.63
130.31
129.99
129.67
129.18
127.90
121.89
118.20
108.15
14.83
9.69
CN
O
N
N
CF3
4i
210 200 190 180 170 160 150 140 130 120 110 100 90 80 70 60 50 40 30 20 10 0
f1 (ppm)

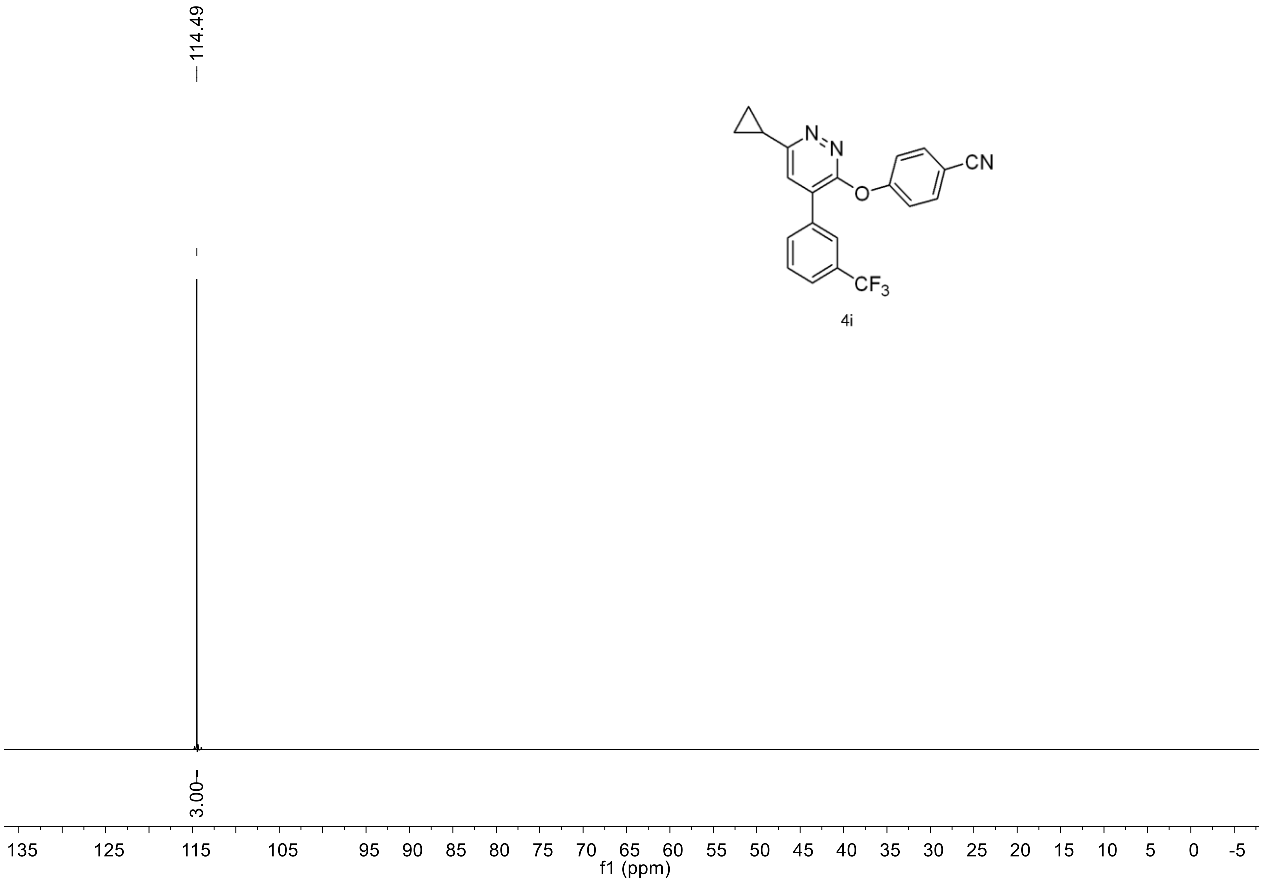
114.49
CN
O
N
N
CF3
4i
3.00
135 125 115 105 95 90 85 80 75 70 65 60 55 50 45 40 35 30 25 20 15 10 5 0 -5
f1 (ppm)

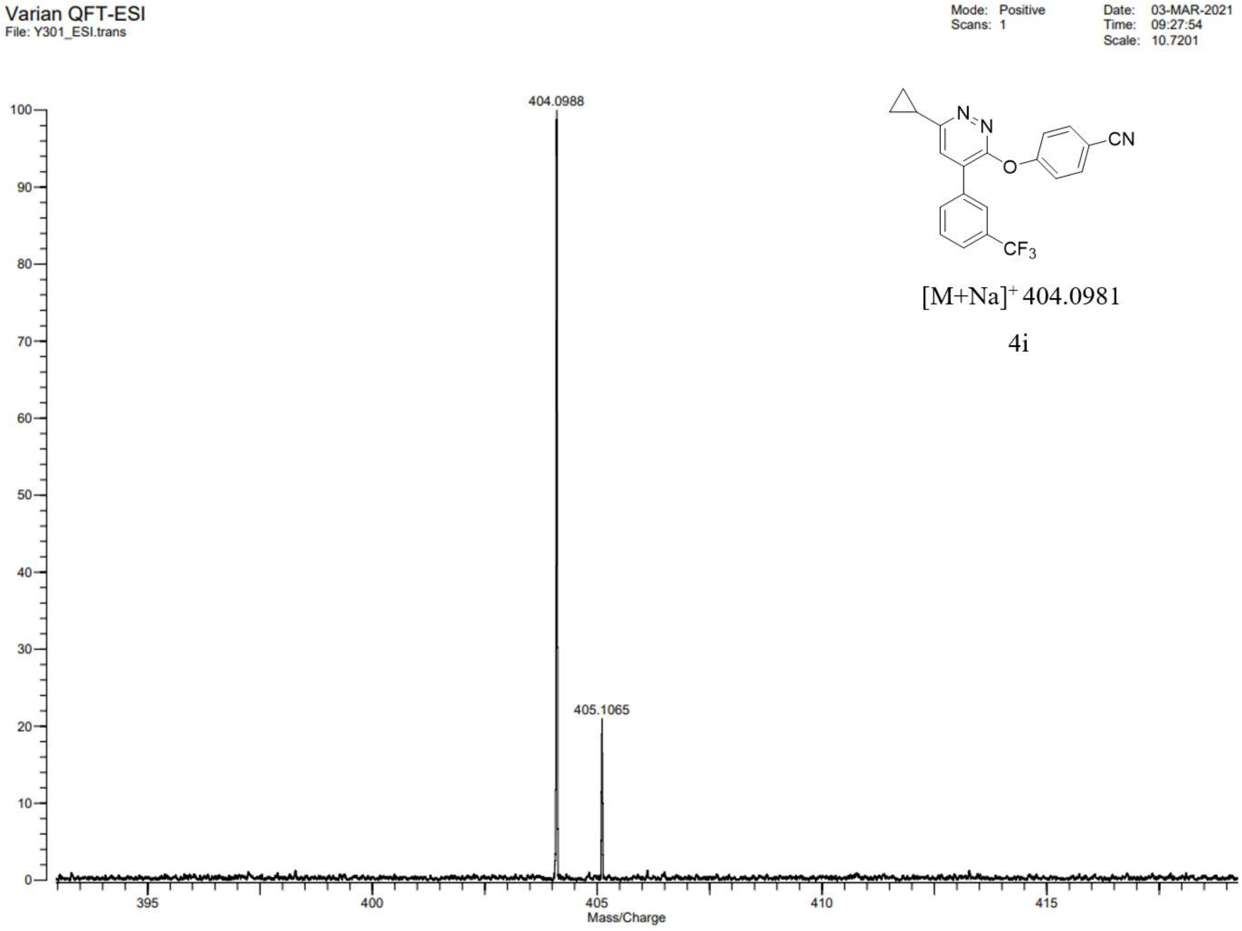
Varian QFT-ESI
File: Y301_ESI.trans
Mode: Positive
Scans: 1
Date: 03-MAR-2021
Time: 09:27:54
Scale: 10.7201
404.0988
405.1065
CN
O
N
N
CF3
[M+Na]+ 404.0981
4i
100 90 80 70 60 50 40 30 20 10 0
395 400 405 410 415
Mass/Charge

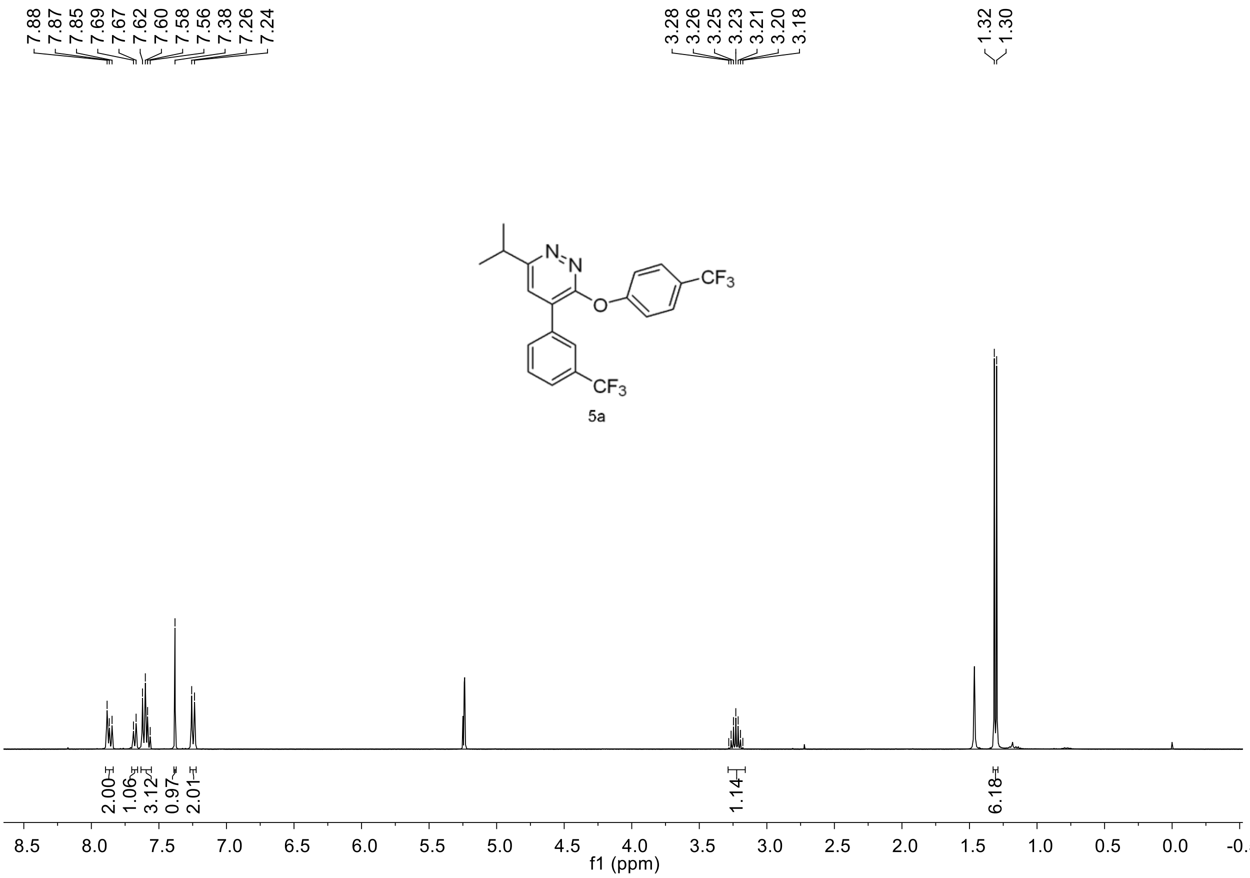
7.88 7.87 7.85 7.69 7.67 7.62 7.60 7.58 7.56 7.38 7.26 7.24
3.28 3.26 3.25 3.23 3.21 3.20 3.18
1.32 1.30
N
N
O
CF3
CF3
5a
2.00 1.06 3.12 0.97 2.01
1.14
6.18
8.5 8.0 7.5 7.0 6.5 6.0 5.5 5.0 4.5 4.0 3.5 3.0 2.5 2.0 1.5 1.0 0.5 0.0 -0.5
f1 (ppm)

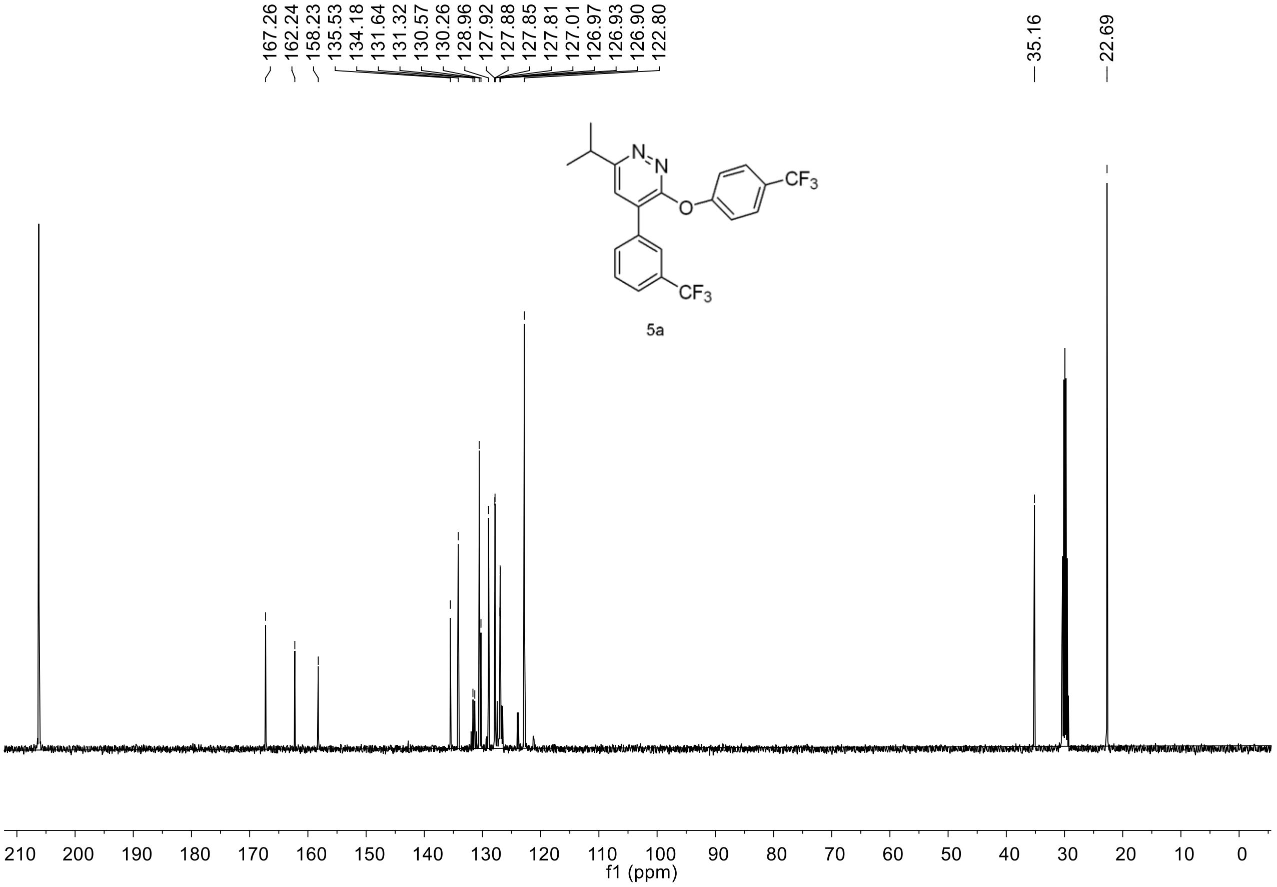
167.26 162.24 158.23 135.53 134.18 131.64 131.32 130.57 130.26 128.96 127.92 127.88 127.85 127.81 127.01 126.97 126.93 126.90 122.80
35.16
22.69
N
N
O
CF3
CF3
5a
210 200 190 180 170 160 150 140 130 120 110 100 90 80 70 60 50 40 30 20 10 0
f1 (ppm)

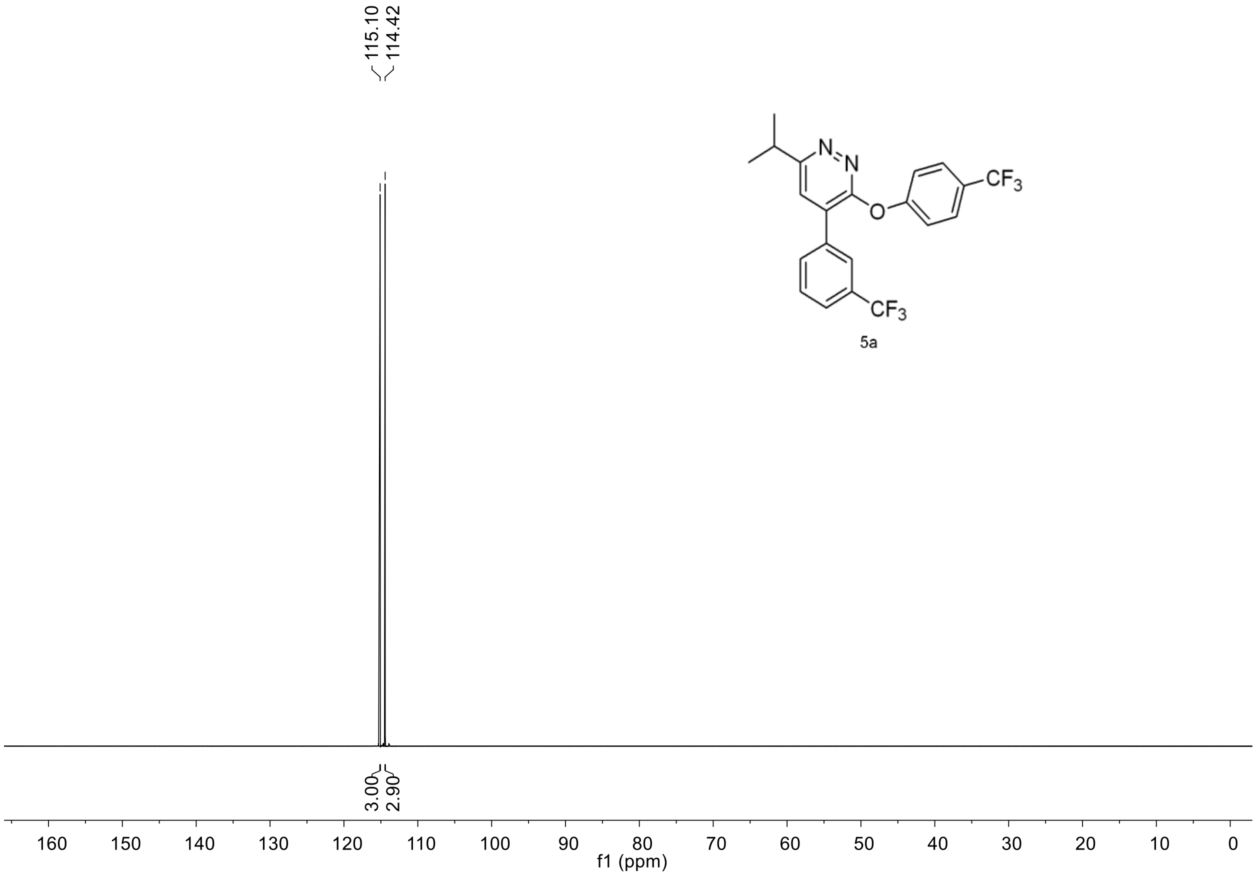
115.10
114.42
5a
3.00
2.90
160 150 140 130 120 110 100 90 80 70 60 50 40 30 20 10 0
f1 (ppm)

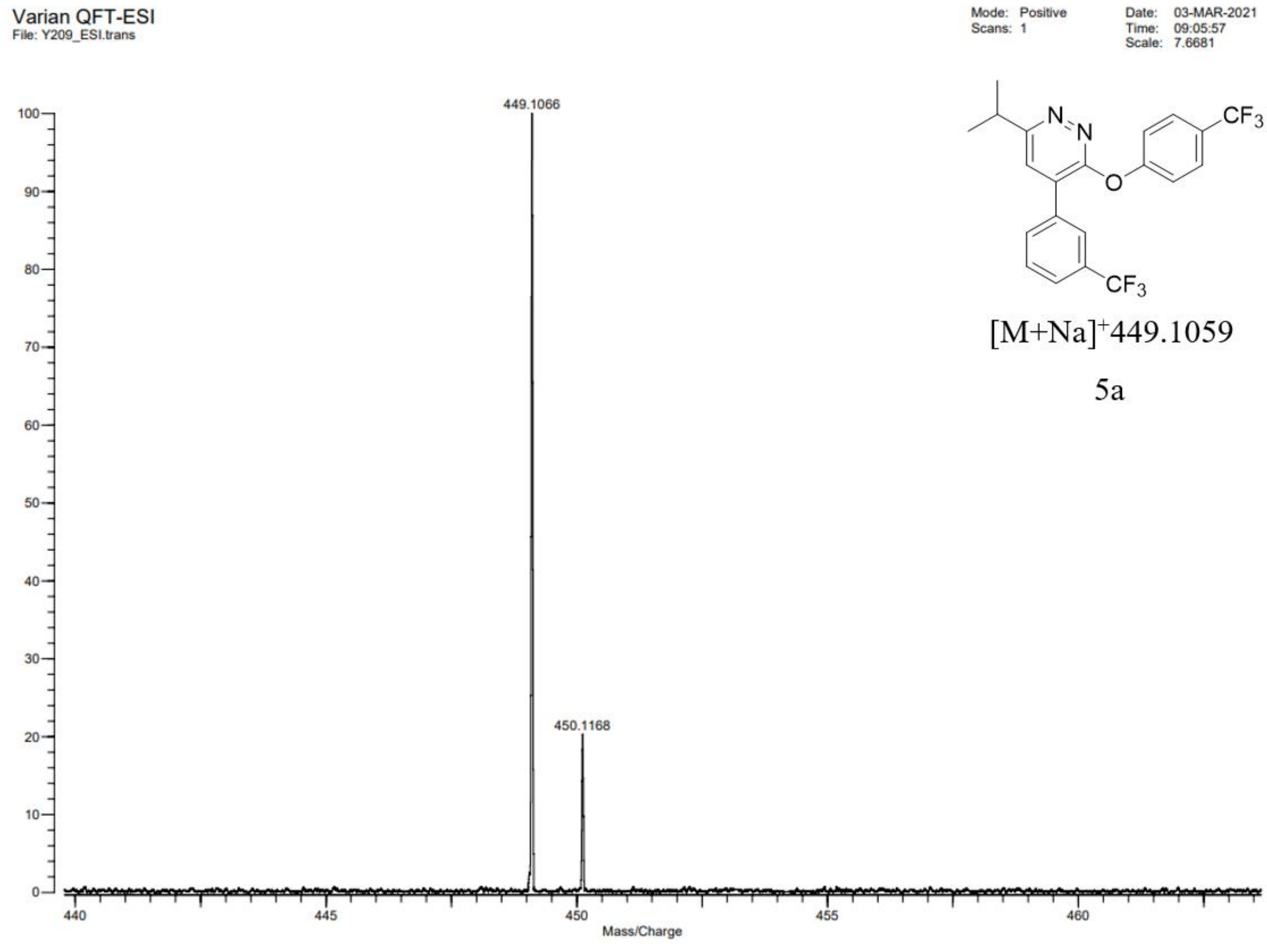
Varian QFT-ESI
File: Y209_ESI.trans
Mode: Positive
Scans: 1
Date: 03-MAR-2021
Time: 09:05:57
Scale: 7.6681
449.1066
450.1168
[M+Na]+449.1059
5a
Mass/Charge

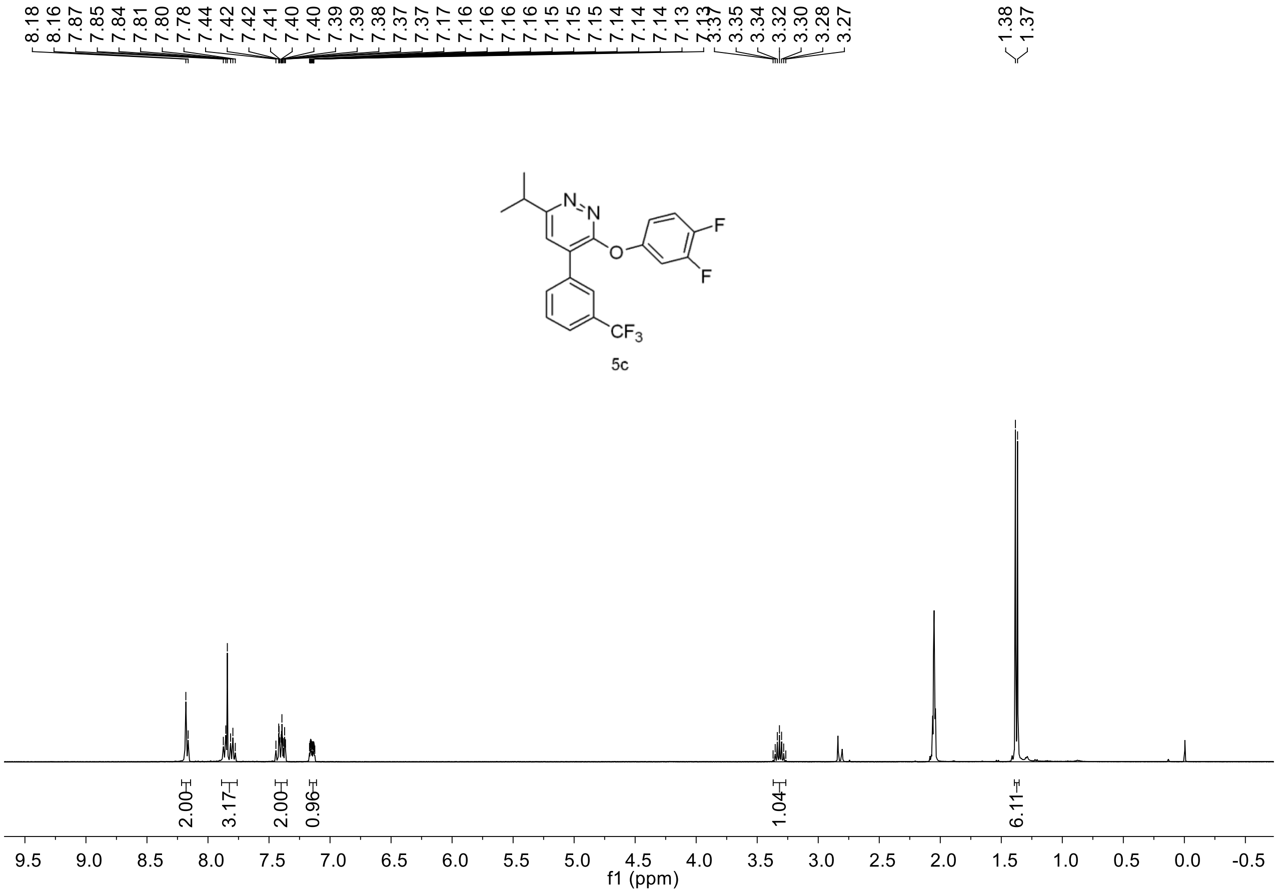
8.18 8.16 7.87 7.85 7.84 7.81 7.80 7.78 7.44 7.42 7.42 7.41 7.40 7.39 7.39 7.38 7.37 7.37 7.17 7.16 7.16 7.16 7.15 7.15 7.15 7.14 7.14 7.14 7.13 7.13 3.37 3.35 3.34 3.32 3.30 3.28 3.27
1.38 1.37
N
N
O
F
F
CF_3
5c
2.00 3.17 2.00 0.96 1.04 6.11
9.5 9.0 8.5 8.0 7.5 7.0 6.5 6.0 5.5 5.0 4.5 4.0 3.5 3.0 2.5 2.0 1.5 1.0 0.5 0.0 -0.5
f1 (ppm)

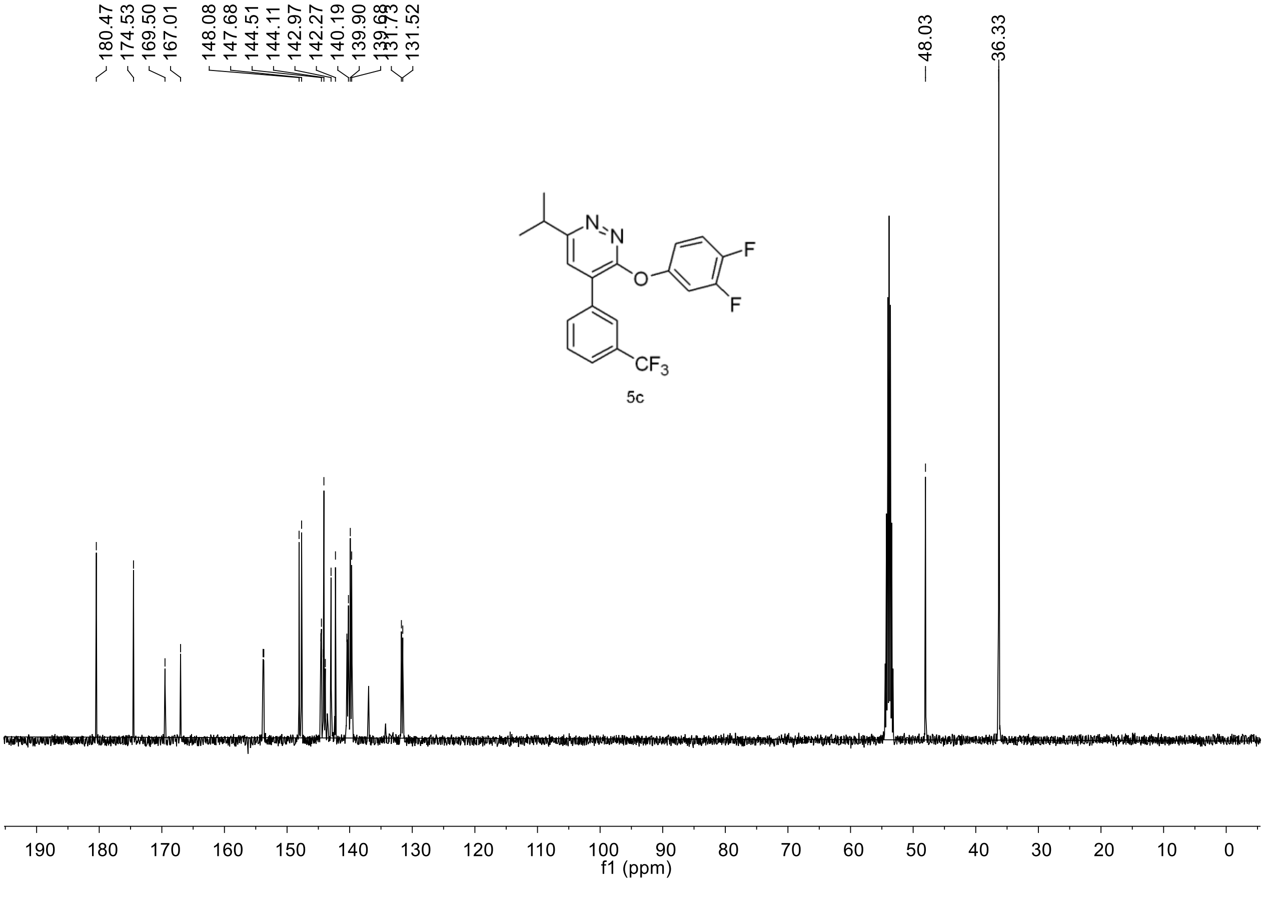
180.47 174.53 169.50 167.01 148.08 147.68 144.51 144.11 142.97 142.27 140.19 139.90 139.68 131.73 131.52
48.03
36.33
N
N
O
F
F
CF_3
5c
190 180 170 160 150 140 130 120 110 100 90 80 70 60 50 40 30 20 10 0
f1 (ppm)

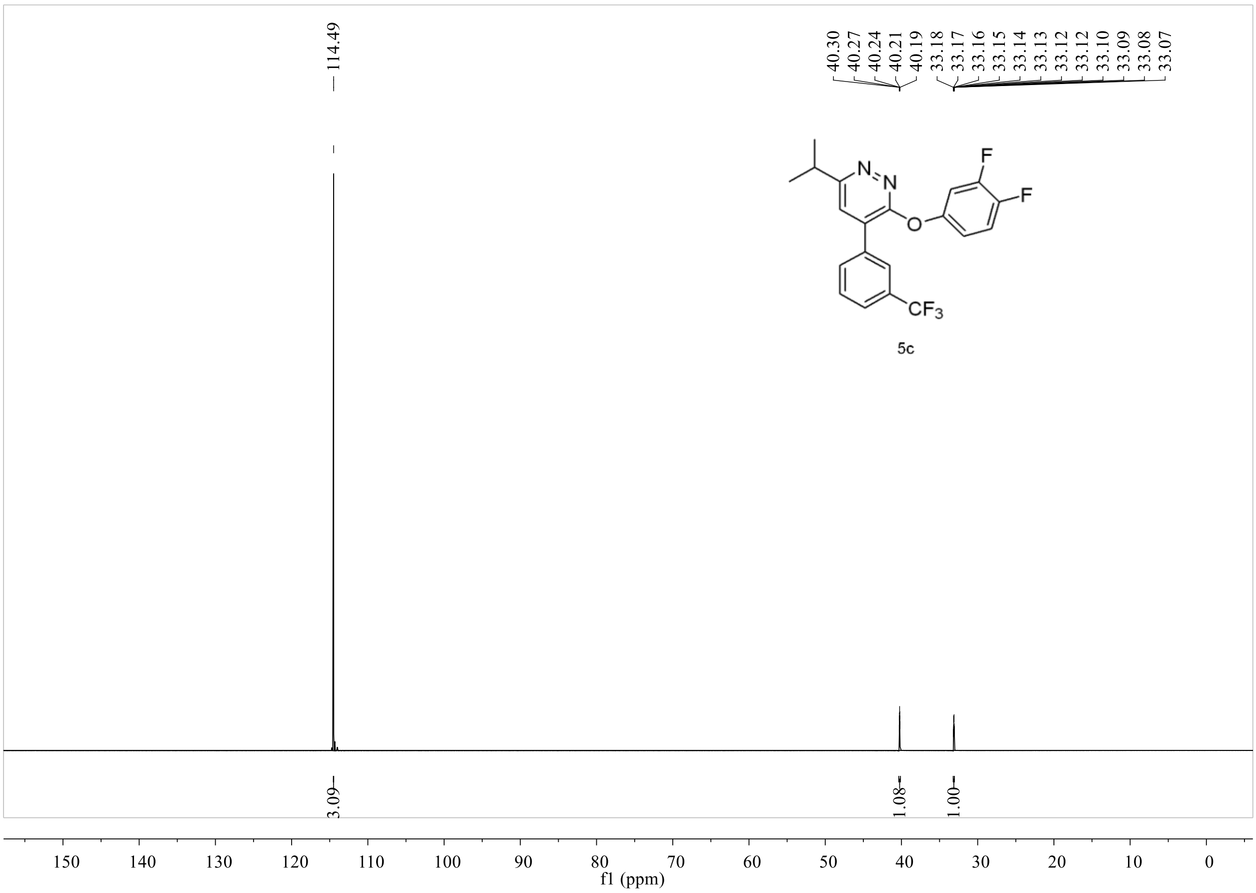

114.49
40.30
40.27
40.24
40.21
40.19
33.18
33.17
33.16
33.15
33.14
33.13
33.12
33.12
33.10
33.09
33.08
33.07
5c
3.09
1.08
1.00
150
140
130
120
110
100
90
80
70
60
50
40
30
20
10
0
f1 (ppm)

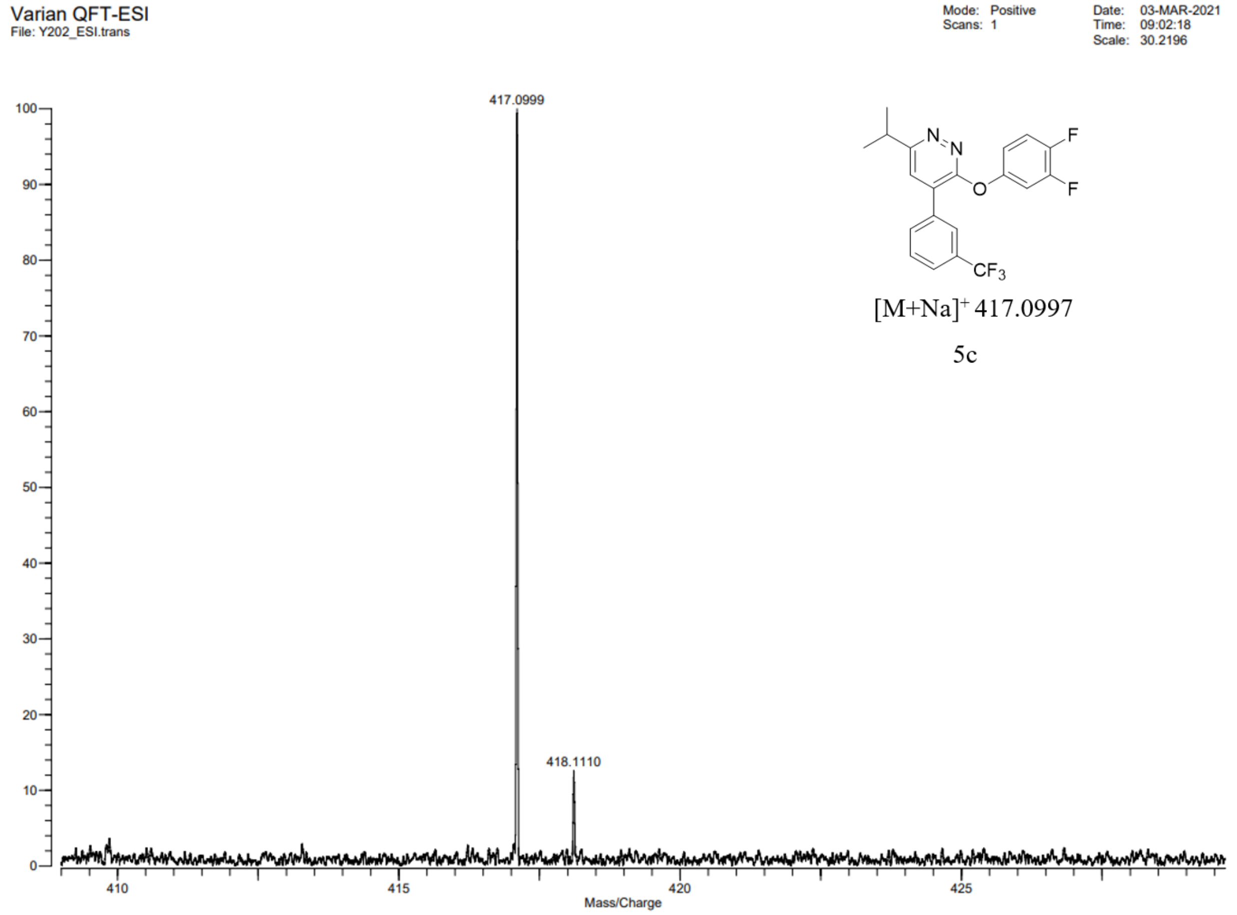

Varian QFT-ESI
File: Y202_ESI.trans
Mode: Positive
Scans: 1
Date: 03-MAR-2021
Time: 09:02:18
Scale: 30.2196
417.0999
418.1110
[M+Na]+ 417.0997
5c
Mass/Charge

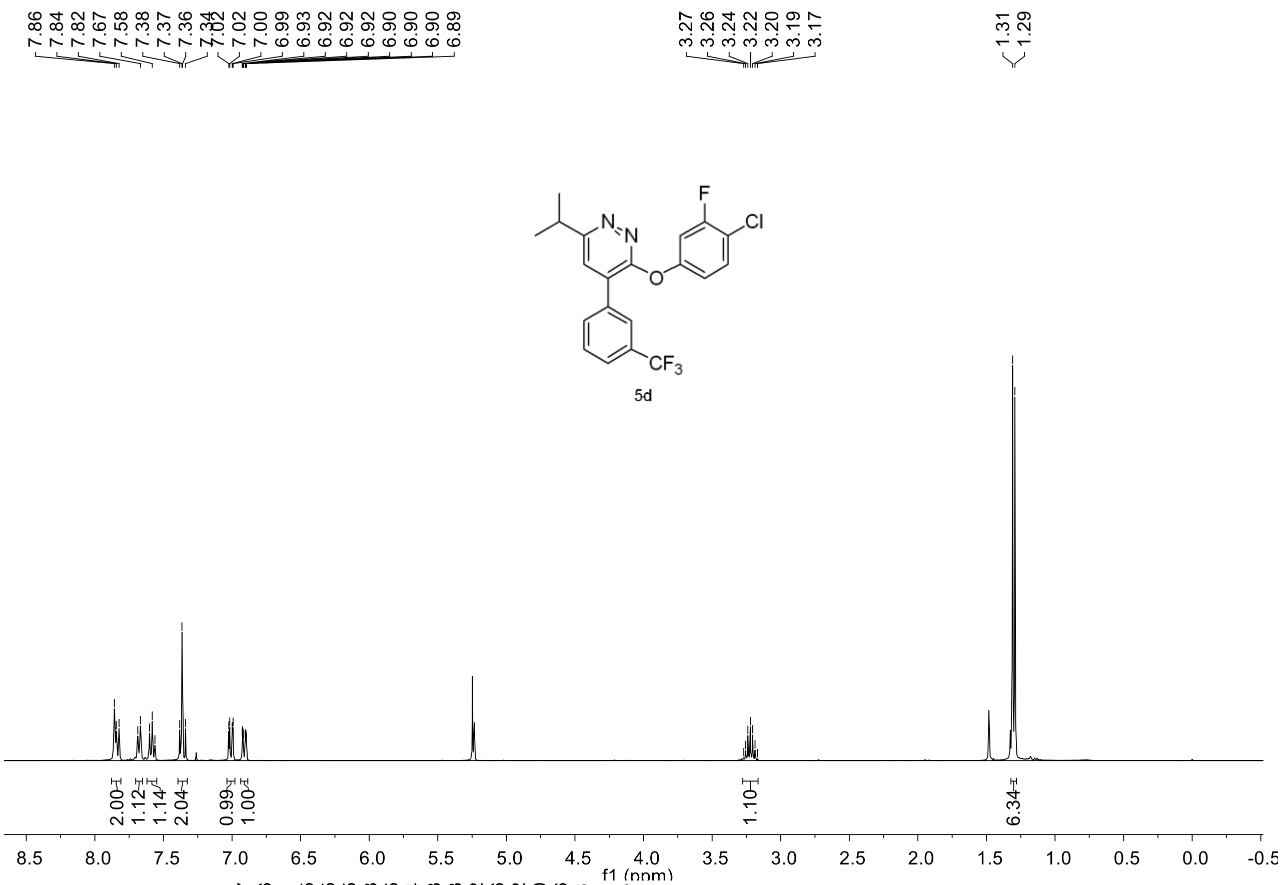
7.86
7.84
7.82
7.67
7.58
7.38
7.37
7.36
7.34
7.02
7.02
7.00
6.99
6.93
6.92
6.92
6.92
6.90
6.90
6.90
6.89
3.27
3.26
3.24
3.22
3.20
3.19
3.17
1.31
1.29
F
Cl
N
N
O
CF3
5d
2.00
1.12
1.14
2.04
0.99
1.00
1.10
6.34
f1 (ppm)

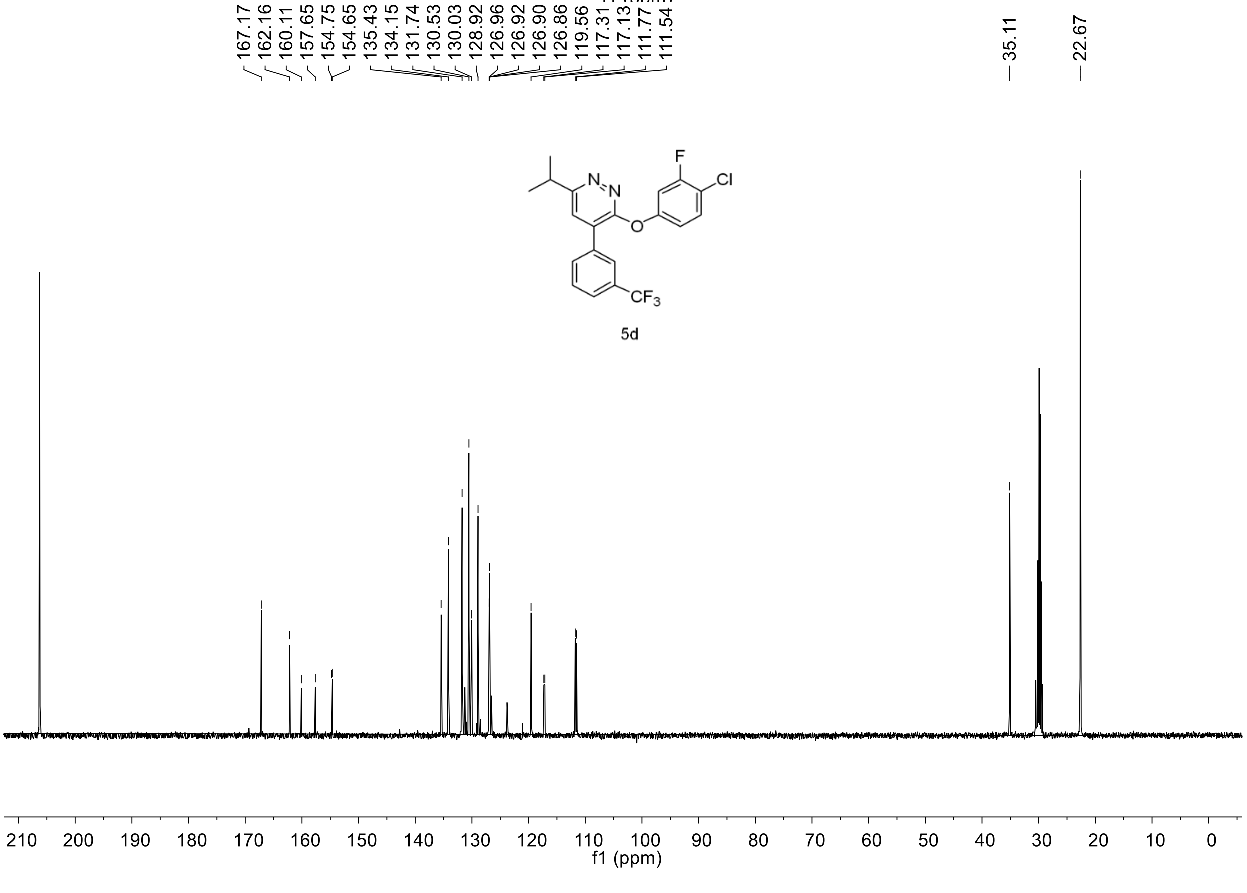
167.17
162.16
160.11
157.65
154.75
154.65
135.43
134.15
131.74
130.53
130.03
128.92
126.96
126.92
126.90
126.86
119.56
117.31
117.13
111.77
111.54
35.11
22.67
F
Cl
N
N
O
CF3
5d
f1 (ppm)

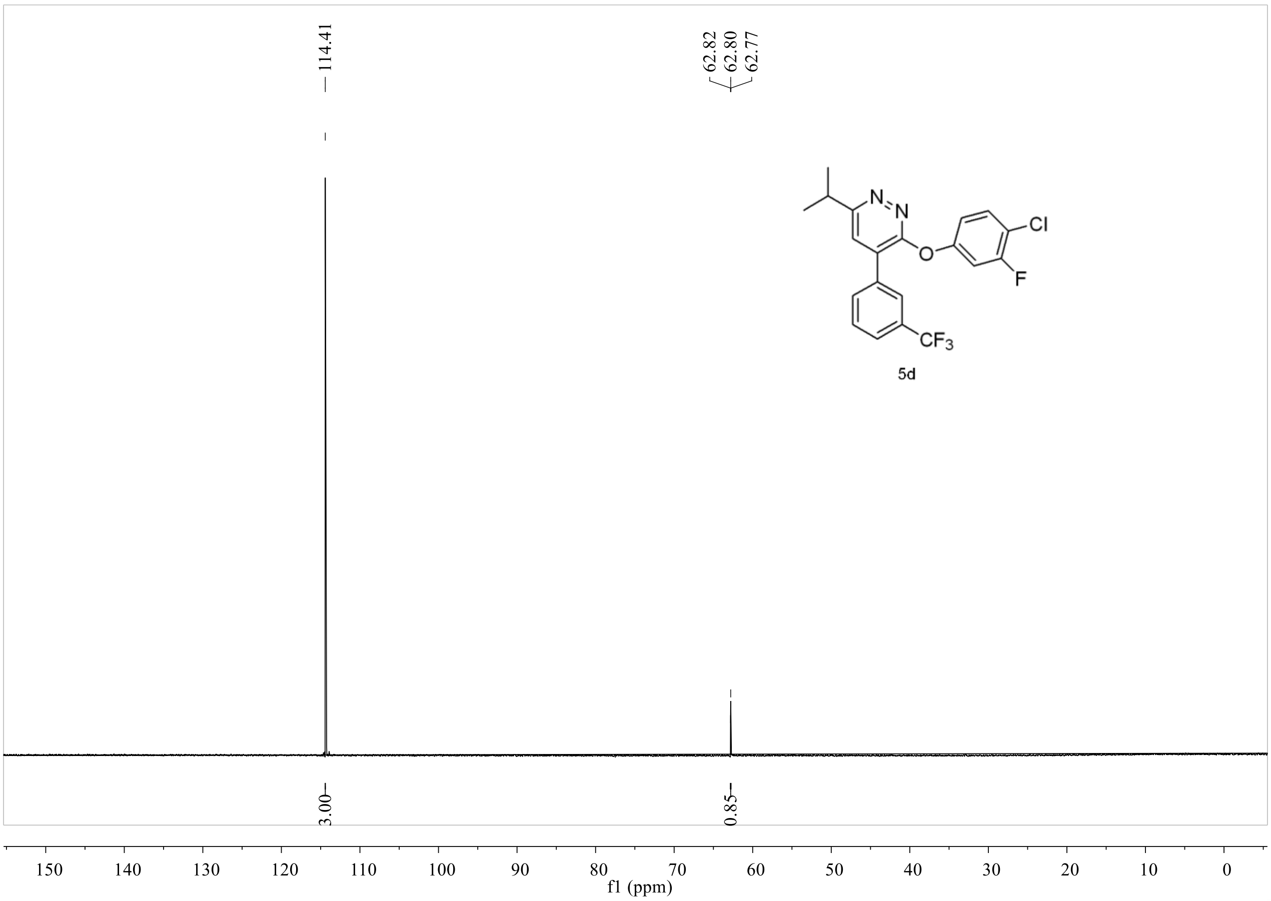
114.41
62.82
62.80
62.77
5d
3.00
0.85
150
140
130
120
110
100
90
80
70
60
50
40
30
20
10
0
f1 (ppm)

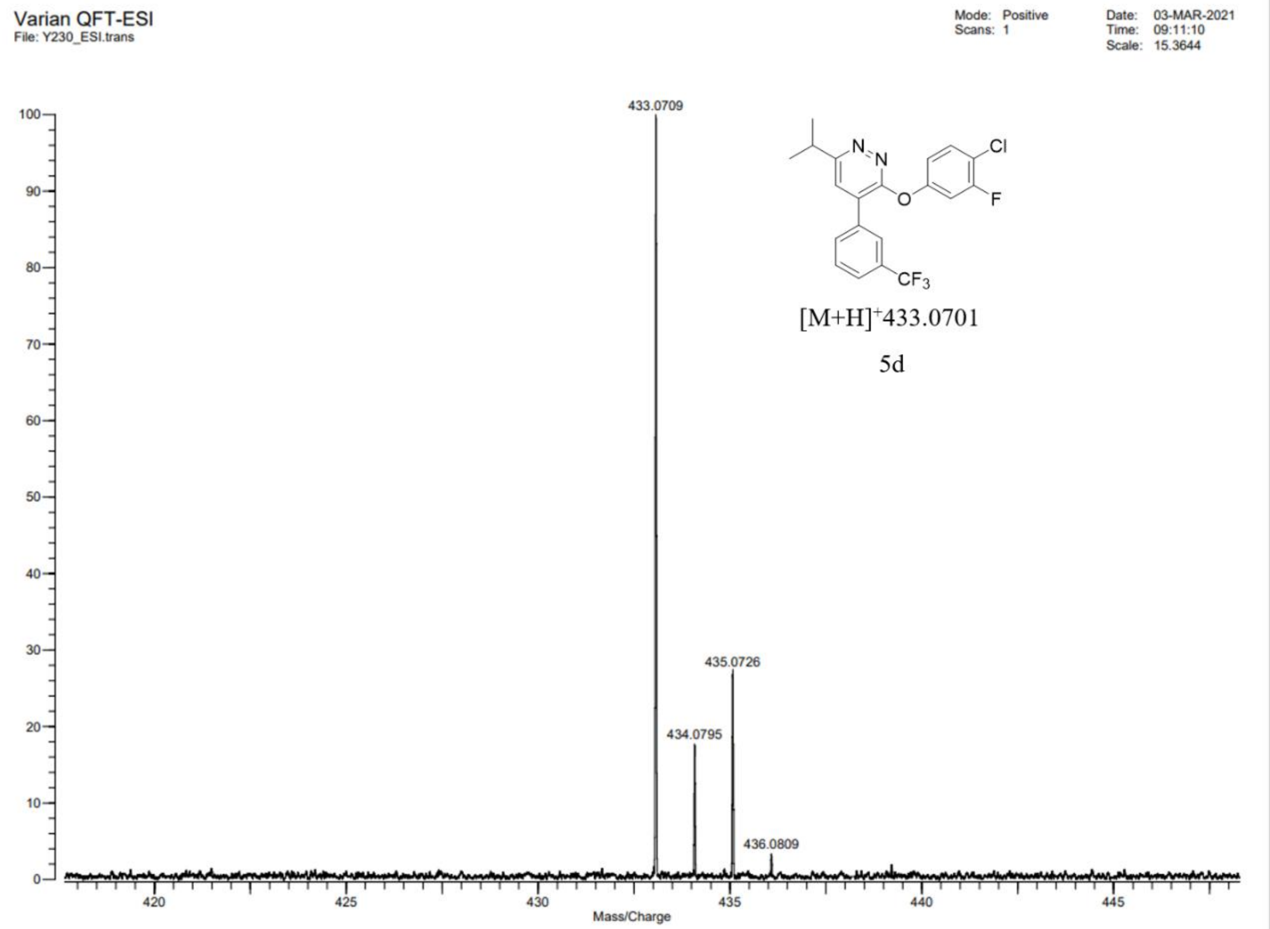
Varian QFT-ESI
File: Y230_ESI.trans
Mode: Positive
Scans: 1
Date: 03-MAR-2021
Time: 09:11:10
Scale: 15.3644
433.0709
434.0795
435.0726
436.0809
[M+H]+433.0701
5d
Mass/Charge
